# A Diborylcarbazolyl Ligand for Stabilizing Low-Coordinate and Low-Valent Metal Complexes

**DOI:** 10.1021/acs.inorgchem.5c05146

**Published:** 2026-02-23

**Authors:** Yen-Hua Lee, Wei-Chieh Chang, Hsuan-Wen Fu, Han-Jung Li, Ting-Shen Kuo, Tzuhsiung Yang, Hsueh-Ju Liu

**Affiliations:** a Department of Applied Chemistry, 34914 National Yang Ming Chiao Tung University, 1001 Daxue Rd, East District, Hsinchu 300093, Taiwan; b Department of Chemistry, 34879 National Taiwan Normal University, Taipei 116059, Taiwan; c Department of Chemistry, 34881 National Tsing Hua University, Hsinchu 300044, Taiwan; d Center for Emergent Functional Matter Science, 34914 National Yang Ming Chiao Tung University, 1001 Daxue Rd, East District, Hsinchu 300093, Taiwan

## Abstract

We report the synthesis of a novel ligand L (3,6-di-*tert*-butyl-1,8-bis­(dimesitylboryl)­carbazolide) based on a carbazolyl scaffold bearing two sterically demanding boryl groups at the 1,8-positions. This diborylcarbazolyl framework effectively supports a range of transition metal complexes, including two-coordinate halide complexes **LM** ^**II**^ **Cl** [M = Fe (**2**), Co (**3**), and Zn (**4**)], trinuclear complex (**LCr**)**_2_** (**CrCl**_**4**_) (**5**), and pseudo-one-coordinate complexes **LM** ^**I**^ [M = Ni (**6**) and Cu (**7**)]. Coinage metal complexes derived from L exhibit a preference for two-coordinate geometries, as demonstrated by the isolation of **LMPPh**_**3**_ [M = Ag (**8**) and Au (**9**)] and **LCu**(**DMAP**) (**10**).

## Graphical abstract

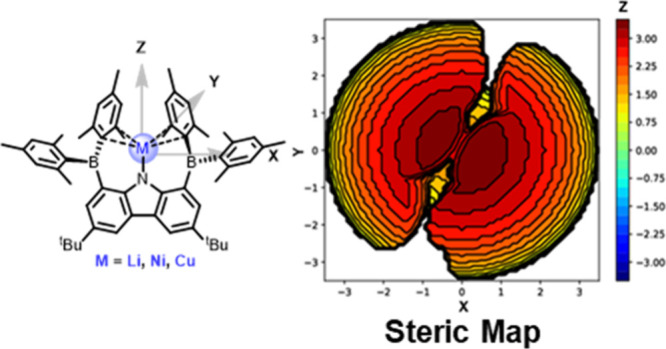

## Introduction

Low-coordinate metal complexes, typically those with coordination numbers of three or fewer,^1^ often display unique electronic structures and/or unusual reactivity due to their highly unsaturated metal centers. This coordinative and electronic unsaturation frequently leads to the discovery of novel bonding motifs and reactivity patterns, enabling their widespread application in small-molecule activation and catalysis.^1,2^ A landmark example is the isolation of quintuple-bonded Cr­(I)–Cr­(I) complexes supported by sterically demanding terphenyl^3^ and amidinate^4^ ligands, which provided fundamental insights into multiple bonding in low-coordinate, low-valent transition metal systems. Related studies and reactivity investigations of quintuple-bonded complexes have since been reported by Tsai, Theopold, and others.^5–8^ In the main-group arena, Jones achieved the isolation of stable Mg­(I) complexes featuring Mg–Mg bonds by employing β-diketiminate ligands with bulky aryl substituents,^9^ which were later shown to serve as effective reducing agents in various reactions.^10^

Accordingly, ligand design has emerged as a central strategy for stabilizing such reactive low-coordinate species, with sterically encumbered frameworks playing a particularly important role. The use of sterically demanding ligands—such as terphenyls,^11^ β-diketiminates,^12^ amides, carbazole, and N-heterocyclic carbenes^13,14^ —has proven effective for stabilizing such reactive species by providing kinetic protection against undesired ligand association and oligomerization. Among these, the carbazolyl framework is particularly versatile, functioning both as a monodentate bulky ligand and as a platform for pincer ligands incorporating donor functionalities such as imines or phosphines (Figure 1a).^15^ Notably, Aldridge and co-workers demonstrated that carbazolyl frameworks offer more sterically protected cavities than terphenyl analogues when bearing equivalent bulky substituents, based on comparative steric and angular analyses.^16^ Building on this, various research groups have introduced diverse substituents onto the carbazolyl scaffold (Figure 1b), resulting in an expanding library of low-coordinate transition metal complexes.^17–20^ Despite these advances, one notable gap in carbazole-based ligand design is the limited incorporation of Lewis acidic substituents. This gap presents an opportunity to combine the steric rigidity and structural robustness of carbazolyl frameworks with Lewis acidic groups to promote metal–Lewis acid cooperativity.

**Figure 1:**
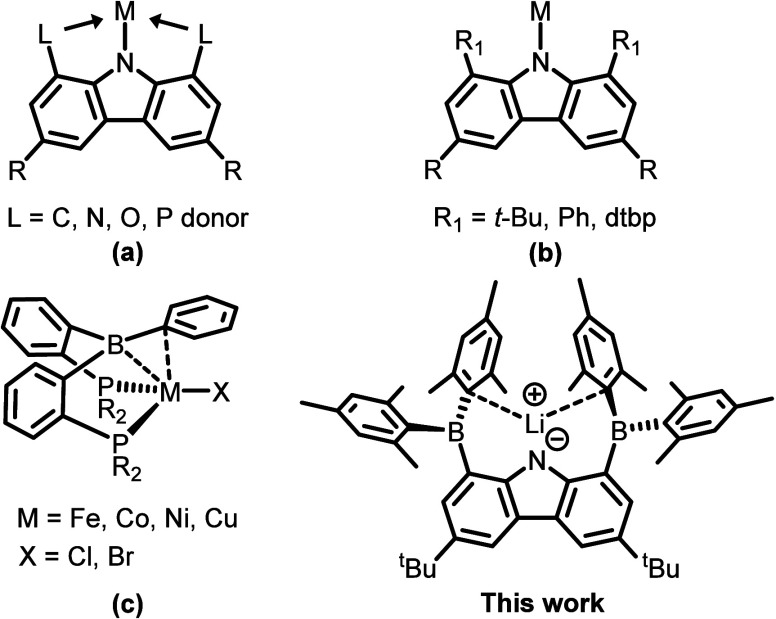
Selected examples of different types of ligands: (a) pincer carbazolyl ligands; (b) bulky monodentate carbazolyl ligands (dtbp = 3,5-di-*tert*-butylphenyl); (c) bis­(phosphine) borane ligands.

Specifically, ligands bearing pendant borane or aluminum moieties as Lewis acids, paired with low-valent, electron-rich metal centers (as Lewis bases), have shown cooperative substrate activation and catalytic activity.^21,22^ Over the past decade, boron-containing ligand frameworks, especially those bearing phosphine side arms, have emerged as key motifs for cooperative catalysis with low-valent metals (Figure 1c).^23–27^ In many of these systems, M→B (Z-type) interactions are observed, in which the electron density from the metal is shared with the Lewis acidic boron, enhancing complex stability and tuning reactivity.

Our initial design strategy was to incorporate Lewis acidic boryl groups at the 1,8-positions of a carbazolyl scaffold with the expectation that they might engage in M→B (Z-type) interactions to stabilize low-valent metal centers. However, in the course of this study, we did not observe such a Z-type bonding. Instead, structural analyses revealed that the mesityl substituents on the boryl groups provide unexpected but effective stabilization of the low-coordinate metal centers. These results demonstrate a new role for boryl substituents in low-coordinate metal chemistry. Herein, we report the synthesis of a carbazole-based ligand bearing two sterically encumbered boryl groups (BMes_2_CBz, L), along with its coordination to a series of low-coordinate transition metal complexes.

## Results and Discussion

### Ligand Synthesis and Characterizations

The synthesis of **BrCBzLi**, **LLi** (**1**) (lithium 3,6-di-*tert*-butyl-1,8-bis­(dimesitylboryl)­carbazolide), and **LH** (**1**-**H**) (3,6-di-*tert*-butyl-1,8-bis­(dimesitylboryl)­carbazole) is outlined in Scheme 1. Starting from the precursor **BrCBzH** (1,8-dibromo-3,6-di-*tert*-butylcarbazole),^28^ deprotonation with *n*-BuLi in pentane afforded the corresponding lithium carbazolide, which was further crystallized from THF to give **BrCBzLi** in 66% yield. In contrast to conventional 1,8-functionalization strategies based on 9-silylated carbazole precursors,^29^ this THF-coordinated lithium carbazolide **BrCBzLi** was also proved to be an effective precursor for subsequent functionalization. Treatment of **BrCBzLi** with 4 equiv of *n*-BuLi promoted lithium–bromine exchange, followed by 1,8-diborylation with dimesitylboron fluoride to afford **LLi** (**1**) as a red solid in 45% yield. Notably, attempts to directly functionalize **BrCBzH** using excess *n*-BuLi (5 equiv) in diethyl ether—presumed to generate the lithium–bromine exchange intermediate—followed by borylation afforded **LLi** (**1**) in only 14% yield. Compound **1** is highly sensitive to air and moisture and reacts with proton sources to give **LH** (**1**-**H**) almost quantitatively. Both compounds **1** and **1**-**H** are soluble in a variety of organic solvents, including THF, benzene, toluene, and pentane, and can be crystallized by slow evaporation from diethyl ether. The ^1^H NMR spectra confirm the symmetrical nature of **1** and **1**-**H** with respect to the central five-membered ring. In contrast, **1** reveals two distinct sets of resonances for the mesityl (Mes) substituents, whereas **1**-**H** shows only one set. This difference might arise from an interaction between the lithium center and inner Mes groups. The ^11^B­{^1^H} NMR spectra exhibit a single broad resonance at 71.3 (**1**) and 69.5 (**1**-**H**) ppm, consistent with chemical shifts observed in typical triarylborane environments,^30^ thus indicative of negligible interaction between the boron and nitrogen centers. Compound **1** crystallizes in the triclinic P1̅ space group, with two crystallographically independent but nearly identical molecules in the unit cell. The crystal structure reveals a monomeric species, in which the lithium atom is nestled within the central “pocket” of the ligand, forming a contact ion pair with the carbazole nitrogen atom with a Li–N bond length of 1.900 (9) Å. The lithium center is further stabilized through secondary Li···C interactions with the flanking Mes groups, with distances ranging from 2.384(9) to 2.538(9) Å, which are comparable to those reported in other bulky alkali carbazolide systems.^16,31,32^ Each boron center adopts a trigonal planar geometry, as evidenced by a sum of bond angles of 360° and the absence of significant short contacts (>3.0 Å) with lithium or nitrogen atoms. Notably, unlike conventional carbazolyl frameworks where steric substituents are appended directly to the carbazolyl backbone, the Mes groups in this system are attached via boryl linkers, providing enhanced conformational flexibility. This structural feature allows the Mes rings to tilt inward toward the central pocket, positioning their π-systems in proximity to the metal center. This inward tilt is expected to contribute to stabilization through aryl–metal interactions, a distinguishing feature of this ligand design.

**Scheme 1:**
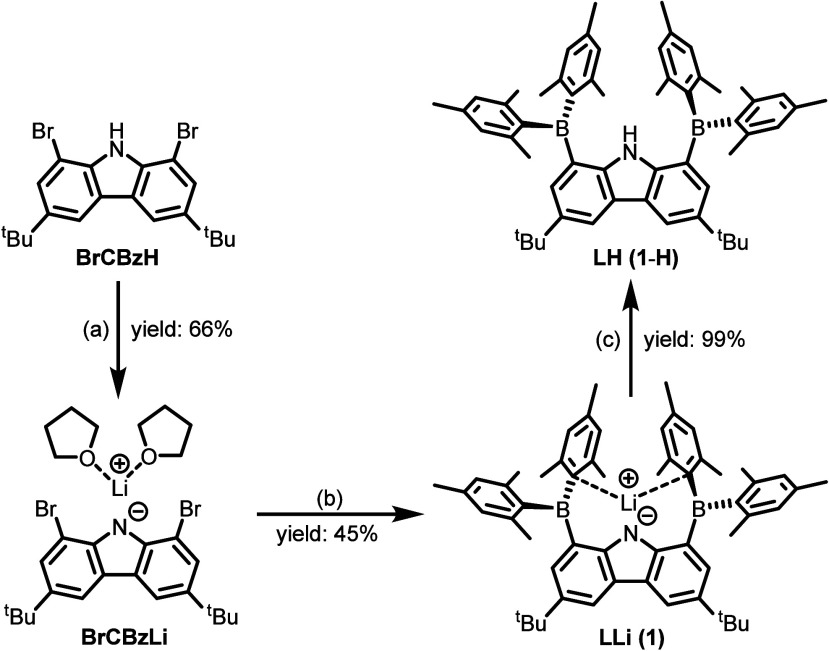
Synthesis of **BrCBzLi**, **LLi** (**1**), and **LH** (**1**-**H**): (a) 1 equiv of *n*-BuLi in Pentane and Crystallization from THF; (b) 4 equiv of *n*-BuLi and 2.1 equiv of BF­(Mes)_2_ in Et_2_O; (c) Exposed to Air in Toluene

### Complexes Synthesis and Characterizations

With **LLi** (**1**) in hand as a suitable ligand precursor for metathesis reactions, we next explored the coordination behavior of BMes_2_CBz (L) toward a range of transition metal halides. These included first-row transition metals from Cr to Zn as well as the coinage metals Ag and Au. A variety of coordination geometries were observed across the resulting metal complexes, reflecting the influence of both the metal identity and oxidation state. Specifically, reactions of **LLi** (**1**) with FeCl_2_, CoCl_2_, and ZnCl_2_(THF) yielded monomeric, two-coordinate halide complexes of the type LM­(II)Cl [Fe (**2**), Co (**3**), and Zn (**4**)]. In contrast, treatment of **1** with an excess of CrCl_2_ led to the unexpected formation of a trinuclear complex, (**LCr**)**_2_** (**CrCl**_**4**_) (**5**), highlighting the distinct coordination chemistry of group VI metals with this ligand framework. For group 10 and 11 metals, coordination of L to Ni­(I) and Cu­(I) afforded pseudo-one-coordinate complexes LM­(I) [Ni (**6**) and Cu (**7**)]. Notably, the corresponding Ag­(I) and Au­(I) complexes were found to be thermally unstable. Further reaction with external donor ligands resulted in the formation of linear, two-coordinate complexes of the type LML′ [**8**–**10**], as discussed in detail below. Molecular structures of complexes **1**–**4** and **6**–**10** are shown in Figure 2 and in the Supporting Information.

**Figure 2:**
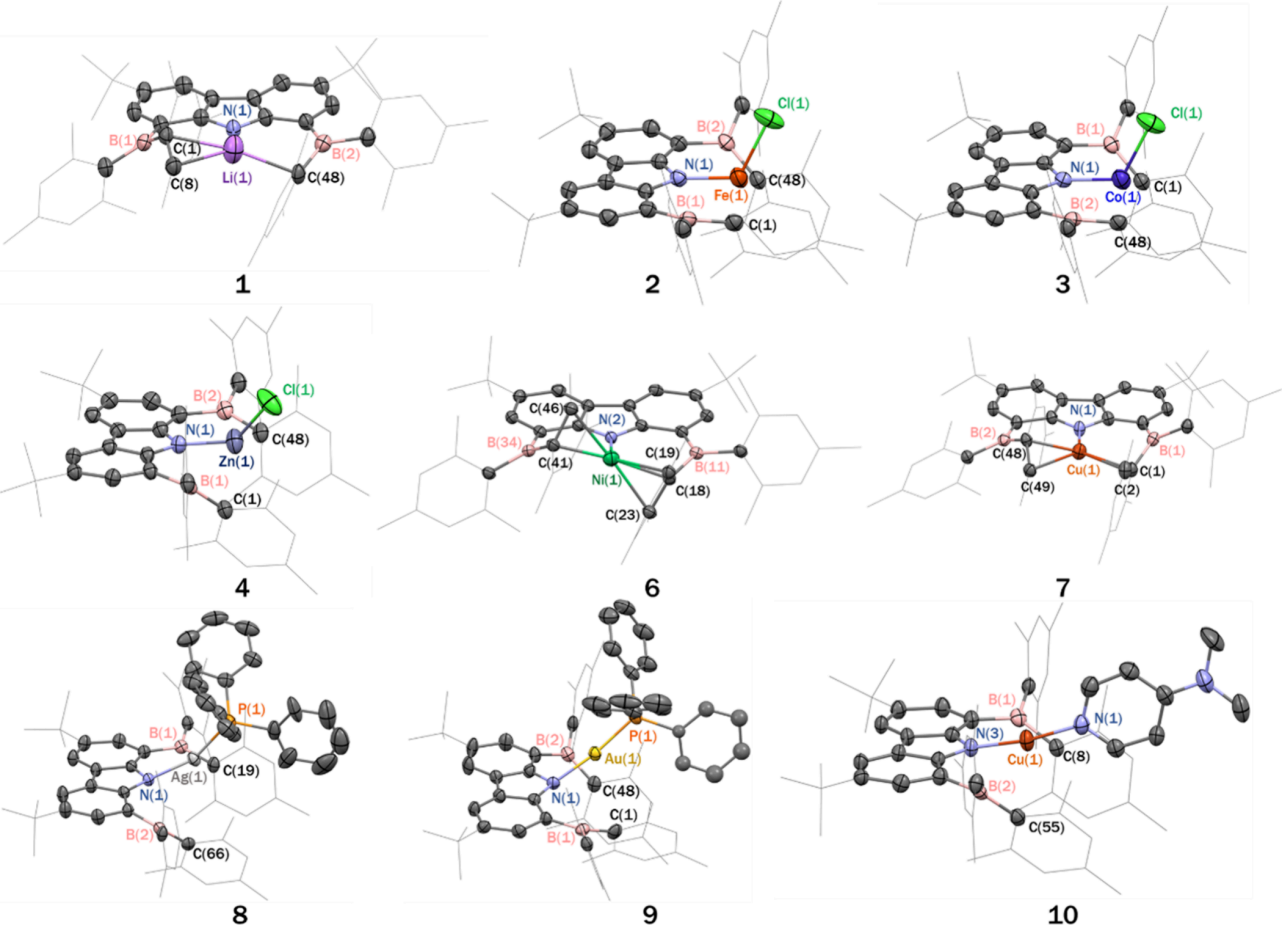
Thermal ellipsoid plots (50% probability surface) of the molecular structures of **1**–**4** and **6**–**10**. Hydrogen atoms, solvate molecules, and the disorder portions of the *t*-butyl and phenyl groups are omitted for clarity. For **1**, only one of the two independent molecules in the unit cell is displayed.

The reaction of **LLi** (**1**) with 1.3 equiv of FeCl_2_ in THF cleanly afforded **LFeCl** (**2**) as a reddish-brown solid in 95% yield. Similarly, treatment of **1** with CoCl_2_ (1.2 equiv) and ZnCl_2_(THF) (1.1 equiv) yielded **LCoCl** (**3**; yellow-brown) and **LZnCl** (**4**; orange-brown) in 99% and 94% yields, respectively (Scheme 2). All three complexes were crystallized by slow evaporation of their concentrated Et_2_O solutions, similar to the method used for **1**. The ^1^H NMR spectrum of complex **4** displays sharp, well-resolved resonances, characteristic of the rigid carbazolyl framework, consistent with the expected diamagnetic nature of a d^10^ Zn­(II) complex. In contrast, the spectra of complexes **2** and **3** exhibit broad and poorly resolved signals over a wide chemical shift range, indicative of paramagnetic behavior at 25 °C. Magnetic susceptibility measurements using the Evans method revealed effective magnetic moments (μ_eff_) of 5.5 μ_B_ for **2** and 4.5 μ_B_ for **3** at 25 °C,^33^ consistent with high-spin d^6^ (*S* = 2) and d^7^ (*S* = 3/2) configurations, respectively. Single-crystal X-ray diffraction studies revealed that complexes **2**–**4** are mononuclear and isostructural, each featuring a bent L–M–Cl linkage (Figure 2). Notably, no THF molecules are coordinated to the metal centers of these complexes despite the reactions being performed in THF, suggesting that the ligand framework provides effective steric shielding of the metal sites. The N_carbazole_–M and M–Cl bond lengths systematically decrease from Fe to Zn (see Table 1), consistent with decreasing covalent radii across the series (high-spin Fe: 1.52 Å; high-spin Co: 1.50 Å; Zn: 1.22 Å).^34^ The N_carbazole_–M–Cl angles are 119.37(7)° for **2**, 119.53(10)° for **3**, and 133.49 (9)° for **4**. These values reflect typical angular distortion observed in low-coordinate first-row complexes due to open-shell configurations and ligand–ligand repulsion.^35^ In addition to the primary N_carbazole_–M and M–Cl interactions, all three complexes exhibit secondary contacts between the metal centers and the flanking mesityl groups, with M···C_ipso_ distances ranging from 2.436(4) to 2.885(4) Å. These distances are significantly longer than the sum of the covalent radii for M–C bonds, suggesting weak to negligible aryl–metal interactions rather than formal coordination. These bond metrics are consistent with those observed in other structurally characterized two-coordinate complexes. Specifically, reported two-coordinate amido complexes exhibit M–N bond lengths ranging from 1.8532(13) to 1.9758(18) Å for Fe, 1.8179(14) to 1.880(2) Å for Co, and 1.8275(14) to 1.887(3) Å for Zn. Similar secondary M···C contacts have also been observed in these cases, spanning 2.4567(12)–2.809(2) Å for Fe, 2.393(2)–2.811(3) Å for Co, and 2.762(2)–2.824(3) Å for Zn.^19,20,36–41^ By comparison, terphenyl supported two-coordinate metal complexes display secondary M···C contacts in the ranges of 2.720(3)–3.004(3) Å for Fe, 2.679(2)–2.965(2) Å for Co, and 2.821(2)–3.025(2) Å for Zn, suggesting that any M···C interactions, if present, are likely to be very weak.^42–46^

**Scheme 2:**
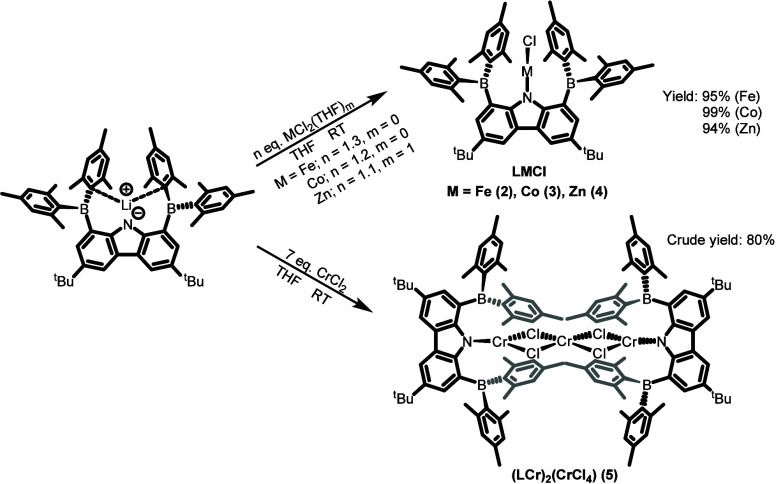
Synthesis of Complexes **2**–**5**

**Table 1:**
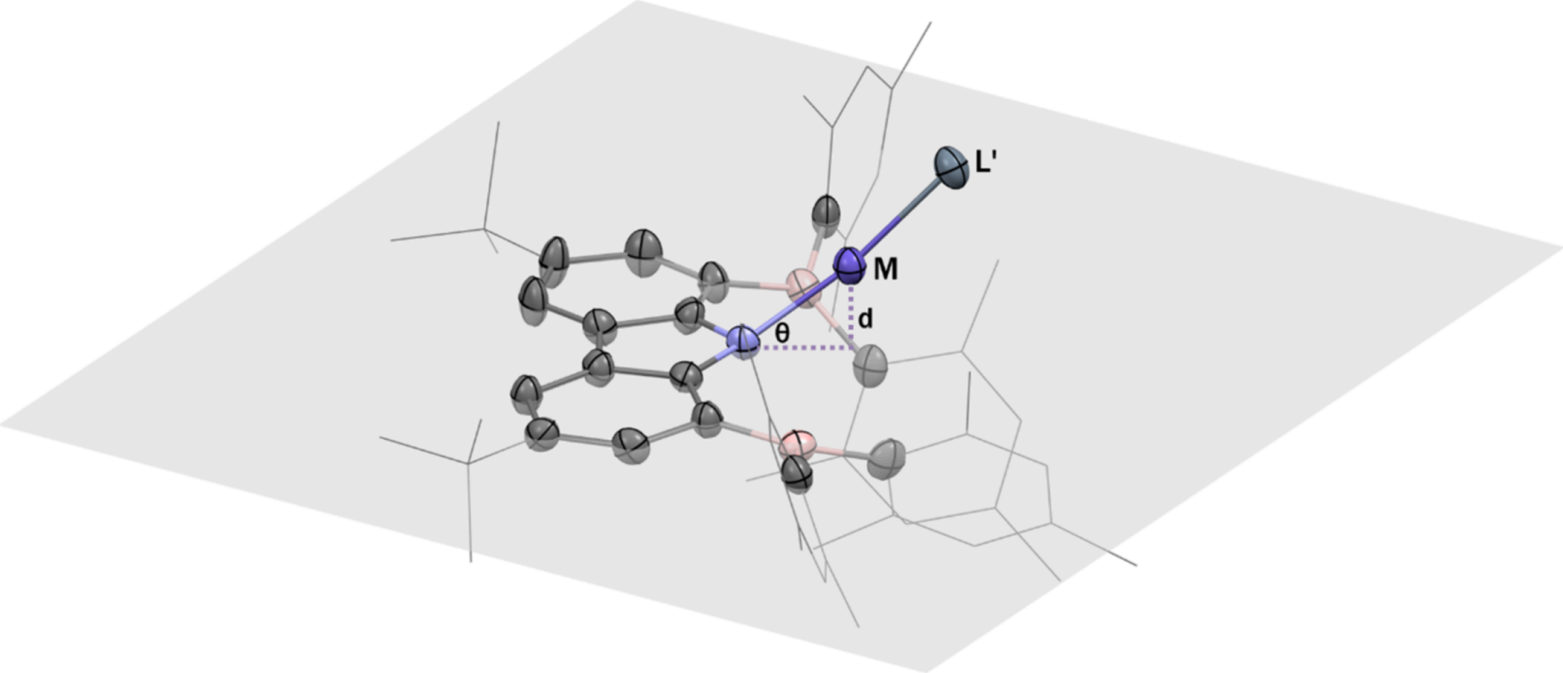
Selected Interatomic Distances and Angles for **1**–**4** and **6**–**10** ^a^

**complex**	**M–N**_**carbazole**_ **[Å]**	M**–**L′ (L′ = N/P/CI) [Å]	**N**_**carbazole**_ ****–**M**–**L** **′** **[°]**	**M**⋯**C** **[Å]**	***d* [Å]**	**θ [°]**
1 (Li)	1.900(9)			2.384(9)–2.538(9)	0.280	7.34
2 (Fe)	1.960(2)	2.2217(9)	119.37(7)	2.460(3), 2.651(3)	0.145	5.25
3 (Co)	1.901(3)	2.2039(13)	119.53(10)	2.477(4), 2.536(5)	0.115	5.45
4 (Zn)	1.892(3)	2.1291(11)	133.49(9)	2.436(4), 2.885(4)	0.280	8.69
6 (Ni)	1.914(3)			2.141(3)–2.458(3)	0.299	7.99
7 (Cu)	1.918(2)			2.159(3)–2.452(3)	0.093	3.53
8 (AgPPh_3_)	2.122(2)	2.3917(9)	157.73(7)	>3	1.130	28.21
9 (AuPPh_3_)	2.052(4)	2.2491(14)	171.38(11)	>3	1.309	34.55
10 (CuDMAP)	1.854(3)	1.881(3)	171.52(13)	>3	0.374	9.82

a The angle θ represents the deviation of the M–N vector from the carbazole plane (light gray), while *d* denotes the vertical distance from the metal center (M) to this mean plane.

The B···M distances in all cases exceed 3.0 Å, and the boron atoms maintain a trigonal planar geometry, suggesting the absence of significant metal–boron interactions. Notably, ligand BMes_2_CBz supports highly unusual low-coordinate halide complexes of first-row transition metals. To the best of our knowledge, complexes **2** and **3** represent the first crystallographically characterized Fe­(II) and Co­(II) halide species with a formally two-coordinate geometry, albeit with weak secondary interactions from the ligand periphery. For zinc, only a single structurally authenticated two-coordinate Zn­(II) halide complex has been reported, stabilized by an extremely bulky amido ligand, as described by Jones and Hicks.^41^ In contrast, monodentate bulky ligands such as terphenyls groups typically give rise to halide-bridged dimers or heterocubane-type clusters in Fe, Co, and Zn systems.^47–50^ Even the most sterically demanding carbazolyl and amido ligands reported previously favor distorted tetrahedral geometries or bridged architectures in Fe­(II) and Co­(II) complexes.^17,41^ In comparison, our results highlight the distinct steric and conformational profile of the diborylcarbazolyl ligand BMes_2_CBz, which effectively enforces low coordination numbers while preserving mononuclear structures.

In the case of chromium, the reaction of **1** with 7 equiv of CrCl_2_ led to the formation of a rare trimetallic complex, (**LCr**)**_2_** (**CrCl**_**4**_) (**5**), featuring a planar [CrCl_4_]^2–^ unit bridging two three-coordinate LCr fragments (Scheme 2). However, the similar solubility profiles of complex **5** and unreacted ligand **1** posed challenges in isolating **5** in an analytically pure form, limiting its full characterization. Although the crystal quality was suboptimal, the atomic connectivity is unambiguous, confirming the trinuclear architecture, as shown in the Supporting Information (Figure S40). Structurally related examples featuring a bridging planar [CrCl_4_]^2–^ unit coordinated to two chromium centers have been reported by Gao, Mu, and co-workers.^51^ In contrast, the reaction of **1** with MnCl_2_ yielded inseparable mixtures of multiple products and unreacted ligands, from which no isolable or identifiable manganese complex could be obtained.

We next turned our attention to later 3d transition metals (Ni and Cu) and coinage metals (Ag and Au). Treatment of **1** with an excess of NiCl_2_ (4.9 equiv) at 60 °C afforded a mixture containing pseudo-one-coordinate Ni­(I) complex **LNi** (**6**) as a deep purple solid in 41% isolated yield, along with **LH** and other uncharacterized paramagnetic species (Scheme 3). The effective magnetic moment of **6** (μ_eff_ = 2.3 μ_B_), as determined by the Evans method, is consistent with a d^9^, *S* = 1/2 configuration. Notably, use of only 1.1 equiv of NiCl_2_ led to the same outcome (Figure S29 in the Supporting Information), albeit with a longer reaction time. We propose that formation of **LNi** proceeds via initial generation of an LNi­(II)Cl intermediate, followed by single-electron transfer (SET) from **LLi** to LNi­(II)Cl to produce an aminyl radical (L·) and **LNi**(**I**). Subsequent hydrogen-atom capture by the aminyl radical accounts for the formation of **LH**, as observed by ^1^H NMR spectroscopy. Although aminyl radical is rare, several examples have been reported.^52–54^ Related Ni­(I) complexes formed via reduction of Ni­(II) precursors with organic alkali-metal reagents have also been described.^55,56^ Moreover, the enhanced instability and facile reducibility of two-coordinate Ni­(II) complexes relative to their Fe and Co congeners have likewise been observed in bis­(amido) systems.^57,58^ The isolation of **6** suggests the stability of BMes_2_CBz toward low-valent metal centers, likely aided by the steric protection provided by the flanking mesityl substituents. The reaction of **1** with CuCl (1.3 equiv) in THF similarly afforded **LCu** (**7**) as a dark red solid in 94% yield. Complex **7** is diamagnetic, and its ^11^B­{^1^H} NMR spectrum shows a broad resonance at 72.3 ppm, closely resembling those of complexes **1** and **4**. In addition, the reaction of **1** with CuCl_2_ yielded uncharacterized paramagnetic species. This part of the study remains under investigation and is not discussed further in the present work. Attempts to isolate the heavier analogues LAg and LAu using Ag­(I) and Au­(I) precursors, including AgOTf and Au­(THT)Cl (THT = tetrahydrothiophene), were unsuccessful. These species proved unstable under ambient conditions and decomposed into an insoluble solid, **LH**, and uncharacterized byproducts. These observations are suggestive of reduction processes potentially formation of the M(0) solid. In the case of gold, a transient intermediate assigned to LAu was observed by ^1^H NMR, but it ultimately decomposed to black solid and **LH** in C_6_D_6_ solution (Figures S30 and 31 in the Supporting Information). For silver, only gray solid, **LH**, and uncharacterized species were detected. The instability of LAg and LAu may be attributed to the hard–soft mismatch between the hard anionic carbazolide donor and the soft Ag­(I) and Au­(I) centers, combined with the limited coordination sphere of BMes_2_CBz, which may be inadequate to stabilize larger coinage metal ions (covalent radii: Cu: 1.32 Å; Ag: 1.45 Å; Au: 1.36 Å).^34^ Slow evaporation of Et_2_O solutions of **6** and **7** provided single crystals suitable for X-ray diffraction. The solid-state structures of both complexes reveal that they are nearly isostructural with compound **1**, all adopting a pseudo-one-coordinate geometry (Figure 2). The M–N_carbazole_ distances are 1.914(3) Å for Ni (**6**) and 1.918(2) Å for Cu (**7**), in line with previously reported metal carbazolide complexes.^59–61^ Additionally, secondary interactions between the metal centers and the π-system of the mesityl substituents are evident in both structures. Notably, the Ni···C­(Mes) distances in **6** are similar, albeit slightly longer, than those reported for Ni­(I) sandwich complexes from the Krossing group.^62,63^ By contrast, the Cu···C­(Mes) distances in **7** are closely comparable to those observed in terphenyl Cu­(I) complexes reported by Niemeyer and Kays et al.^64,65^ Key structural metrics for complexes **6** and **7** are summarized in Table 1, which highlights a consistent trend of slightly elongated bond lengths in the Cu complex relative to Ni, in agreement with the increasing covalent radii across the group. Though LAg and LAu could not be isolated due to intrinsic instability, these species could be stabilized through coordination with L-type ligands such as PPh_3_. This strategy parallels our previous work on coinage metal complexes featuring a di-iron hydrido scaffold, where phosphine adducts of Cu, Ag, and Au precursors were used to access heterometallic species.^66^ Accordingly, the reactions of **1** with Ag­(PPh_3_)­OTf and Au­(PPh_3_)Cl cleanly yielded **LMPPh**_**3**_ complexes [M = Ag (**8**) and Au (**9**)] as orange (**8**) and golden yellow (**9**) solids in high yields (Scheme 4). Complex **9** could also be obtained by adding PPh_3_ to a C_6_D_6_ solution containing the in situ-generated LAu intermediate. In contrast to the Ag and Au cases, attempts to synthesize the corresponding LCuPPh_3_ complex from either **LCu** (**7**) or CuPPh_3_Cl were unsuccessful; in both cases, **LCu** (**7**) was isolated with the recovery of free PPh_3_, indicating that steric hindrance prevents phosphine coordination in the Cu system. For Ag and Au, the larger covalent radii and the soft character of the metal centers allow successful coordination with PPh_3_ despite the steric bulk of BMes_2_CBz. Interestingly, although PPh_3_ could not coordinate to complex **7**, a smaller L-type ligand, DMAP (4-dimethylaminopyridine), did bind to afford **LCu**(**DMAP**) (**10**) as an orange-red solid in 99% yield. Complexes **8**–**10** exhibit similar NMR characteristics: the ^1^H NMR spectra are consistent with symmetry across the N_carbazole_–M axis, and the ^11^B NMR spectra show broad resonances at 74.9 (**8**), 74.7 (**9**), and 77.5 ppm (**10**), suggesting that the boron centers remain three-coordinate and noninteracting with the metal centers.

**Scheme 3:**
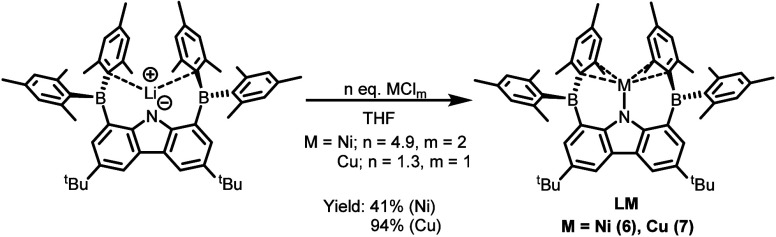
Synthesis of Complexes **6** and **7**

**Scheme 4:**
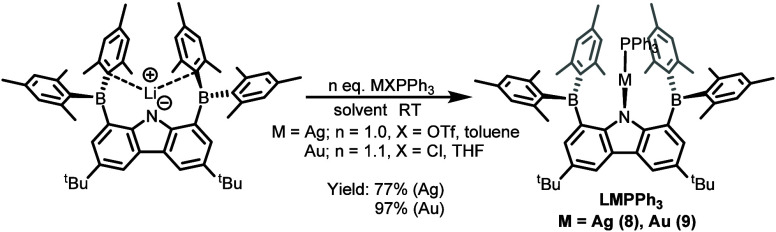
Synthesis of Complexes **8** and **9**

Single-crystal X-ray diffraction reveals that complexes **8**–**10** adopt two-coordinate linear geometries with N_carbazole_–M–L′ angles of 157.73(7)° (Ag, **8**), 171.38(11)° (Au, **9**), and 171.52(13)° (Cu, **10**) (see Figure 2). The deviation from linearity in the Ag complex is likely due to a nearby THF molecule in the crystal lattice, which perturbs the coordination environment and compresses the N_carbazole_–Ag–P angle. Distinct from complexes **1**–**7**, where the metal centers lie approximately within the carbazole plane to optimize σ-donation and π-bonding interactions, complexes **8**–**10** exhibit “lifted” metal centers, with displacements from the carbazole plane of 0.374 (Cu, **10**), 1.130 (Ag, **8**), and 1.309 Å (Au, **9**). The angles between the M–N bond vector and the carbazole plane also increase from 9.82° (Cu) to 28.21° (Ag) and 34.55° (Au), reflecting the increased steric repulsion from the ancillary ligands. This structural distortion is rationalized by considering the electronic and steric demands of the metal centers. The electron-deficient, low-coordinate LCu, LAg, and LAu fragments favor coordination by additional L-type ligands; however, linear two-coordinate geometries typical of d^10^ systems cannot be achieved within the carbazole plane due to steric clash with the flanking BMes_2_ groups. Consequently, the metal centers are displaced out of the plane to accommodate linear N–M–L′ angles.

Taken together, the low-coordinate metal complexes described in this study fall into three structural classes: pseudo-one-coordinate (**1**, **6**, and **7**), two-coordinate (**2**–**4** and **8**–**10**), and trinuclear (**5**). The pseudo-one-coordinate species feature metal centers embedded in the central cavity of the ligand and stabilized primarily by N_carbazole_ coordination and secondary arene interactions. As the metal covalent radius increases (e.g., Ag and Au), the cavity becomes less able to stabilize the metal alone, leading to instability, unless auxiliary ligands are present. The two-coordinate complexes adopt either bent (**2**–**4**) or linear (**8**–**10**) geometries depending on the nature of the metal and the coordinated L′ ligand. The degree of metal tilting out of the carbazole plane correlates with steric repulsion from the ancillary ligand and the size of the metal center. Lastly, the trinuclear Cr complex (**5**) likely forms due to a combination of electronic and steric factors, with the LCrCl fragment incorporating an additional CrCl_2_ unit rather than forming a halide-bridged dimer, due to repulsion between the bulky BMes_2_ groups.

### Steric Profile Quantified by %*V*_bur_

To quantify and visualize the steric influence of the ligand on the metal center, buried volume calculations (%*V*_bur_) and steric maps were performed (Figure 3).^67,68^ BMes_2_CBz was evaluated in comparison with other sterically demanding monodentate carbazolyl ligands commonly used in low-coordinate metal chemistry. Substituents at the 1,8-positions—including *tert*-butyl, phenyl, 3,5-di-*tert*-butylphenyl and anthracene—impart varying degrees of steric hindrance. The calculated %*V*_bur_ values for all bulky monodentate carbazolyl ligands are summarized in the Supporting Information (Table S12). Notably, the two BMes_2_ groups (%*V*_bur_ = 81.4) create a more effectively shielded cavity than anthracene substituents (%*V*_bur_ = 74.0), highlighting the importance of substituent orientation in modulating steric protection. In the pseudo-one-coordinate complexes, the %*V*_bur_ values all exceed 80.0, with slight variation depending on the metal center (Table S11). Additionally, %*V*_bur_ values of complexes **2**–**4** [74.8 (Fe), 75.1 (Co), and 66.1 (Zn)] are higher than those reported for bulky amide ligands [48.9–62.5, Ar*­(Ph_3_Si)­N], typical β-diketiminate (NacNac) ligands (57.3–66.3), carbazolyl ligand (44.4 and 45.3), and terphenyl ligands (43.7 and 45.9) in low-coordinate transition metal halide complexes (Table S13).

**Figure 3:**
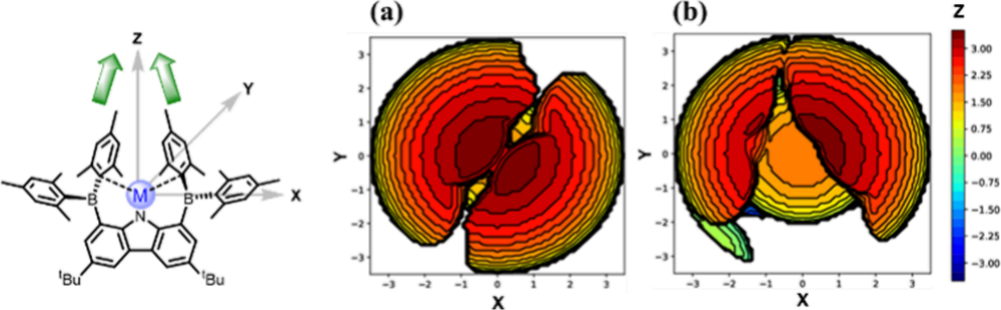
Steric map of complex (a) **1** and (b) **2**; metal atom was chosen as the origin; *Z*-axis was defined along the vector from the nitrogen atom of the carbazole ring to the metal center, with the *XZ*-plane corresponding to the plane of the carbazole ring, sphere with *r* = 3.5 Å.

### Computational Studies and EPR Measurement

To gain insight into the electronic structures of this series of complexes, we first analyzed the frontier molecular orbitals (MOs) of complex **1**. The HOMO of complex **1** is primarily composed of delocalized π-bonding over the carbazole framework. The energies of LUMO and LUMO+1 are nearly the same, and both display notable contributions from empty p-orbitals of the boron moieties, suggesting that these sites may serve as potential reactive centers (Figure 4a). Similar MO characteristics are observed for complexes **6** and **7** (Figures S53 and S54 in the Supporting Information), supporting the prospect of metal–ligand cooperativity, which will be explored in future studies. To complement the orbital analysis and directly probe the electronic structures of the low-coordinate species, continuous-wave (CW) X-band EPR spectroscopy was carried out. Complex **2** showed no signal at 77 K, consistent with an integer spin (*S* = 2) and the expected EPR silence at perpendicular mode (Figure S32 in the Supporting Information).

**Figure 4:**
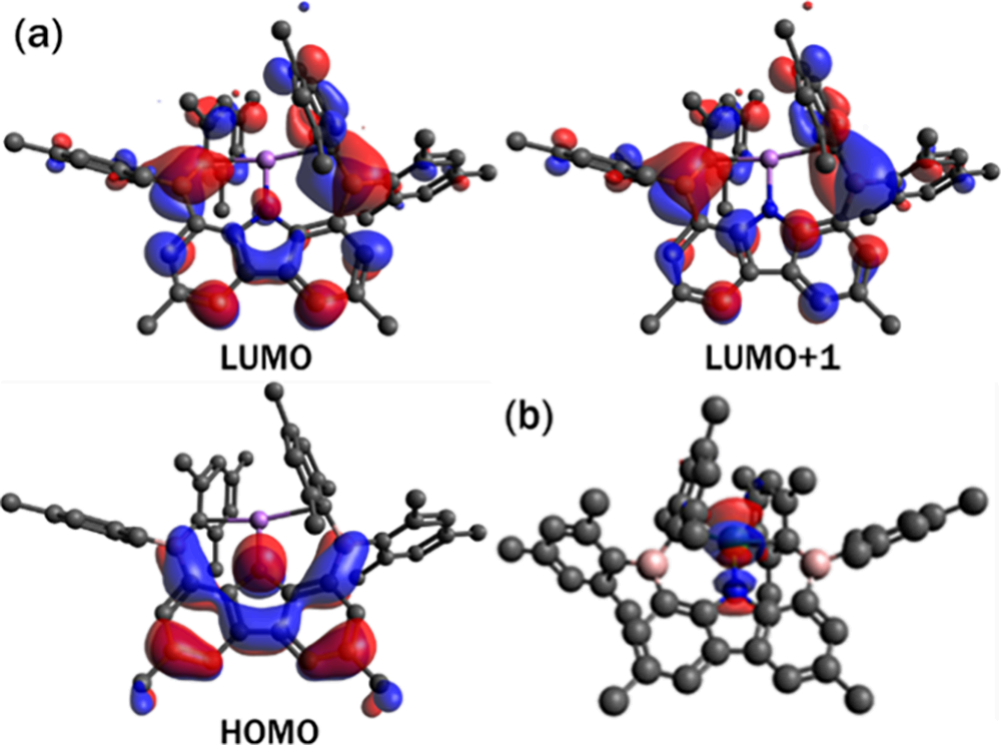
(a) MO plot of the HOMO, LUMO, and LUMO+1 of complex **1** with an isosurface value of 0.025; (b) unrestricted natural spin orbital of complex **6**.

In contrast, complex **6** displayed broader features extending across from 250 to 350 mT at 77 K (see Figure 5). The best fit gave *g*-values of 1.98, 2.25, and 2.50, indicating a pronounced rhombic anisotropy. Hyperfine couplings were assigned to ^14^N (28.3, 0, 29.4 MHz). Significant strain terms (*H*_strain_ = [47.3, 123, 191] MHz; *g*_Strain_ = [0, 0.0157, 0.0415]) were needed to reproduce the breadth of the features, alongside a narrow intrinsic line width (0.2 mT). The strong anisotropy (Δ*g* ≈ 0.52) reflects spin–orbit coupling contributions from $d_{x^{2}‐y^{2}}$ and $d_{z^{2}}$ orbitals in the Ni­(I) environment. Complex **6** exhibits highly anisotropic *g*-tensors, characteristic of first-row late-transition-metal *S* = 1/2 species, and displays broad, strain-dominated spectra that require inclusion of both Ni and N hyperfine couplings. To complement the experimental fitting, density functional theory (DFT) calculations were performed (Table S14 in the Supporting Information). For **6**, the experimental spectrum is dominated by rhombic *g*-anisotropy (1.98, 2.25, and 2.50) with N hyperfine couplings. Among the functionals tested, the range-separated hybrid GGA functional wB97X gave the closest agreement with experiment, particularly in reproducing the large *g*-shift along *g*_*z*_. It predicts *g* = 2.02, 2.23, and 2.35 and |A­(N)| = 25, 19, and 18 MHz, in line with the enhanced *g*_*z*_ and moderate hyperfine anisotropy observed experimentally. Unrestricted natural spin orbital (UNSO) analyses (Figure 4b) confirm that for the unpaired electron in complex **6**, the spin density is distributed between Ni $d_{x^{2}‐y^{2}}$ and $d_{z^{2}}$ orbitals with appreciable delocalization onto the carbazole nitrogen atom, in agreement with the modest but non-negligible |A­(N)| couplings. The percentages of unpaired electrons in the $d_{x^{2}‐y^{2}}$ and $d_{z^{2}}$ orbitals are obtainable from Mulliken spin population analysis and determined to be 49.7% and 31.3%, respectively.

**Figure 5:**
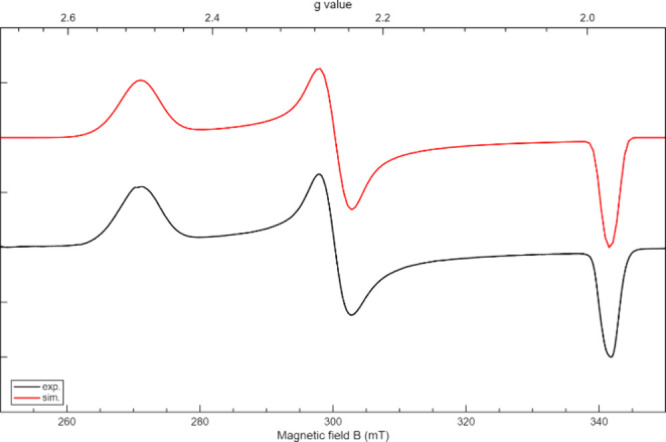
Experimental (black) and simulated (red) EPR spectra of complex **6**. Fit parameters: *g*_1_ = 1.9805 ± 0.0001, *g*_2_ = 2.2528 ± 0.0001, and *g*_3_ = 2.5006 ± 0.0002; *A*_1_ = 28.3 ± 0.4 MHz, *A*_2_ = 0 MHz, and *A*_3_ = 29.4 ± 3.2 MHz (^14^N); *g*_strain_ = [0, 0.0157, 0.0415] ± [0, 0.0008, 0.06]. *A*_strain_ = [0, 0, 75.6] ± [0, 0, 100] MHz; *H*_strain_ = [47.3, 123, 191] ± [2.2, 0, 160] MHz; lw_pp_ = [0.0909, 0.240] ± [0, 0.026] mT.

## Conclusions

In summary, we have synthesized a new carbazolyl ligand, L, bearing two bulky and Lewis acidic BMes_2_ substituents. The corresponding **LLi** (**1**) serves as an effective precursor to a range of low-coordinate transition metal complexes spanning three structural motifs: pseudo-one-coordinate, two-coordinate, and trinuclear. Diamagnetic species were characterized by NMR spectroscopy, while paramagnetic species were investigated by EPR and magnetic susceptibility measurements. Single-crystal X-ray diffraction provided detailed structural information, confirming that the boron centers retain a trigonal planar geometry and do not engage in M→B interactions. Theoretical calculation indicates that boron moieties are the highly potential reactive sites. These findings demonstrate that the diborylcarbazolyl framework, BMes_2_CBz, combines steric and electronic features conducive to the stabilization of low-coordinate metal species and holds promise for future applications in metal–ligand cooperative reactivity.

## Experimental Section

### General Information

All manipulations with oxygen- and moisture-sensitive materials were performed in a nitrogen-filled glovebox or by using standard Schlenk line techniques. All glassware was dried in an oven at 150 °C for at least 3 h. Solvents were dried and deaerated using a solvent system (AsiaWong Enterprise Co., Ltd.) prior to use and stored under N_2_ over 3 Å molecular sieves. Benzene-*d*_6_ was dried over sodium and benzophenone, degassed by three freeze–pump–thaw cycles, and stored in the glovebox. The NMR spectra were recorded using a Varian 400 MHz, Varian 600 MHz, or JEOL 400 MHz spectrometer. The NMR spectra were referenced to residual proteosolvent for ^1^H NMR (7.16 ppm for compound in benzene-*d*_6_), to deuterated solvent for ^13^C NMR (128.06 ppm for compound in benzene-*d*_6_). All NMR spectra were recorded at 25 °C. Complex multiplets are noted as “m” and broad resonances as “br”. The EPR spectra were recorded using a Bruker ELEXSYS E-580 spectrometer. The UV spectra were recorded at 25 °C using a Hitachi U-2900 spectrophotometer. Elemental analyses were performed using an Elementar Vario EL CUBE (CHN-OS Rapid, Germany). CrCl_2_, FeCl_2_, CoCl_2_, NiCl_2_, CuCl, DMAP, and *n*-BuLi were commercially procured and used as received. BF­(Mes)_2_,^69^ 1,8-dibromo-3,6-di-*tert*-butylcarbazole,^28^ ZnCl_2_(THF),^70^ Cu­(PPh_3_)­Cl,^71^ Ag­(PPh_3_)­(OTf),^72^ Au­(PPh_3_)­Cl,^73^ and Au­(THT)­Cl^74^ were synthesized according to the reported procedures.

### Safety Notification

Great attention must be exercised when working with pyrophoric chemicals like *n*-BuLi. The experiments were carried out using these substances under inert conditions, employing the Schlenk technique or gloveboxes.

### Synthesis of Compounds

#### BrCBzLi

A solution of *n*-BuLi (19.0 mL, 47.5 mmol, 2.5 M in hexane) was added dropwise to a 300 mL pentane solution of 1,8-dibromo-3,6-di-*tert*-butylcarbazole (20.983 g, 47.9 mmol). The reaction mixture was stirred at room temperature for 2 h. The white solids were removed by filtration through a G4 frit. The white solids were further crystallized with THF (75 mL). Yield: 18.530 g (66%).

^1^H NMR (C_6_D_6_, 400 MHz, 298 K): δ (ppm) = 1.20–1.23 (m, 8H, THF-β-CH_2_), 1.47 (s, 18H, C­(CH_3_)_3_), 3.47–3.50 (m, 8H, THF-α-CH_2_), 7.97 (d, 2H, *J* = 1.7 Hz, Carb-CH), 8.46 (d, 2H, *J* = 1.7 Hz, Carb-CH).

#### **LLi** (**1**)

A solution of *n*-BuLi (55.0 mL, 137.5 mmol, 2.5 M in hexane) was added dropwise to a 150 mL diethyl ether solution of **BrCBzLi** (20.009 g, 34.1 mmol). The reaction mixture was stirred at room temperature for 3 h. Subsequently, a 30 mL diethyl ether solution of BF­(Mes)_2_ (19.157 g, 71.4 mmol) was added, and the reaction mixture was stirred for 14 h. The red solids were removed by filtration through a G4 frit. The solids were extracted with toluene (3 × 75 mL), and the combined toluene extracts were dried under reduced pressure to afford **LLi** (**1**) as a red solid. Yield: 11.995 g (45%). Red single crystals suitable for X-ray diffraction and analytically pure samples suitable for elemental analysis were obtained by slow evaporation from diethyl ether.

^1^H NMR (C_6_D_6_, 400 MHz, 298 K): δ (ppm) = 1.45 (s, 18H, C­(CH_3_)_3_), 1.96 (s, 6H, Mes-*p*-CH_3_), 2.00 (s, 12H, Mes-*o*-CH_3_), 2.16 (s, 12H, Mes-*o*-CH_3_), 2.22 (s, 6H, Mes-*p*-CH_3_), 6.45 (s, 4H, Mes-CH), 6.87 (s, 4H, Mes-CH), 8.04 (d, 2H, *J* = 2.0 Hz, Carb-CH), 8.72 (d, 2H, *J* = 2.0 Hz, Carb-CH); ^13^C­{^1^H} NMR (C_6_D_6_, 100 MHz, 298 K): δ (ppm) = 21.4, 21.4, 22.7, 23.9, 32.4, 34.6, 123.5, 126.9, 128.6, 129.9 130.3, 134.9, 137.6, 138.0, 140.0, 141.0, 141.4, 141.8, 144.2, 157.1; ^11^B­{^1^H} NMR (C_6_H_6_, 128 MHz, 298 K): δ (ppm) = 71.3 (Δν_1/2_ (fwhm) = 3000 Hz); ^7^Li­{^1^H} NMR (C_6_D_6_, 155 MHz, 298 K): δ (ppm) = 0.5.

UV–vis: λ (ε) = 290 nm (24000 cm^–1^ M^–1^), 387 nm (8200 cm^–1^ M^–1^), 492 nm (13000 cm^–1^ M^–1^) in THF.

Elem. anal. calc. for C_56_H_66_B_2_LiN: C 86.04%, H 8.51%, N 1.79%; Found: C 85.92%, H 8.69%, N 1.90%.

#### **LH** (**1-H**)

A 10 mL toluene solution of **LLi** (312 mg, 0.40 mmol) was exposed to air, resulting in an immediate color change to green. The reaction mixture was stirred under air at room temperature for 3 h, after which the volatiles were removed under reduced pressure. The residue was extracted with toluene (3 × 10 mL), and the combined extracts were concentrated under reduced pressure to afford **LH** (**1**-**H**) as a green solid. Yield: 307 mg (99%). Green single crystals suitable for X-ray diffraction and analytically pure samples suitable for elemental analysis were obtained by slow evaporation from diethyl ether.

^1^H NMR (C_6_D_6_, 400 MHz, 298 K): δ (ppm) = 1.33 (s, 18H, C­(CH_3_)_3_), 2.03 (s, 24H, Mes-*o*-CH_3_), 2.17 (s, 12H, Mes-*p*-CH_3_), 6.72 (s, 8H, Mes-CH), 7.85 (d, 2H, *J* = 2.0 Hz, Carb-CH), 8.46 (d, 2H, *J* = 1.8 Hz, Carb-CH), 8.50 (s, 1H, NH); ^13^C­{^1^H} NMR (C_6_D_6_, 100 MHz, 298 K): δ (ppm) = 21.5, 23.2, 32.1, 32.3, 34.8, 121.3, 122.8, 129.1, 129.5, 132.6, 139.0, 141.2, 142.6, 142.9; ^11^B­{^1^H} NMR (C_6_H_6_, 128 MHz, 298 K): δ (ppm) = 69.5 (Δν_1/2_ = 3400 Hz).

UV–vis: λ (ε) = 295 nm (14000 cm^–1^ M^–1^), 332 nm (22000 cm^–1^ M^–1^), 392 nm (14000 cm^–1^ M^–1^) in THF.

Elem. anal. calc. for C_56_H_67_B_2_N: C 86.70%, H 8.71%, N 1.81%; Found: C 86.86%, H 8.62%, N 1.67%.

#### **LFeCl** (**2**)

A 10 mL THF solution of **LLi** (188 mg, 0.24 mmol) was added to a suspension of FeCl_2_ (39 mg, 0.31 mmol) in 5 mL of THF, resulting in an immediate color change to reddish-brown. The reaction mixture was stirred at room temperature for 12 h, after which the volatiles were removed under reduced pressure. The residue was extracted with toluene (3 × 10 mL), and the combined extracts were concentrated under reduced pressure to afford **LFeCl** (**2**) as a reddish-brown solid. Yield: 198 mg (95%). Brown single crystals suitable for X-ray diffraction were obtained by slow evaporation from the diethyl ether. Analytically pure samples suitable for elemental analysis were obtained by slow evaporation from a diethyl ether/dichloromethane mixed solvent system.

Magnetic susceptibility: μ_eff_ = 5.5 μ_B_ (Evans method, C_6_D_6_, 298 K; capillary reference: 2% v/v C_6_H_6_ in C_6_D_6_).

UV–vis: λ (ε) = 315 nm (15000 cm^–1^ M^–1^), 388 nm (9400 cm^–1^ M^–1^), 480 nm (6200 cm^–1^ M^–1^) in THF.

Elem. anal. calc. for C_56_H_66_B_2_ClFeN·(C_4_H_10_O)·(CH_2_Cl_2_): C 71.47%, H 7.67%, N 1.37%; Found: C 71.48%, H 7.74%, N 1.46%.

#### **LCoCl** (**3**)

A 10 mL THF solution of **LLi** (206 mg, 0.26 mmol) was added to a suspension of CoCl_2_ (41 mg, 0.32 mmol) in 5 mL of THF, resulting in an immediate color change to brown. The reaction mixture was stirred at room temperature for 12 h, after which the volatiles were removed under reduced pressure. The residue was extracted with toluene (3 × 10 mL), and the combined extracts were concentrated under reduced pressure to afford **LCoCl** (**3**) as a yellow-brown solid. Yield: 226 mg (99%). Brown single crystals suitable for X-ray diffraction and analytically pure samples suitable for elemental analysis were obtained by slow evaporation from diethyl ether.

Magnetic susceptibility: μ_eff_ = 4.5 μ_B_ (Evans method, C_6_D_6_, 298 K; capillary reference: 2% v/v C_6_H_6_ in C_6_D_6_).

UV–vis: λ (ε) = 410 nm (8500 cm^–1^ M^–1^), 445 nm (7300 cm^–1^ M^–1^) in THF.

Elem. anal. calc. for C_56_H_66_B_2_ClCoN·2­(C_4_H_10_O): C 75.56%, H 8.52%, N 1.38%; Found: C 75.90%, H 8.26%, N 1.52%.

#### **LZnCl** (**4**)

A 10 mL THF solution of **LLi** (103 mg, 0.13 mmol) was added to a 5 mL THF solution of ZnCl_2_(THF) (30 mg, 0.14 mmol), resulting in an immediate color change to orange-red. The reaction mixture was stirred at room temperature for 12 h, after which the volatiles were removed under reduced pressure. The residue was extracted with toluene (3 × 10 mL), and the combined extracts were concentrated under reduced pressure to afford **LZnCl** (**4**) as an orange-brown solid. Yield: 108 mg (94%). Orange single crystals suitable for X-ray diffraction were obtained by slow evaporation from diethyl ether. Analytically pure samples suitable for elemental analysis were obtained by slow evaporation from a diethyl ether/dichloromethane mixed solvent system.

^1^H NMR (C_6_D_6_, 400 MHz, 298 K): δ (ppm) = 1.36 (s, 18H, C­(CH_3_)_3_), 2.03 (s, 12H, Mes-*p*-CH_3_), 2.18 (s, 24H, Mes-*o*-CH_3_), 6.57 (br, 4H, Mes-CH), 6.79 (s, 4H, Mes-CH), 8.02 (d, 2H, *J* = 2.1 Hz, Carb-CH), 8.61 (d, 2H, *J* = 2.2 Hz, Carb-CH); ^13^C­{^1^H} NMR (C_6_D_6_, 100 MHz, 298 K): δ (ppm) = 21.2, 21.3, 21.4, 21.5, 25.2, 32.1, 34.7, 123.0, 125.6, 125.7, 125.8, 129.3, 129.9, 135.8, 136.1, 138.3, 140.8, 143.9, 144.5, 152.4; ^11^B­{^1^H} NMR (C_6_D_6_, 128 MHz, 298 K): δ (ppm) = 71.6 (Δν_1/2_ = 3600 Hz).

UV–vis: λ (ε) = 307 nm (15000 cm^–1^ M^–1^), 403 nm (8800 cm^–1^ M^–1^), 460 nm (4700 cm^–1^ M^–1^) in THF.

Elem. anal. calc. for C_56_H_66_B_2_ClZnN·(C_4_H_10_O)·(CH_2_Cl_2_): C 70.81%, H 7.60%, N 1.35%; Found: C 70.57%, H 7.36%, N 1.31%.

#### (**LCr**)_2_(**CrCl**_4_) (**5**)

A 5 mL THF solution of **LLi** (65 mg, 0.083 mmol) was added to a suspension of CrCl_2_ (72 mg, 0.58 mmol) in 10 mL of THF. The reaction mixture was stirred at room temperature for 3 days, after which the volatiles were removed under reduced pressure. The residue was extracted with toluene (3 × 10 mL), and the combined extracts were concentrated under reduced pressure to afford a dark red solid. Crude yield: 61 mg (80%). Single crystals suitable for X-ray diffraction were obtained by slow evaporation from diethyl ether.

Elem. anal. calc. No analysis given due to the difficulties in isolating pure product.

#### **LNi** (**6**)

A 10 mL THF solution of **LLi** (235 mg, 0.30 mmol) was added to a suspension of NiCl_2_ (192 mg, 1.48 mmol) in 20 mL of THF. The reaction mixture was heated to 60 °C and stirred for 13 h, after which the volatiles were removed under reduced pressure. The residue was extracted with toluene (3 × 15 mL), and the combined extracts were concentrated under reduced pressure to afford the crude product, which was subsequently washed with pentane (3 × 15 mL) to afford **LNi** (**6**) as a deep purple solid. Yield: 102 mg (41%). Purple single crystals suitable for X-ray diffraction were obtained by slow evaporation from diethyl ether. Analytically pure samples suitable for elemental analysis were obtained by slow evaporation from a benzene/dichloromethane mixed solvent system.

Magnetic susceptibility: μ_eff_ = 2.3 μ_B_ (Evans method, C_6_D_6_, 298 K; capillary reference: 2% v/v C_6_H_6_ in C_6_D_6_).

UV–vis: λ (ε) = 315 nm (13000 cm^–1^ M^–1^), 374 nm (8400 cm^–1^ M^–1^), 515 nm (8900 cm^–1^ M^–1^) in THF.

Elem. anal. calc. for C_56_H_66_B_2_NiN·(C_6_H_6_)·(CH_2_Cl_2_): C 75.93%, H 7.49%, N 1.41%; Found: C 75.63%, H 7.84%, N 1.75%.

#### **LCu** (**7**)

A 10 mL THF solution of **LLi** (146 mg, 0.19 mmol) was added to a suspension of CuCl (25 mg, 0.25 mmol) in 5 mL of THF, resulting in an immediate color change to dark red. The reaction mixture was stirred at room temperature for 12 h, after which the volatiles were removed under reduced pressure. The residue was extracted with toluene (3 × 10 mL), and the combined extracts were concentrated under reduced pressure to afford **LCu** (**7**) as a dark red solid. Yield: 147 mg (94%). Red single crystals suitable for X-ray diffraction were obtained by slow evaporation from diethyl ether. Analytically pure samples suitable for elemental analysis were obtained by slow evaporation from a diethyl ether/THF mixed solvent system.

^1^H NMR (C_6_D_6_, 400 MHz, 298 K): δ (ppm) = 1.45 (s, 18H, C­(CH_3_)_3_), 1.93 (s, 24H, Mes-*o*-CH_3_), 1.98 (s, 6H, Mes-*p*-CH_3_), 2.15 (s, 6H, Mes-*p*-CH_3_), 6.37 (s, 4H, Mes-CH), 6.75 (s, 4H, Mes-CH), 7.92 (d, 2H, *J* = 2.1 Hz, Carb-CH), 8.71 (d, 2H, *J* = 2.1 Hz, Carb-CH); ^13^C­{^1^H} NMR (C_6_D_6_, 100 MHz, 298 K): δ (ppm) = 21.3, 21.4, 23.4, 23.5, 32.5, 34.8, 124.0, 125.0, 125.3, 128.4, 129.1, 130.9, 133.1, 134.9, 137.7, 138.2, 138.8, 141.0, 143.4, 154.5; ^11^B­{^1^H} NMR (C_6_H_6_, 128 MHz, 298 K): δ (ppm) = 72.3 (Δν_1/2_ = 3000 Hz).

UV–vis: λ (ε) = 294 nm (19000 cm^–1^ M^–1^), 325 nm (15000 cm^–1^ M^–1^), 397 nm (7500 cm^–1^ M^–1^), 502 nm (6600 cm^–1^ M^–1^) in THF.

Elem. anal. calc. for C_56_H_66_B_2_CuN·2­(C_4_H_8_O): C 78.24%, H 8.41%, N 1.43%; Found: C 78.11%, H 8.12%, N 1.52%.

#### **LAgPPh_3_** (**8**)

A 10 mL toluene solution of **LLi** (75 mg, 0.095 mmol) was added to a 5 mL toluene solution of AgOTfPPh_3_ (51 mg, 0.098 mmol). The reaction mixture was stirred at room temperature and avoiding light for 14 h, after which the volatiles were removed under reduced pressure. The residue was extracted with toluene (3 × 10 mL), and the combined extracts were concentrated under reduced pressure to afford **LAgPPh**_**3**_ (**8**) as an orange solid. Yield: 85 mg (77%). Orange single crystals suitable for X-ray diffraction and analytically pure samples suitable for elemental analysis were obtained by crystallization from a THF/pentane mixed solvent system at −35 °C.

^1^H NMR (C_6_D_6_, 400 MHz, 298 K): δ (ppm) = 1.46 (s, 18H, C­(CH_3_)_3_), 1.98 (s, 24H, Mes-*o*-CH_3_), 2.12 (s, 12H, Mes-*p*-CH_3_), 6.62 (br, 8H, Mes-CH), 6.90–7.07 (m, 9H, PPh_3_), 7.11–7.15 (m, 6H, PPh_3_), 7.93 (d, 2H, *J* = 1.8 Hz, Carb-CH), 8.70 (d, 2H, *J* = 2.1 Hz, Carb-CH); ^13^C­{^1^H} NMR (C_6_D_6_, 100 MHz, 298 K): δ (ppm) = 21.4, 32.5, 34.7, 122.2, 125.6, 125.7, 125.7, 128.6, 128.9, 129.0, 129.1, 129.3, 130.7, 133.3, 134.5, 134.7, 137.9, 138.3; ^11^B­{^1^H} NMR (C_6_D_6_, 128 MHz, 298 K): δ (ppm) = 74.9 (Δν_1/2_ = 4300 Hz).; ^31^P­{^1^H} NMR (C_6_D_6_, 162 MHz, 298 K): δ (ppm) = 11.3 (Δν_1/2_ = 1500 Hz).

UV–vis: λ (ε) = 387 nm (5700 cm^–1^ M^–1^), 503 nm (7700 cm^–1^ M^–1^) in THF.

Elem. anal. calc. for C_74_H_81_B_2_AgNP: C 77.63%, H 7.13%, N 1.22%; Found: C 77.58%, H 7.37%, N 1.00%.

#### **LAuPPh**_**3**_ (**9**)

A 5 mL THF solution of **LLi** (64 mg, 0.082 mmol) was added to a 5 mL THF solution of AuClPPh_3_ (43 mg, 0.087 mmol). The reaction mixture was stirred at room temperature for 13 h, after which the volatiles were removed under reduced pressure. The residue was extracted with toluene (3 × 10 mL), and the combined extracts were concentrated under reduced pressure to afford **LAuPPh**_**3**_ (**9**) as a golden yellow solid. Yield: 98 mg (97%). Yellow single crystals suitable for X-ray diffraction and analytically pure samples suitable for elemental analysis were obtained by crystallization from a THF/pentane mixed solvent system at −35 °C.

^1^H NMR (C_6_D_6_, 400 MHz, 298 K): δ (ppm) = 1.41 (s, 18H, C­(CH_3_)_3_), 1.86 (br, 24H, Mes-*o*-CH_3_), 2.27 (br, 12H, Mes-*p*-CH_3_), 6.64 (br, 8H, Mes-CH), 6.92–6.96 (m, 6H, PPh_3_), 7.00–7.07 (m, 9H, PPh_3_), 8.00 (d, 2H, *J* = 2.2 Hz, Carb-CH), 8.65 (d, 2H, *J* = 2.2 Hz, Carb-CH); ^13^C­{^1^H} NMR (C_6_D_6_, 100 MHz, 298 K): δ (ppm) = 32.2, 34.7, 120.3, 125.9, 126.0, 128.8, 128.9, 129.1, 129.3, 129.4, 130.0, 131.2, 131.2, 131.4, 133.8, 134.2, 134.4, 135.0, 135.1, 135.3, 137.5, 140.3, 142.0, 155.8; ^11^B­{^1^H} NMR (C_6_D_6_, 128 MHz, 298 K): δ (ppm) = 74.7 (Δν_1/2_ = 4800 Hz).; ^31^P­{^1^H} NMR (C_6_D_6_, 162 MHz, 298 K): δ (ppm) = 28.8.

UV–vis: λ (ε) = 325 nm (14000 cm^–1^ M^–1^), 380 nm (5200 cm^–1^ M^–1^), 410 nm (5100 cm^–1^ M^–1^), 447 nm (5100 cm^–1^ M^–1^) in THF.

Elem. anal. calc. for C_74_H_81_B_2_AuNP·(C_5_H_12_): C 72.64%, H 7.18%, N 1.07%; Found: C 72.30%, H 7.39%, N 1.03%.

#### **LCu**(**DMAP**) (**10**)

A 5 mL toluene solution of **LCu** (59 mg, 0.070 mmol) was added to a 5 mL toluene solution of DMAP (10 mg, 0.082 mmol), resulting in an immediate color change to orange-red. The reaction mixture was stirred at room temperature for 1 h, after which the volatiles were removed under reduced pressure to afford **LCu**(**DMAP**) (**10**) as an orange-red solid. Yield: 67 mg (99%). Orange single crystals suitable for X-ray diffraction were obtained by slow evaporation from diethyl ether.

^1^H NMR (C_6_D_6_, 400 MHz, 298 K): δ (ppm) = 1.46 (s, 18H, C­(CH_3_)_3_), 2.02 (s, 24H, Mes-*o*-CH_3_), 2.11 (s, 6H, DMAP-CH_3_), 2.19 (s, 12H, Mes-*p*-CH_3_), 5.73 (br, 4H, DMAP-CH), 6.68 (br, 8H, Mes-CH), 7.87 (d, 2H, *J* = 2.1 Hz, Carb-CH), 8.65 (d, 2H, *J* = 2.2 Hz, Carb-CH); ^13^C­{^1^H} NMR (C_6_D_6_, 100 MHz, 298 K): δ (ppm) = 21.4, 21.5, 32.5, 34.7, 38.2, 106.1, 120.9, 125.2, 125.7, 128.6, 129.3, 132.3, 134.7, 137.9, 138.5, 150.1, 154.2, 155.1; ^11^B­{^1^H} NMR (C_6_D_6_, 128 MHz, 298 K): δ (ppm) = 77.5 (Δν_1/2_ = 4400 Hz).

UV–vis: λ (ε) = 378 nm (8100 cm^–1^ M^–1^), 487 nm (12000 cm^–1^ M^–1^) in THF.

Elem. anal. calc. Despite several attempts, satisfactory elemental analysis results could not be obtained.

## Supplementary Material



## Notes

Compound characterizations, steric map, theoretical calculation details, and crystallographic data for all compounds (PDF)

## #

Department of Chemistry, Chung Yuan Christian University, Taoyuan City 320314, Taiwan
