# Supplementary material for: A Diborylcarbazolyl Ligand for Stabilizing Low-Coordinate and Low-Valent Metal Complexes
Source: Inorg Chem. 2026 Feb 23;65(9):4997–5008. doi: 10.1021/acs.inorgchem.5c05146 (PMC12977055; doi:10.1021/acs.inorgchem.5c05146)
Supplement: Supplementary file 1 [file ic5c05146_si_001.pdf]

Supporting Information

A Diborylcarbazolyl Ligand for Stabilizing Low-Coordinate and Low-Valent Metal Complexes

Yen-Hua Lee,[a] Wei-Chieh Chang,[a] Hsuan-Wen Fu,[a] Han-Jung Li,[a] Ting-Shen Kuo,[b] Tzuhsiung Yang,*[c] and Hsueh-Ju Liu*[a], [d]

[a] Department of Applied Chemistry, National Yang Ming Chiao Tung University, 1001 Daxue Rd, East District, Hsinchu 300093, Taiwan

[b] Department of Chemistry, National Taiwan Normal University, Taipei 116059, Taiwan

[c] Department of Chemistry, National Tsing Hua University, Hsinchu 300044, Taiwan

[d] Center for Emergent Functional Matter Science, National Yang Ming Chiao Tung University, 1001 Daxue Rd, East District, Hsinchu 300093, Taiwan

Corresponding author's E-mail: hsuehjuliu@nycu.edu.tw, yangzixiong@sais.org.cn

Table of content:

1. **NMR CHARACTERIZATIONS**
2. **EPR CHARACTERIZATIONS**
3. **SINGLE-CRYSTAL X-RAY DIFFRACTION CRYSTAL STRUCTURE FIGURES AND DATA TABLES**
4. **BURIED VOLUME ANALYSIS AND STERIC MAPS**
5. **COMPUTATIONAL RESULTS**
6. **REFERENCES**

1. NMR Characterizations

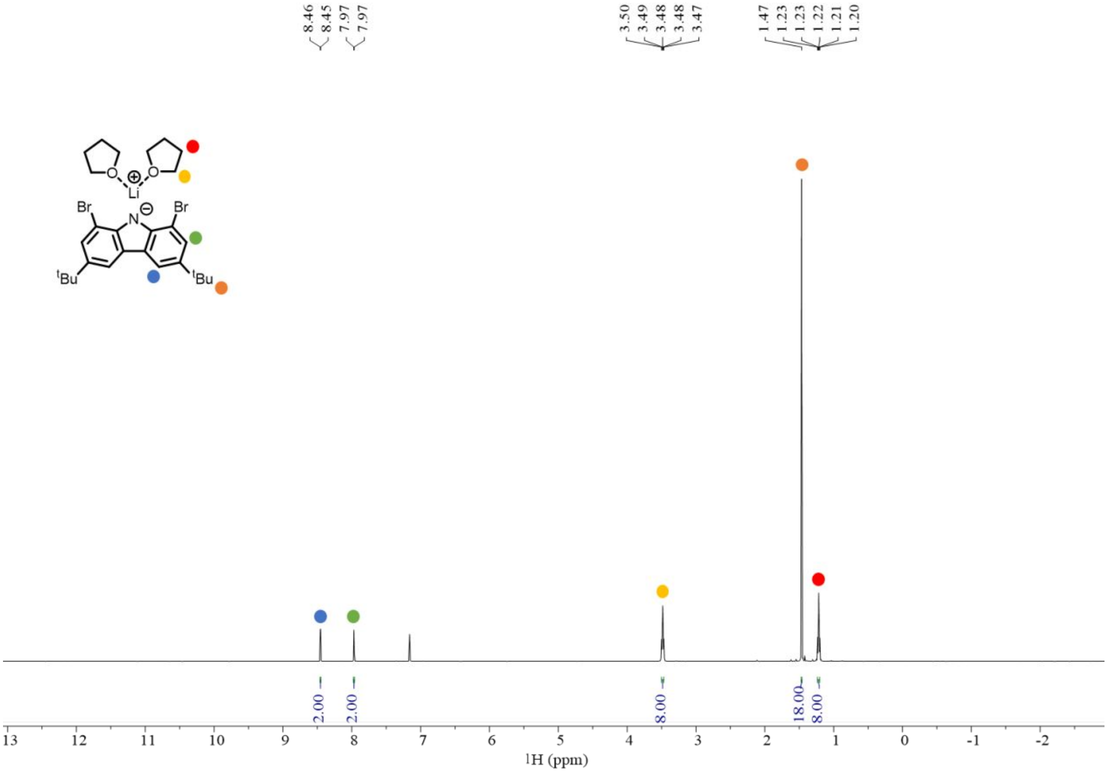

Figure S1. ^{1}H NMR spectrum of **BrCBzLi** in C_6D_6.

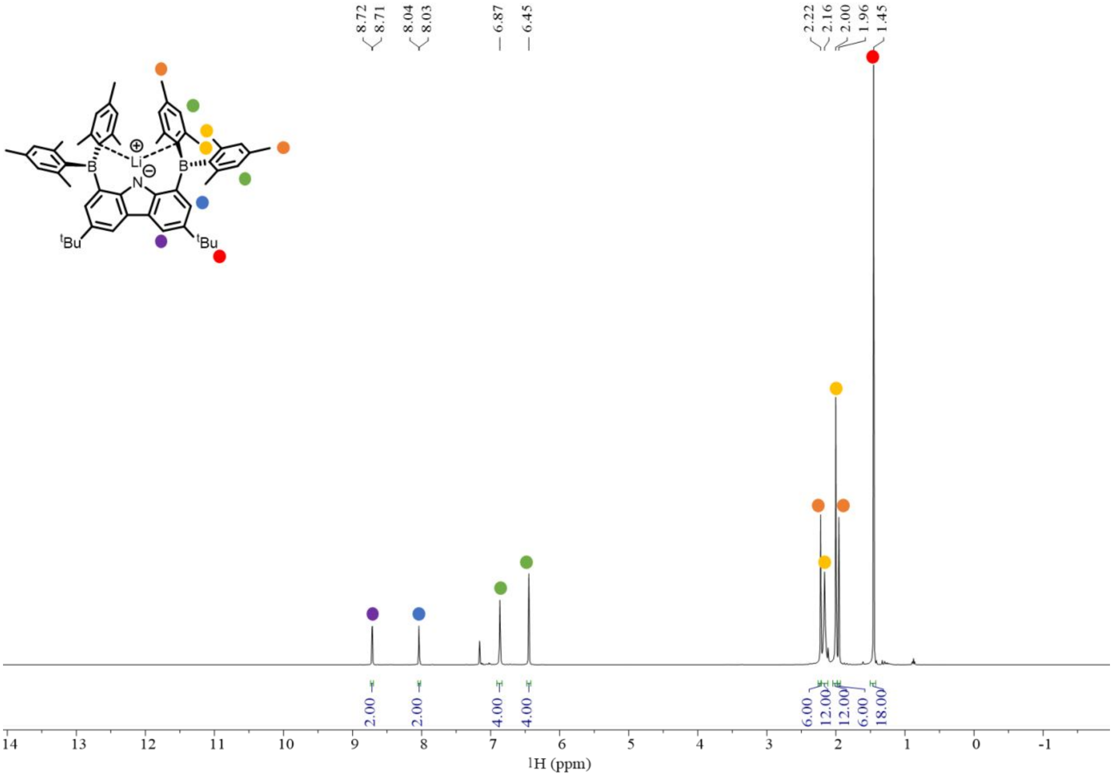

Figure S2. ^{1}H NMR spectrum of **LLi** (**1**) in C_6D_6.

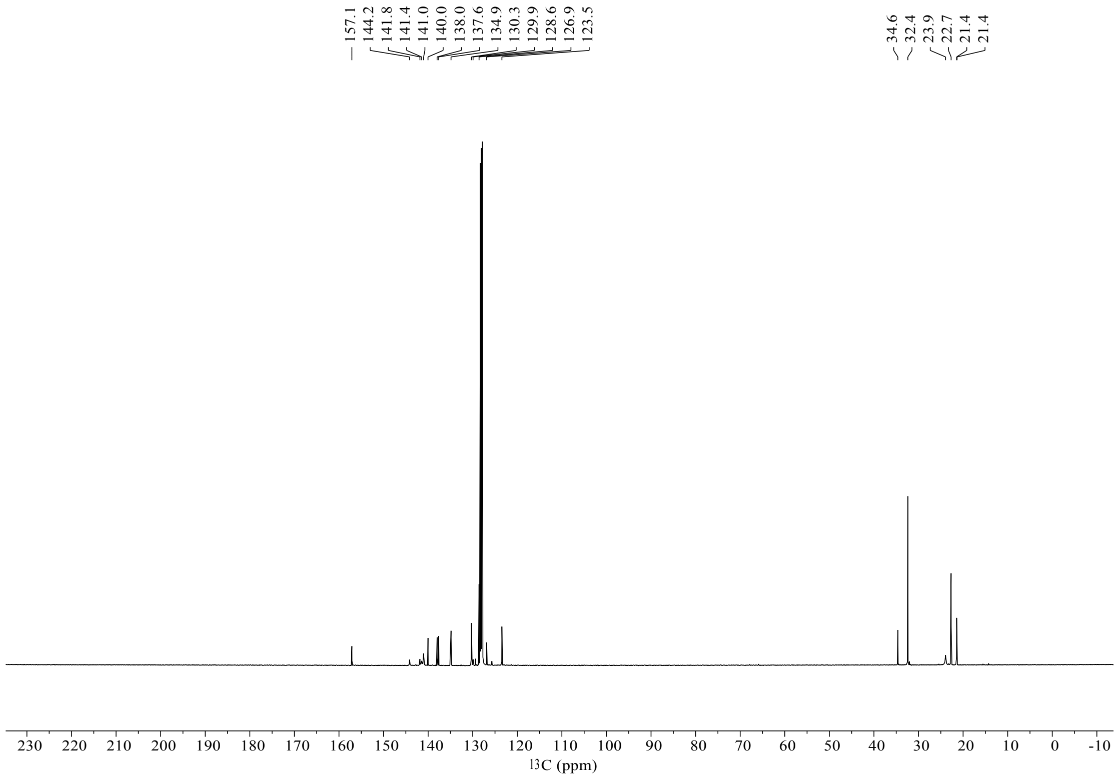

Figure S3. $^{13}C\{^{1}H\}$ NMR spectrum of **LLi** (**1**) in C_6D_6.

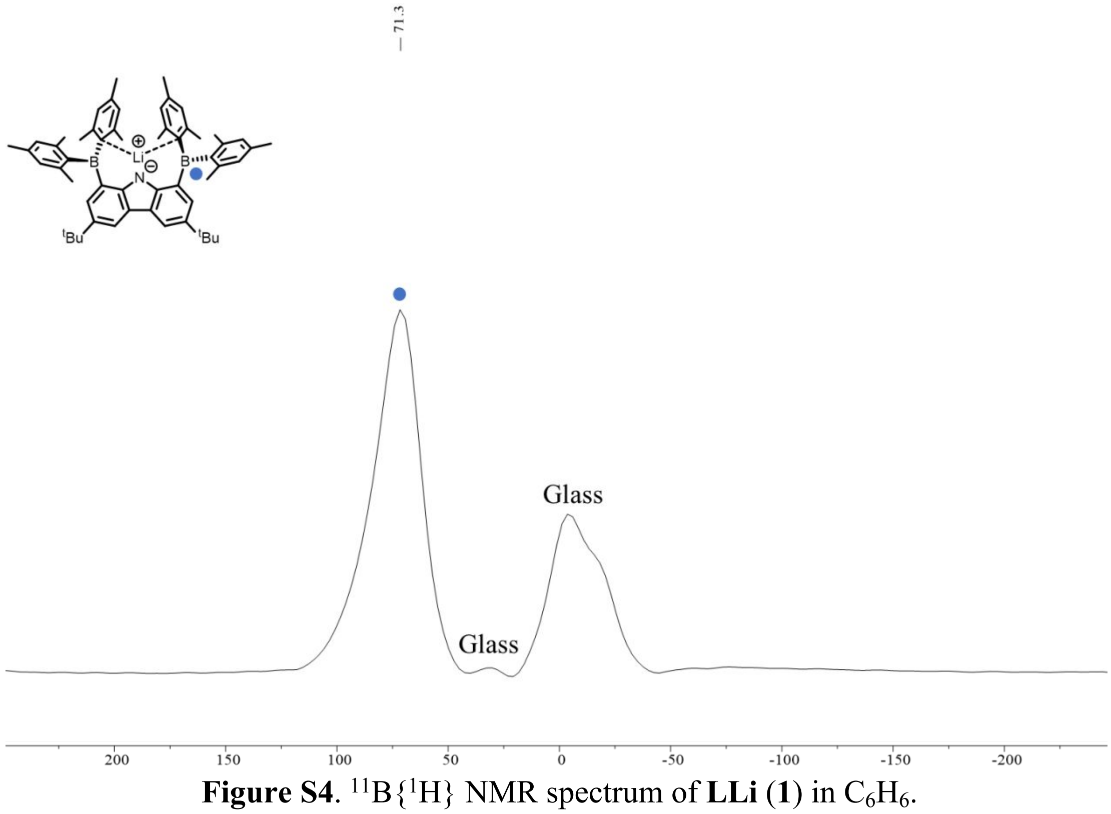

Figure S4. $^{11}B\{^{1}H\}$ NMR spectrum of **LLi** (**1**) in C_6H_6.

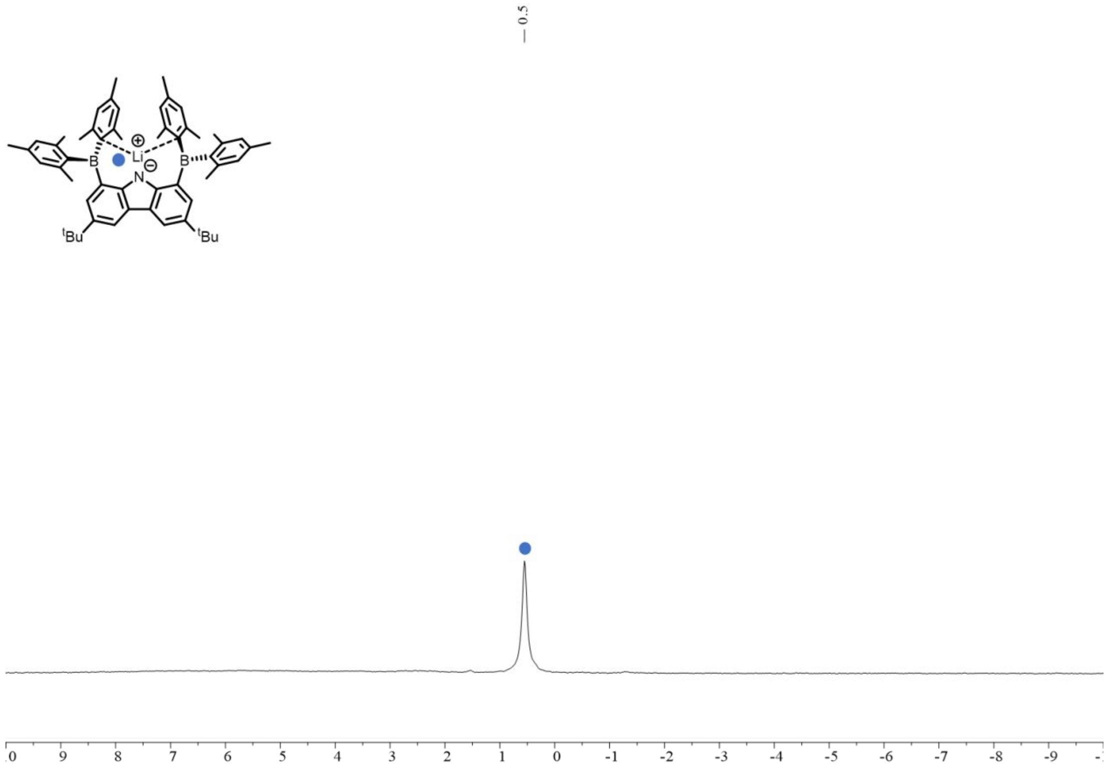

Figure S5. $^{7}Li\{^{1}H\}$ NMR spectrum of **LLi** (**1**) in C_6D_6.

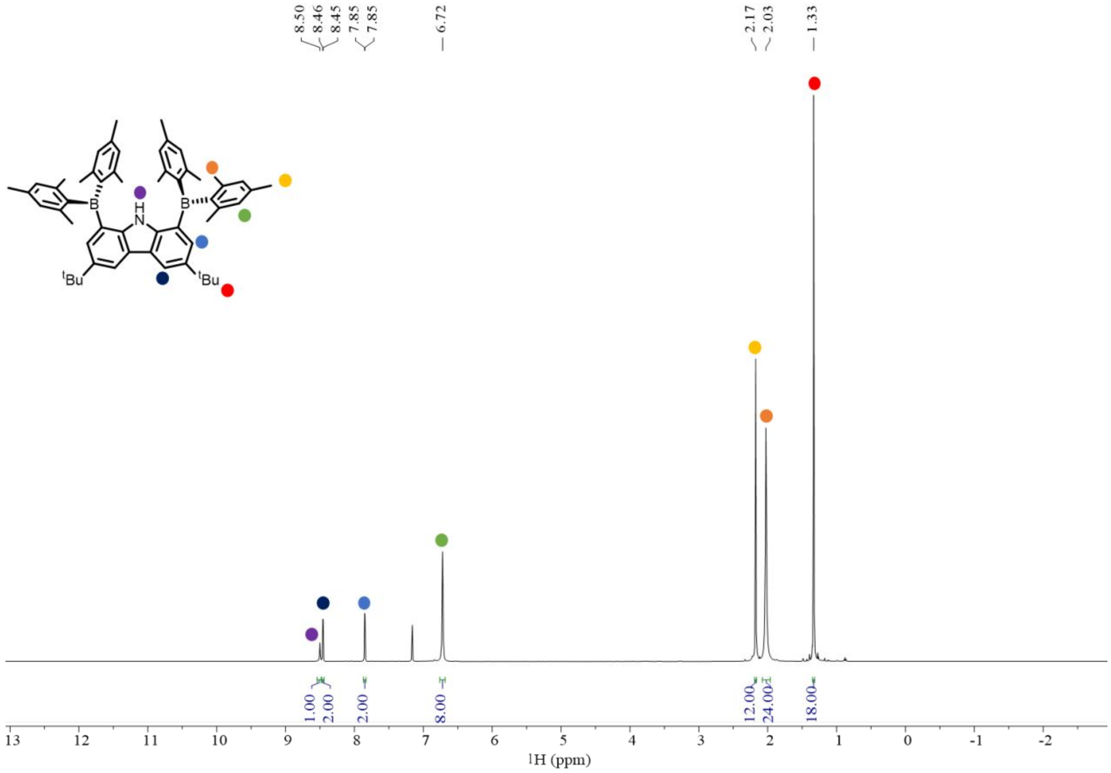

Figure S6. ^{1}H NMR spectrum of **LH** (**1-H**) in C_6D_6.

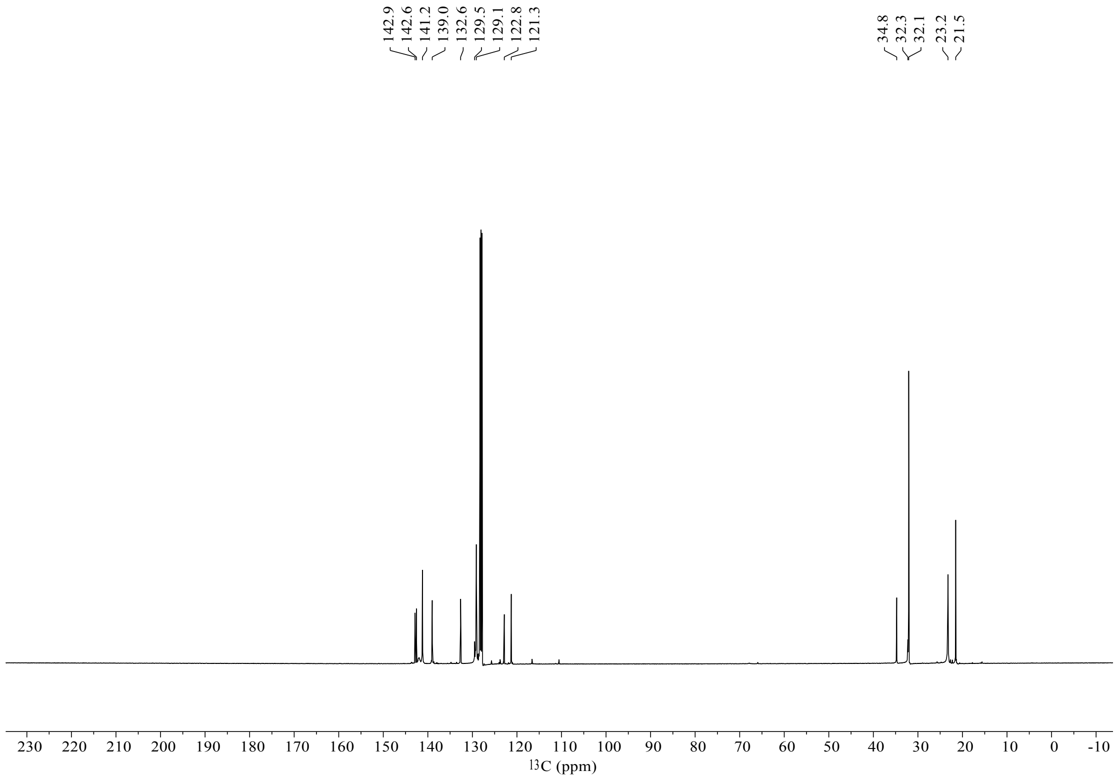

Figure S7. $^{13}C\{^1H\}$ NMR spectrum of **LH** (**1-H**) in C_6D_6.

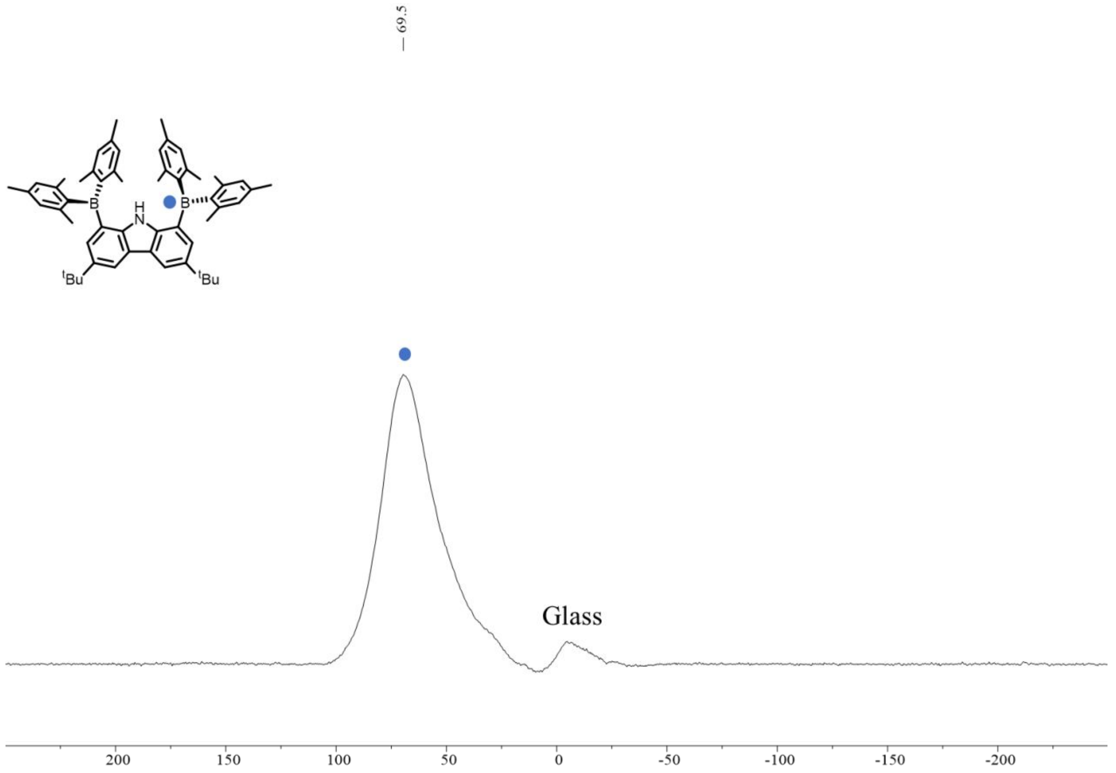

Figure S8. $^{11}B\{^1H\}$ NMR spectrum of **LH** (**1-H**) in C_6D_6.

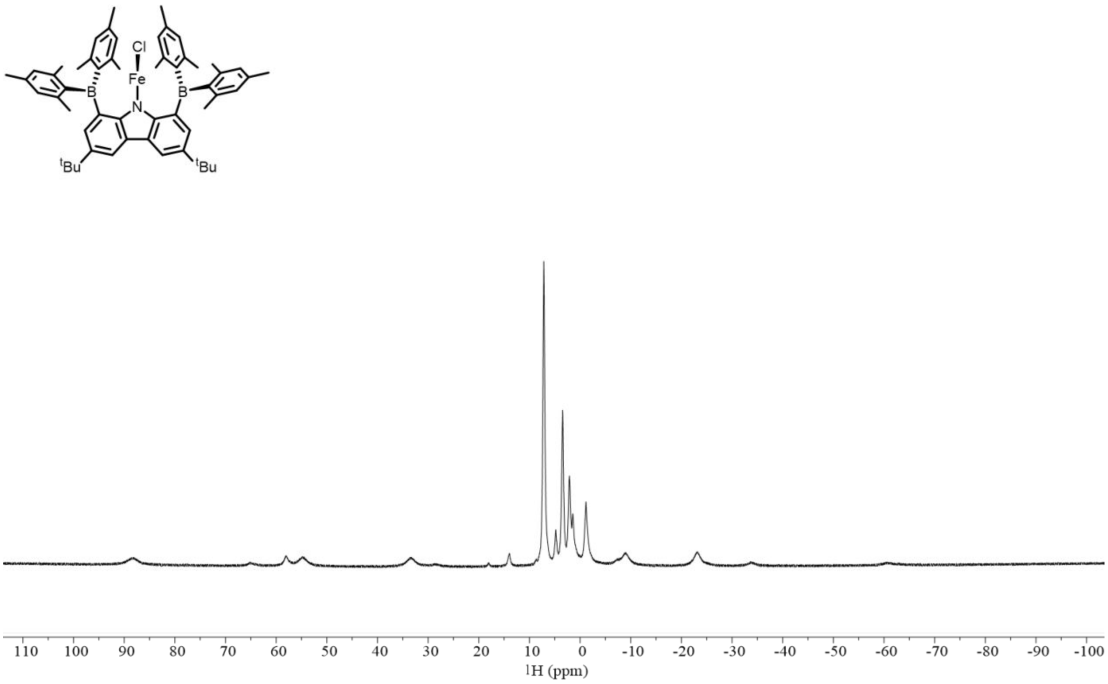

Figure S9. ^{1}H NMR spectrum of **LFeCl** (**2**) in C_6D_6.

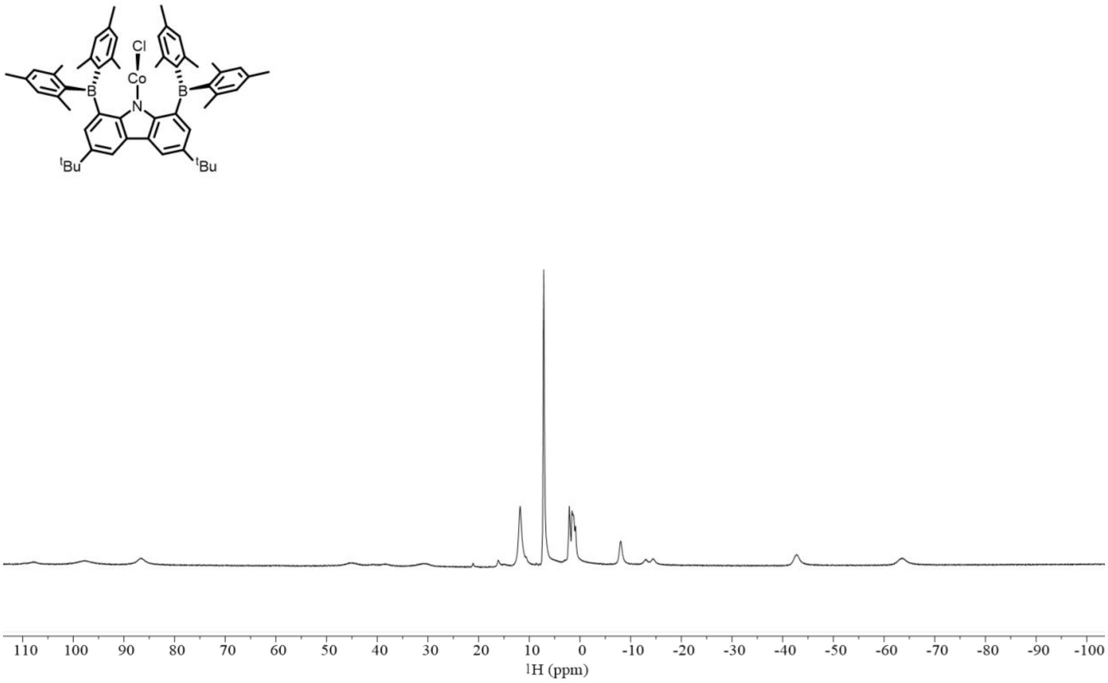

Figure S10. ^{1}H NMR spectrum of **LCoCl** (**3**) in C_6D_6.

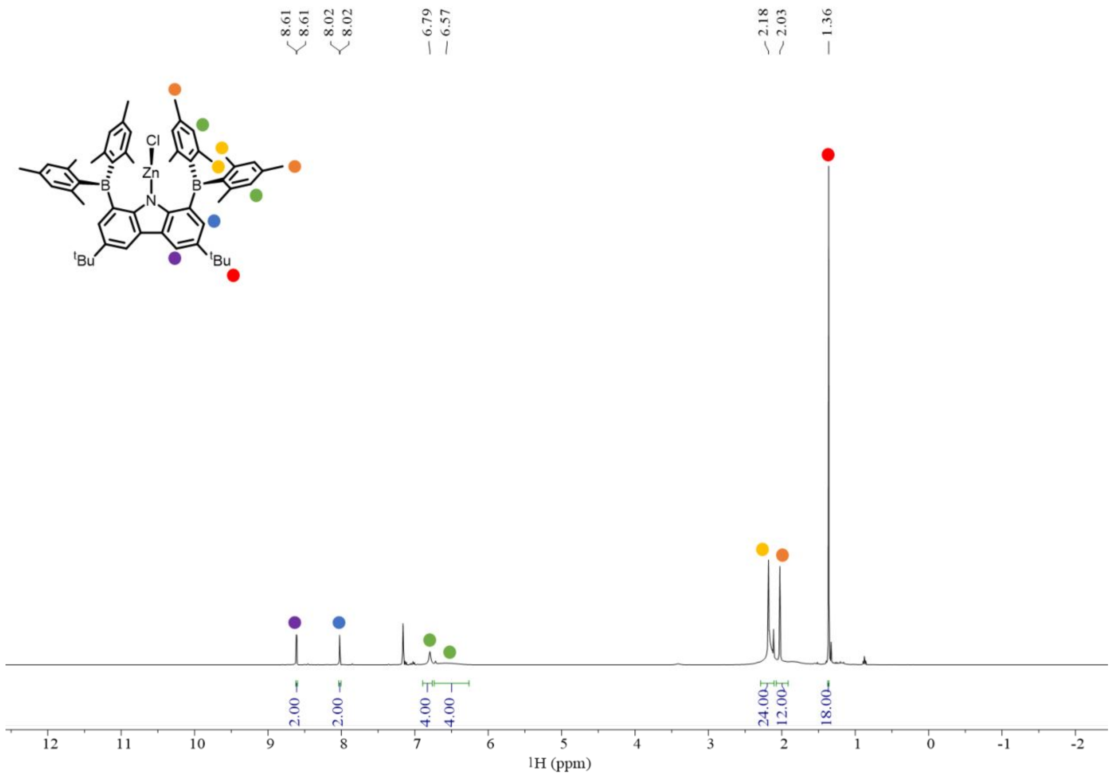

Figure S11. ^{1}H NMR spectrum of **LZnCl** (**4**) in C_6D_6.

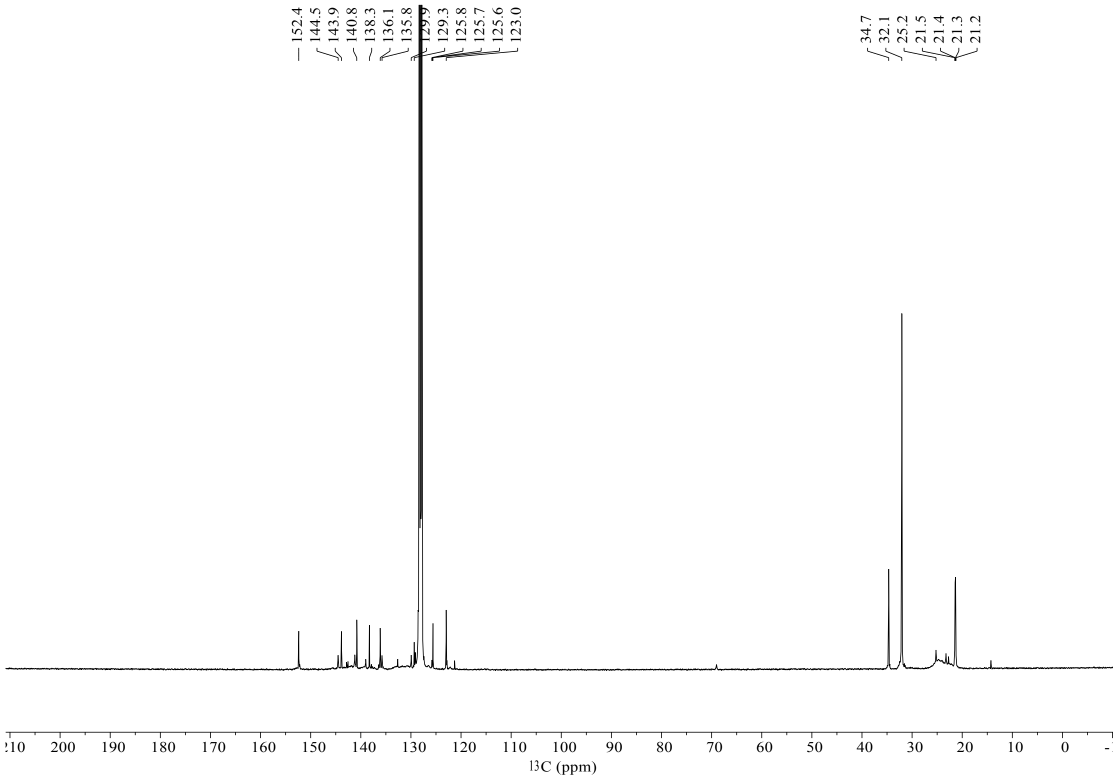

Figure S12. $^{13}C\{^{1}H\}$ NMR spectrum of **LZnCl** (**4**) in C_6D_6.

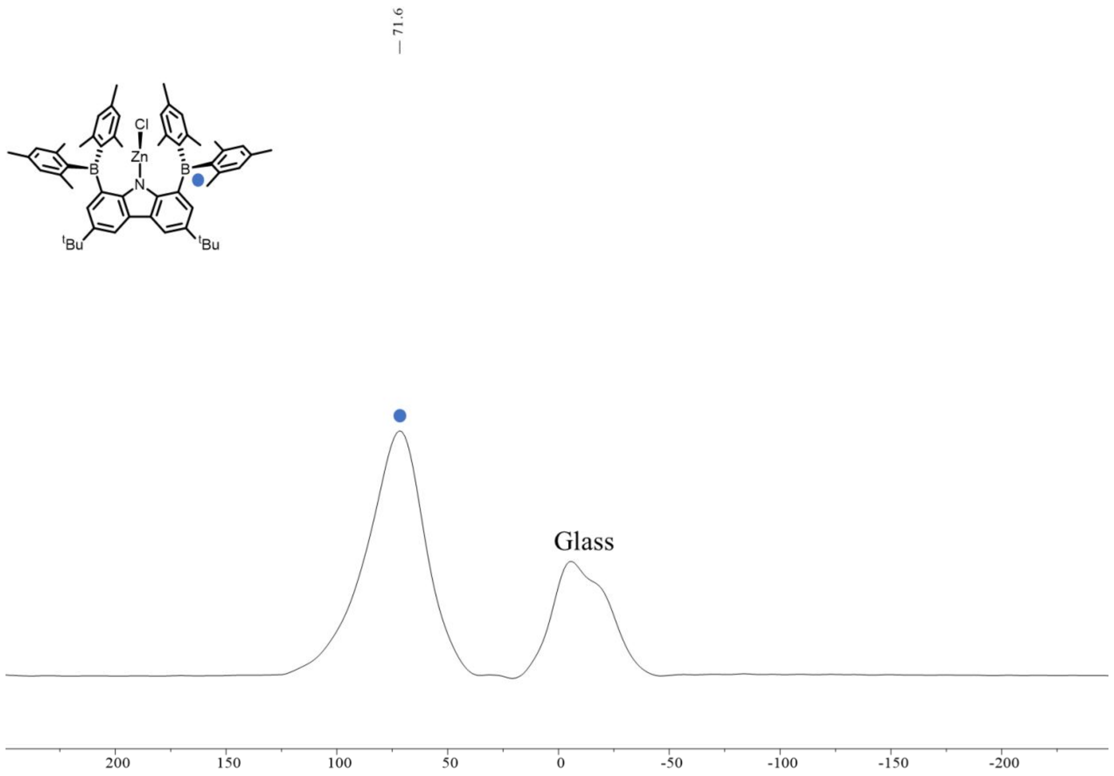

Figure S13. $^{11}B\{^{1}H\}$ NMR spectrum of **LZnCl** (**4**) in C_6D_6.

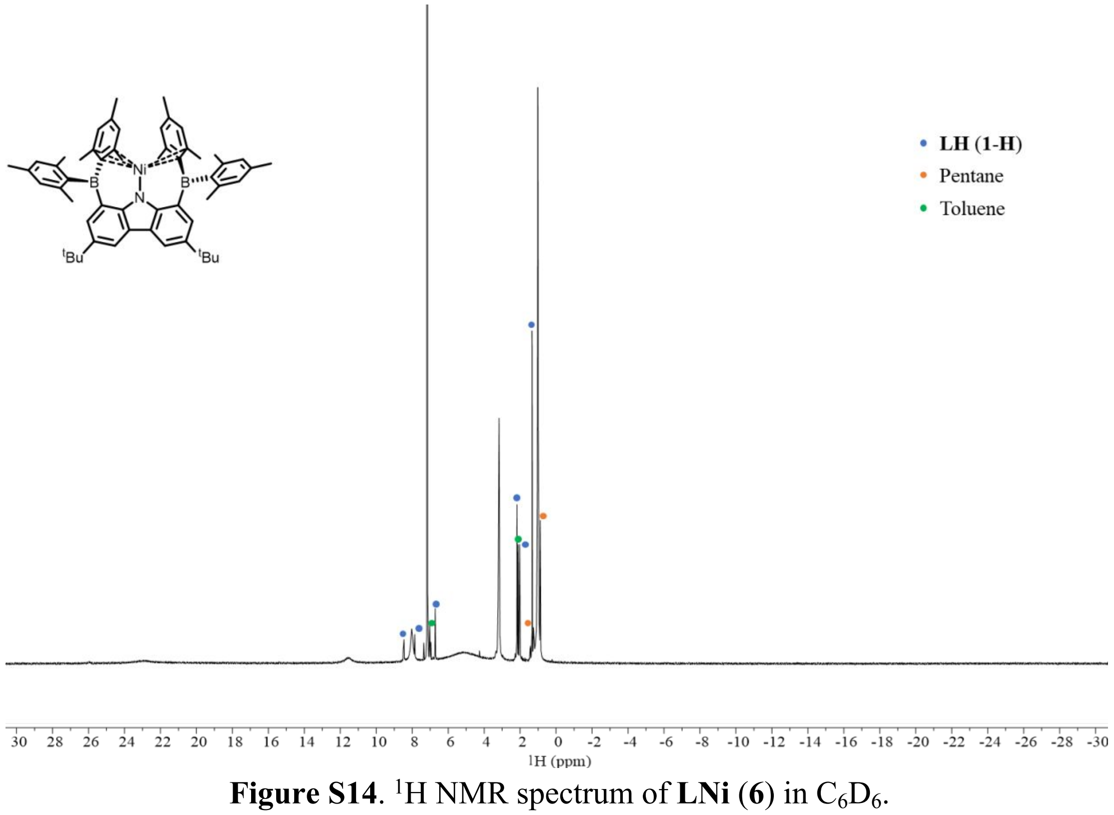

Figure S14. ^{1}H NMR spectrum of **LNi** (**6**) in C_6D_6.

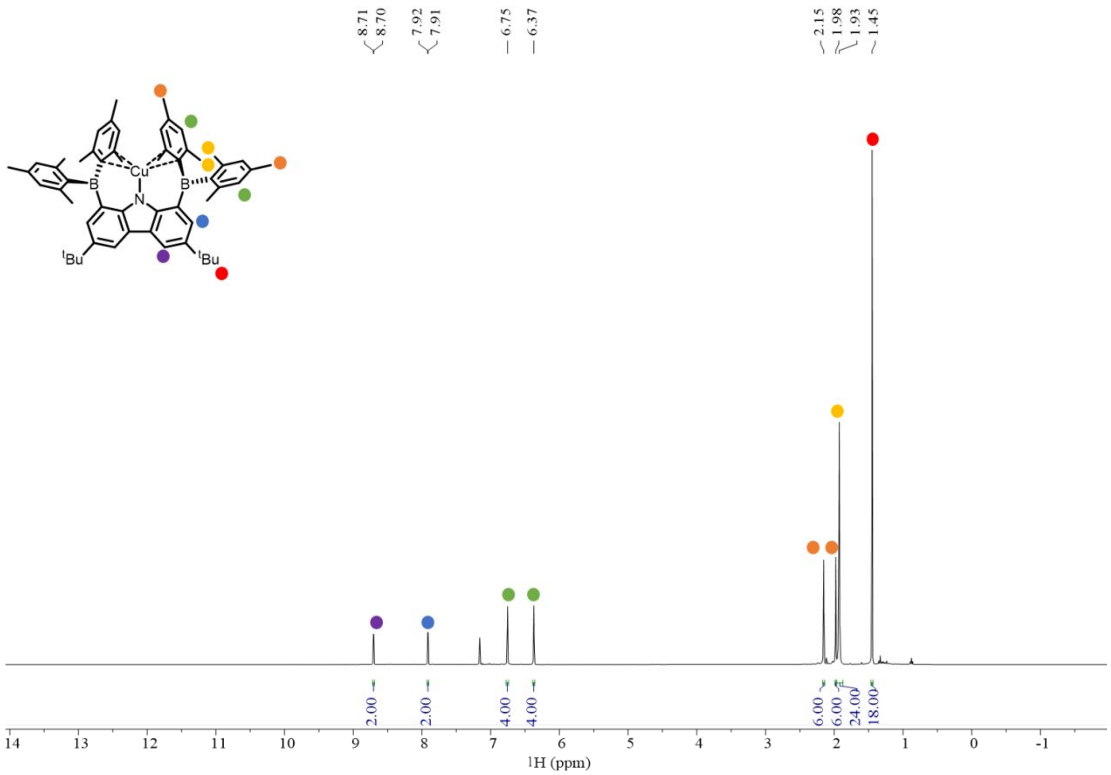

Figure S15. ^{1}H NMR spectrum of **LCu** (**7**) in C_6D_6.

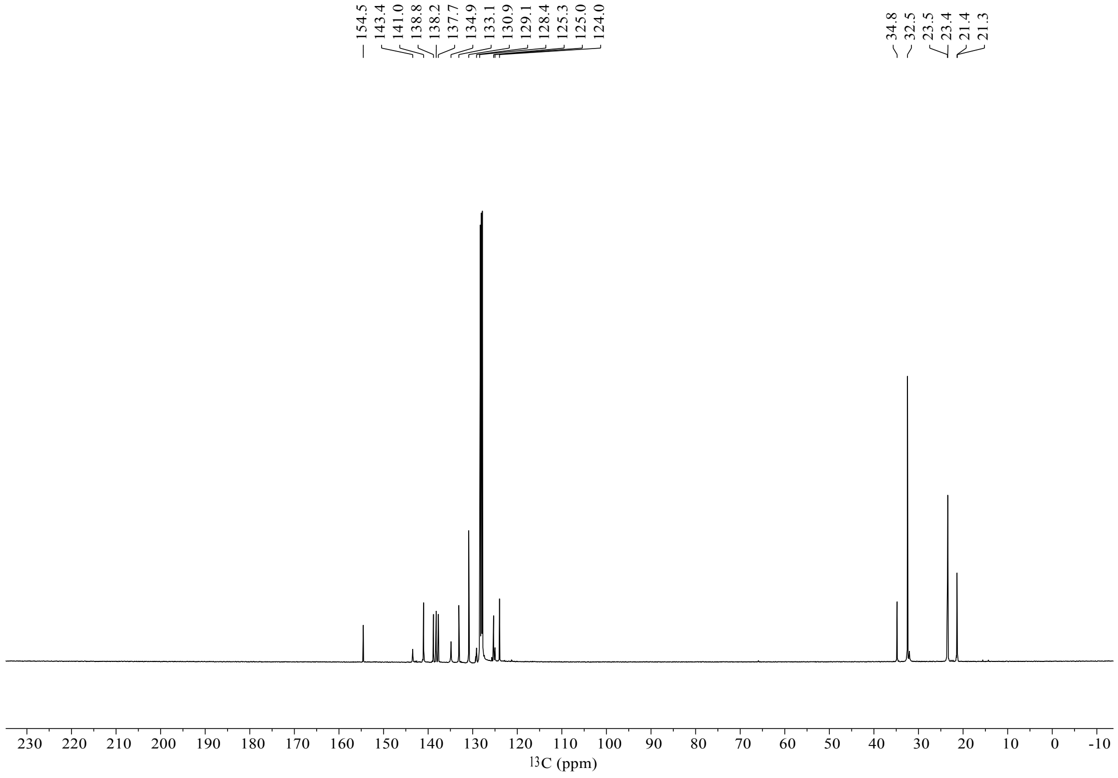

Figure S16. $^{13}C\{^{1}H\}$ NMR spectrum of **LCu** (**7**) in C_6D_6.

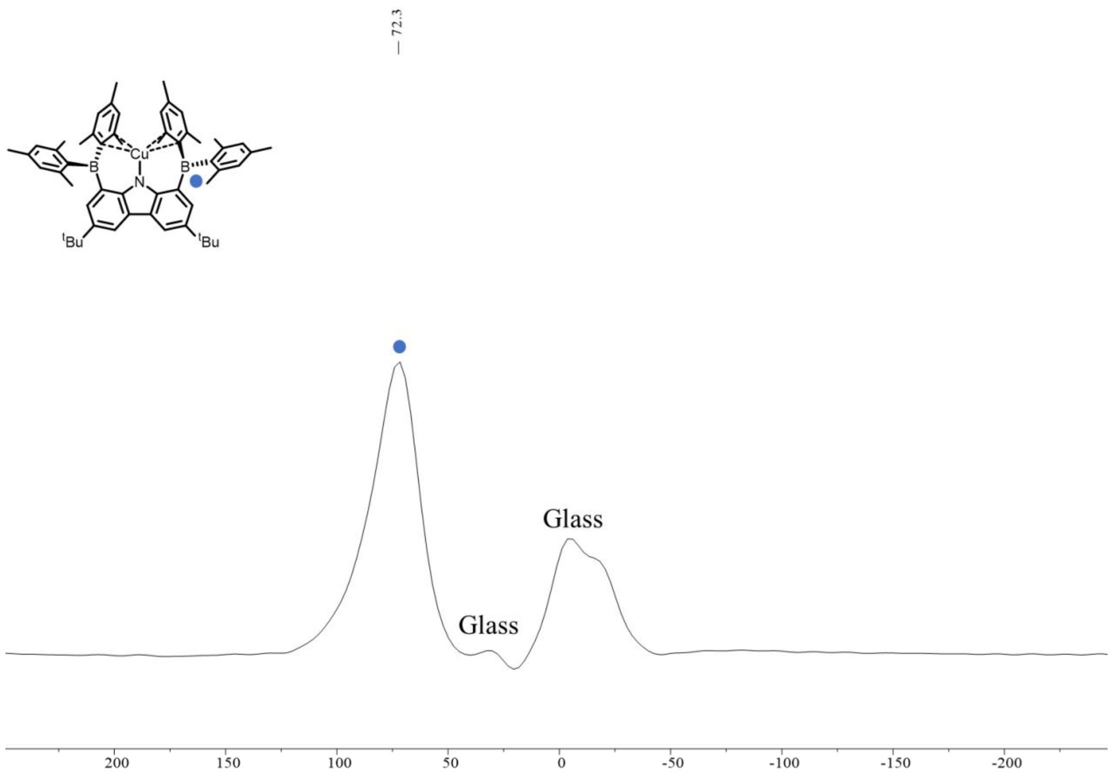

Figure S17 $^{11}B\{^1H\}$ NMR spectrum of **LCu** (**7**) in C_6H_6.

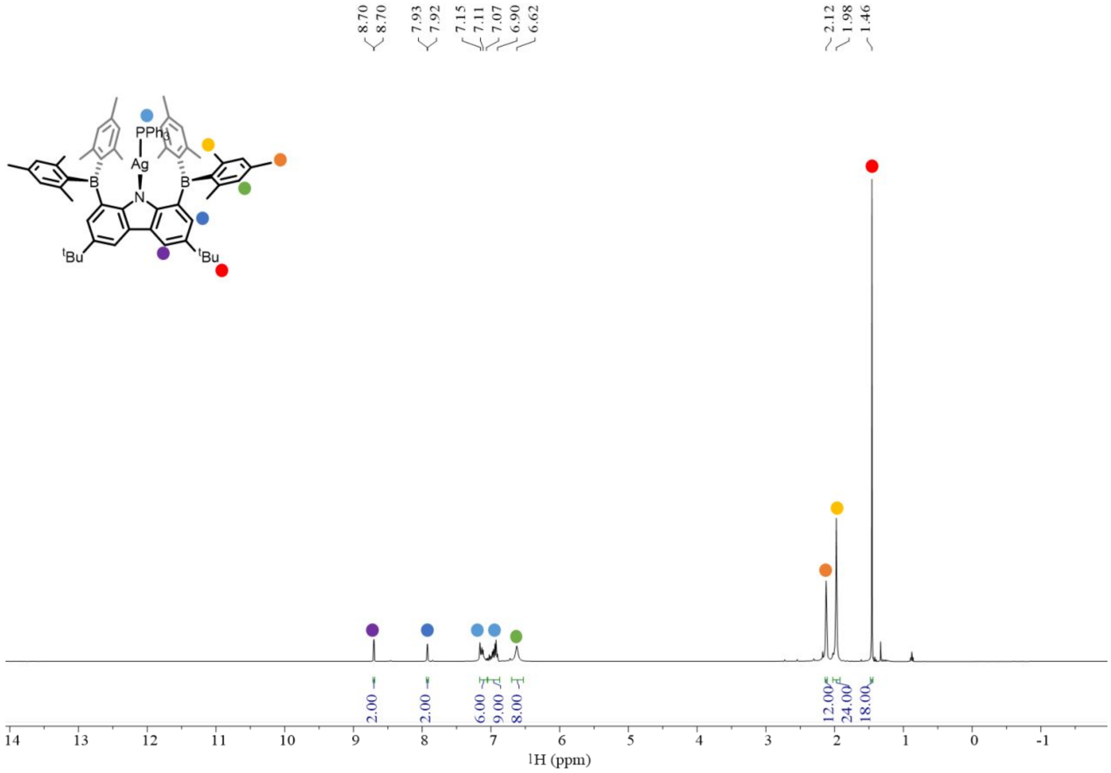

Figure S18. ^1H NMR spectrum of **LAgPPh$_3$** (**8**) in C_6D_6.

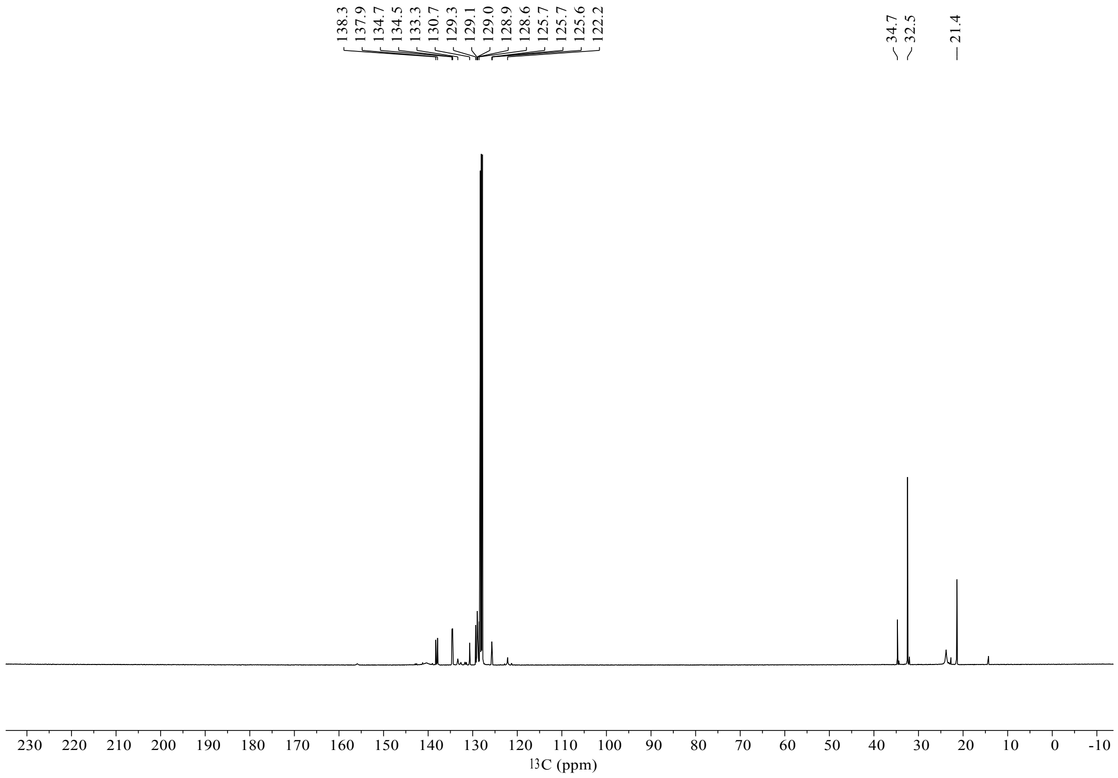

Figure S19. $^{13}C\{^1H\}$ NMR spectrum of **LAgPPh$_3$** (**8**) in C_6D_6.

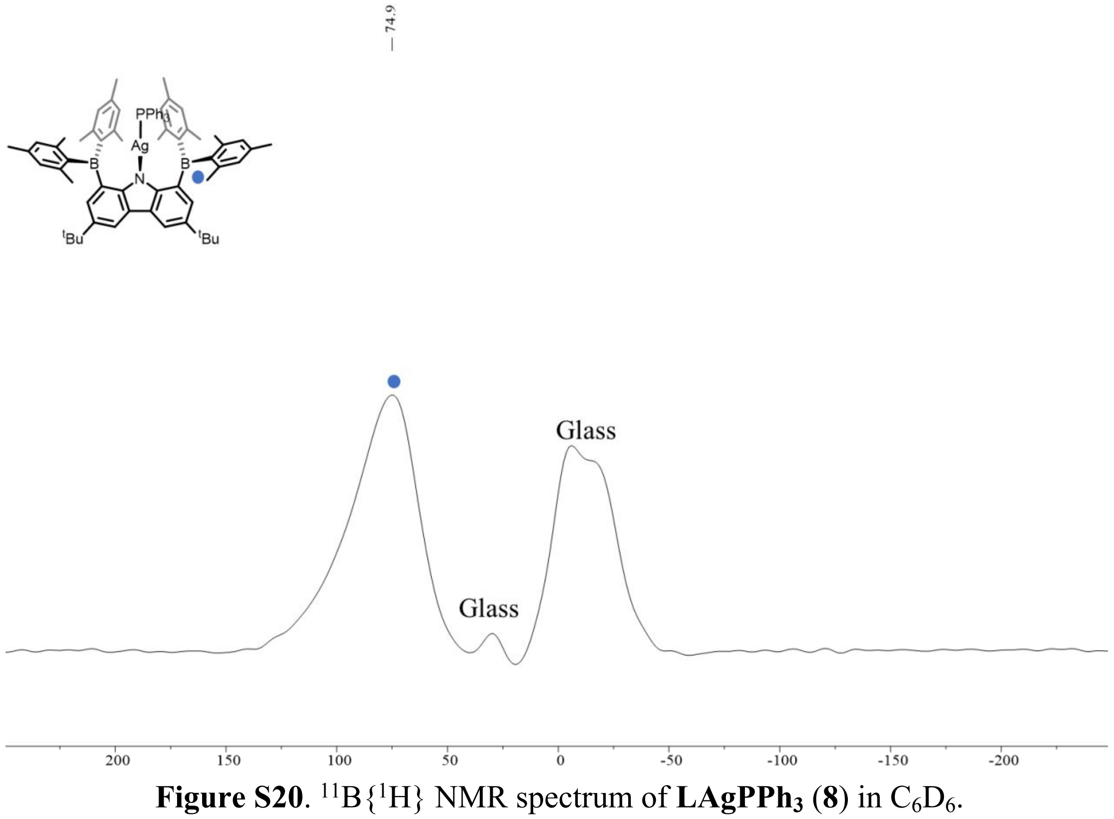

Figure S20. $^{11}B\{^1H\}$ NMR spectrum of **LAgPPh$_3$** (**8**) in C_6D_6.

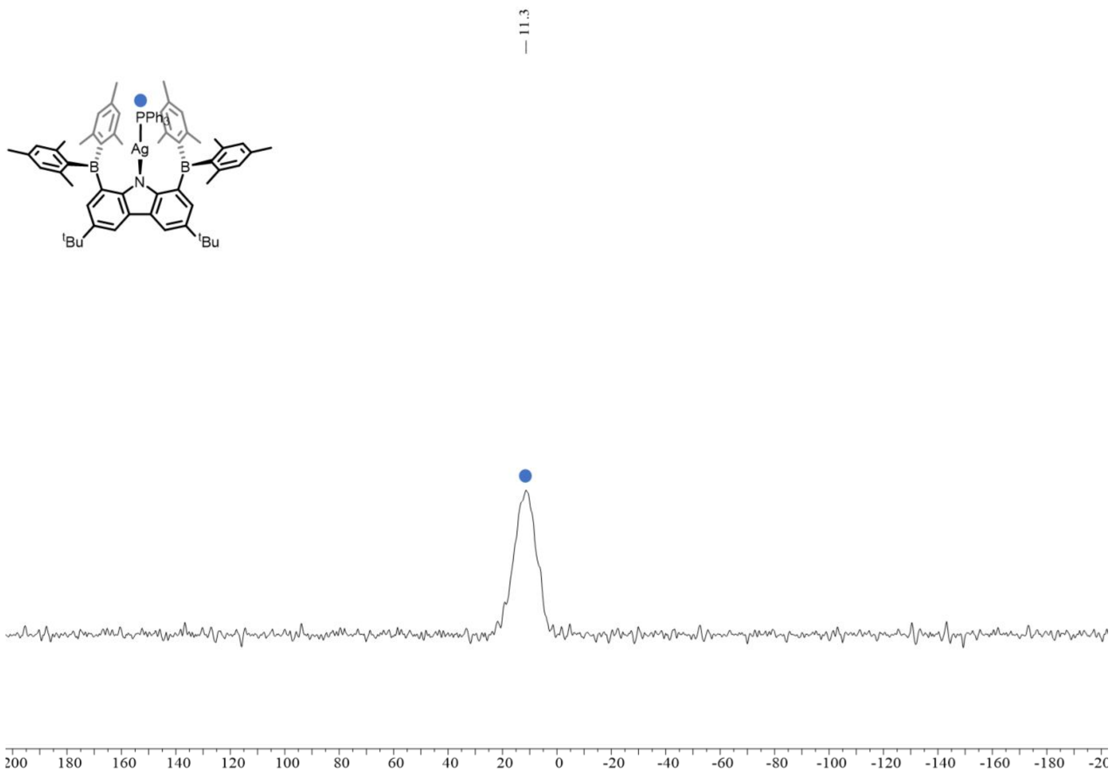

Figure S21. $^{31}P\{^1H\}$ NMR spectrum of **LAgPPh$_3$** (**8**) in C_6D_6.

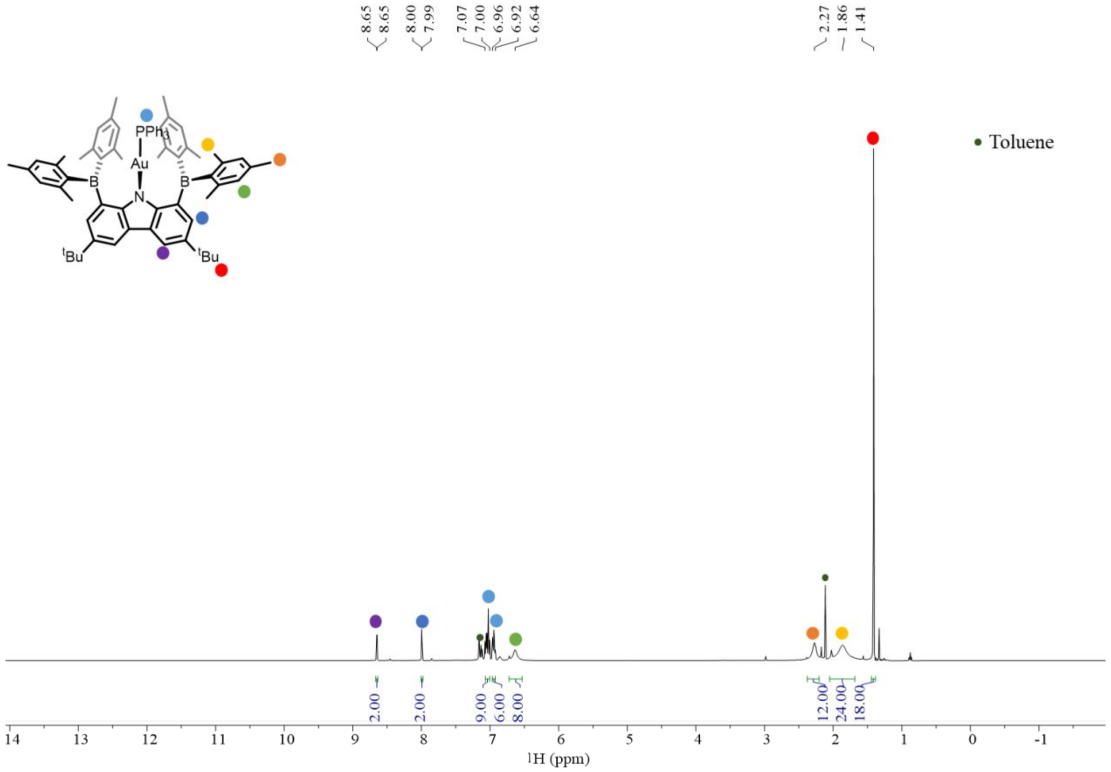

Figure S22. ^1H NMR spectrum of **LAuPPh$_3$** (**9**) in C_6D_6.

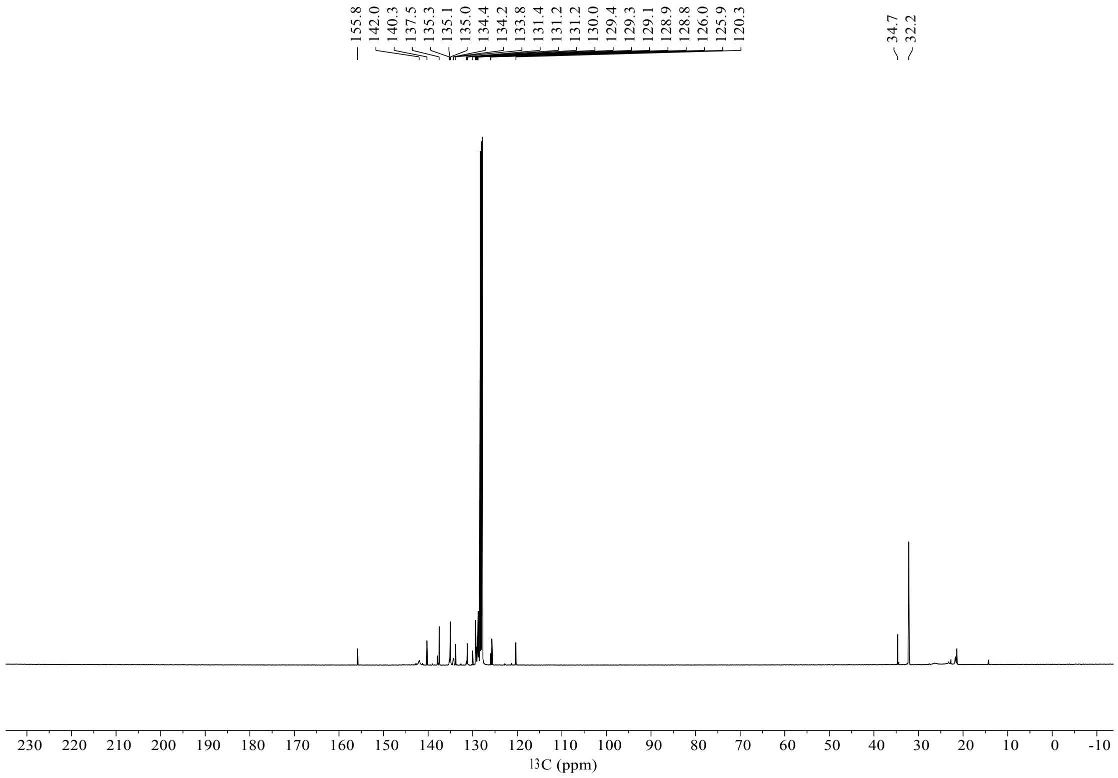

Figure S23. $^{13}C\{^{1}H\}$ NMR spectrum of **LAuPPh$_3$** (**9**) in C_6D_6.

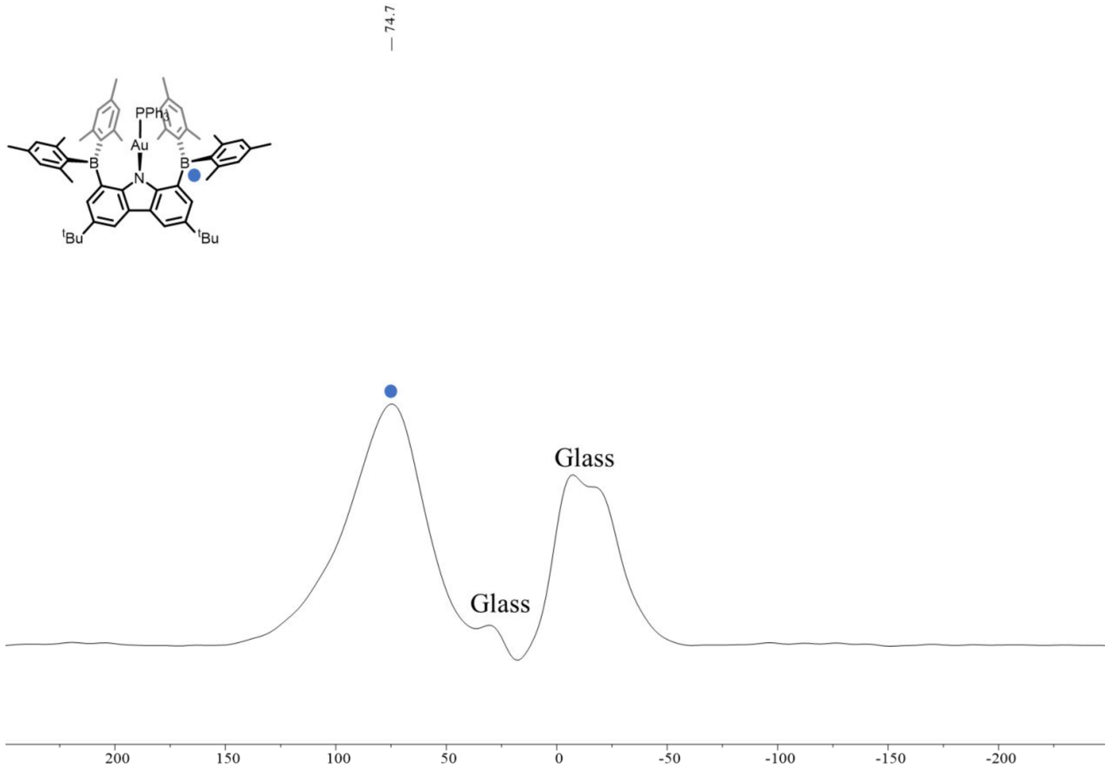

Figure S24. $^{11}B\{^{1}H\}$ NMR spectrum of **LAuPPh$_3$** (**9**) in C_6D_6.

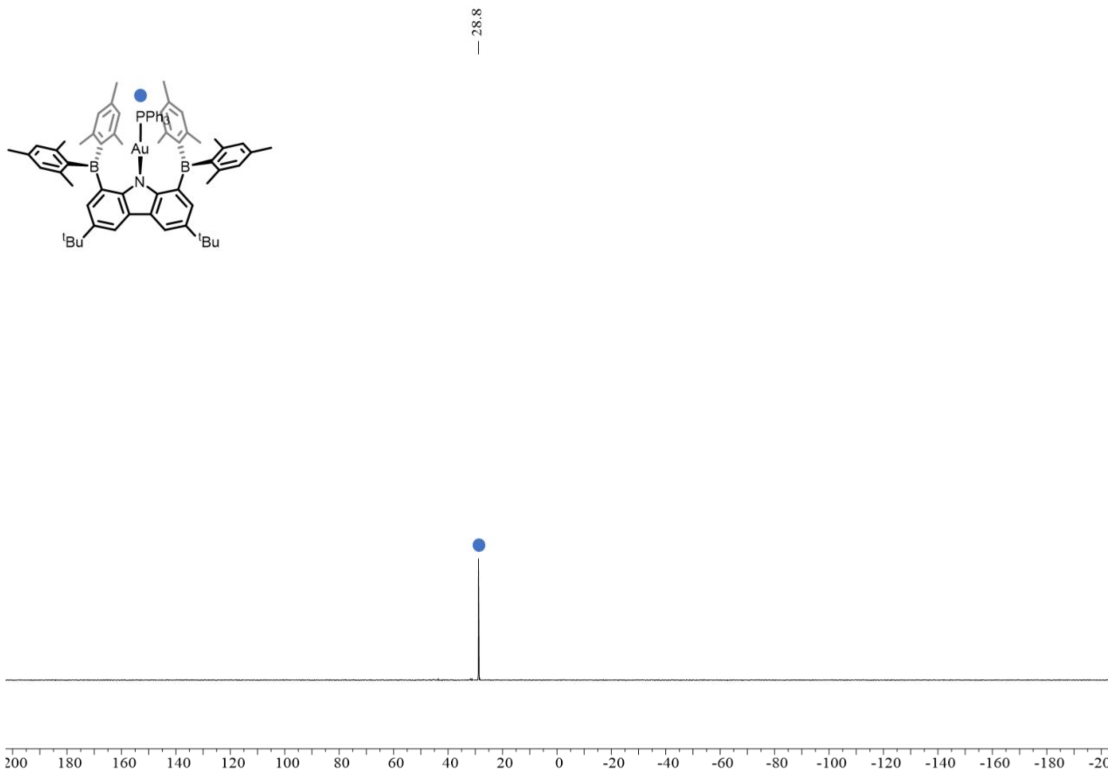

Figure S25. $^{31}P\{^{1}H\}$ NMR spectrum of **LAuPPh$_3$** (**9**) in C_6D_6.

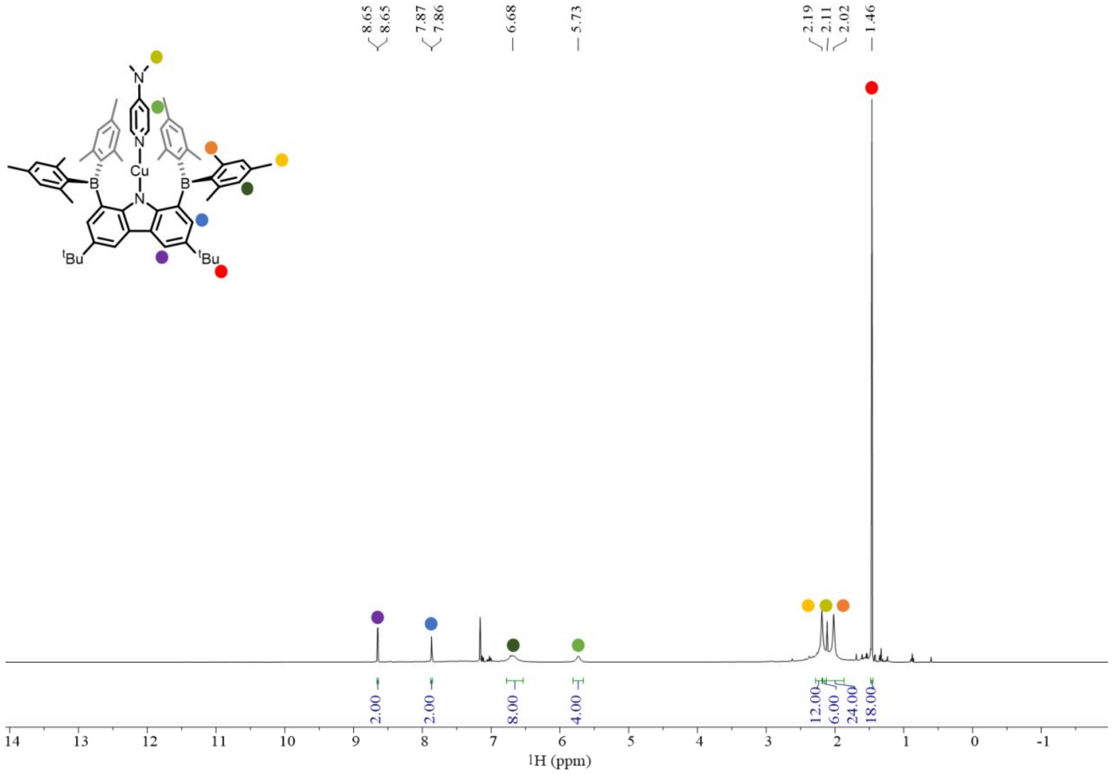

Figure S26. ^{1}H NMR spectrum of **LCu**(**DMAP**) (**10**) in C_6D_6.

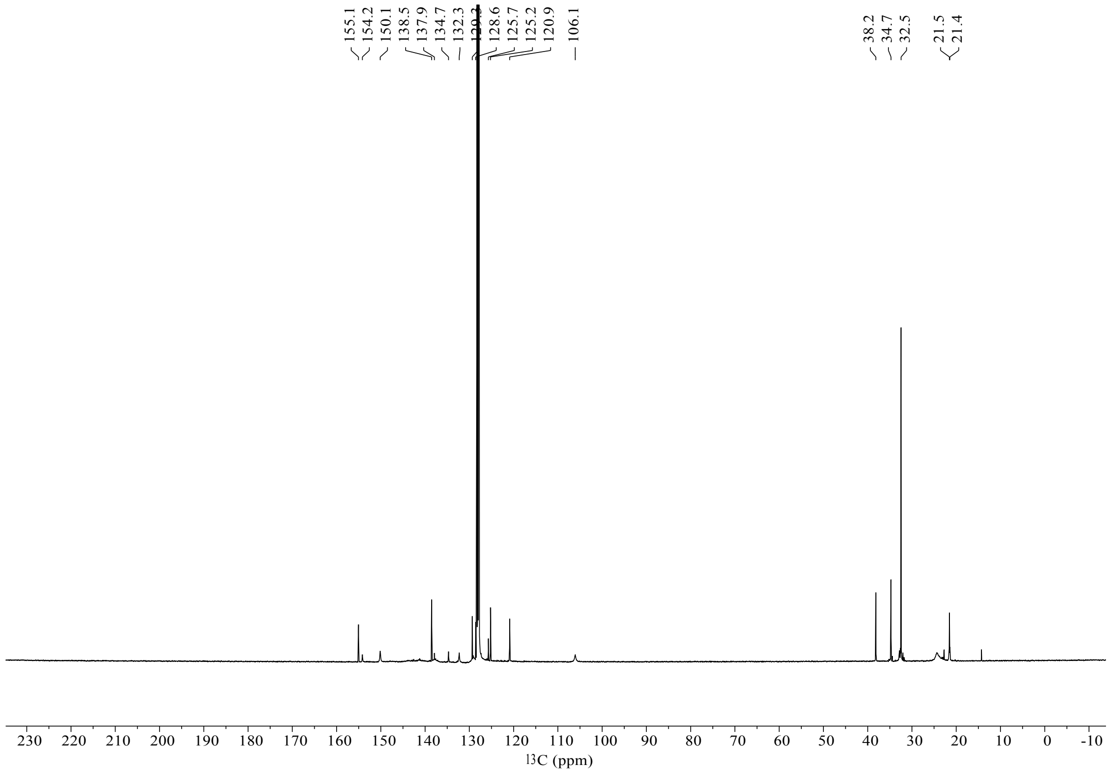

Figure S27. $^{13}C\{^{1}H\}$ NMR spectrum of **LCu**(**DMAP**) (**10**) in C_6D_6.

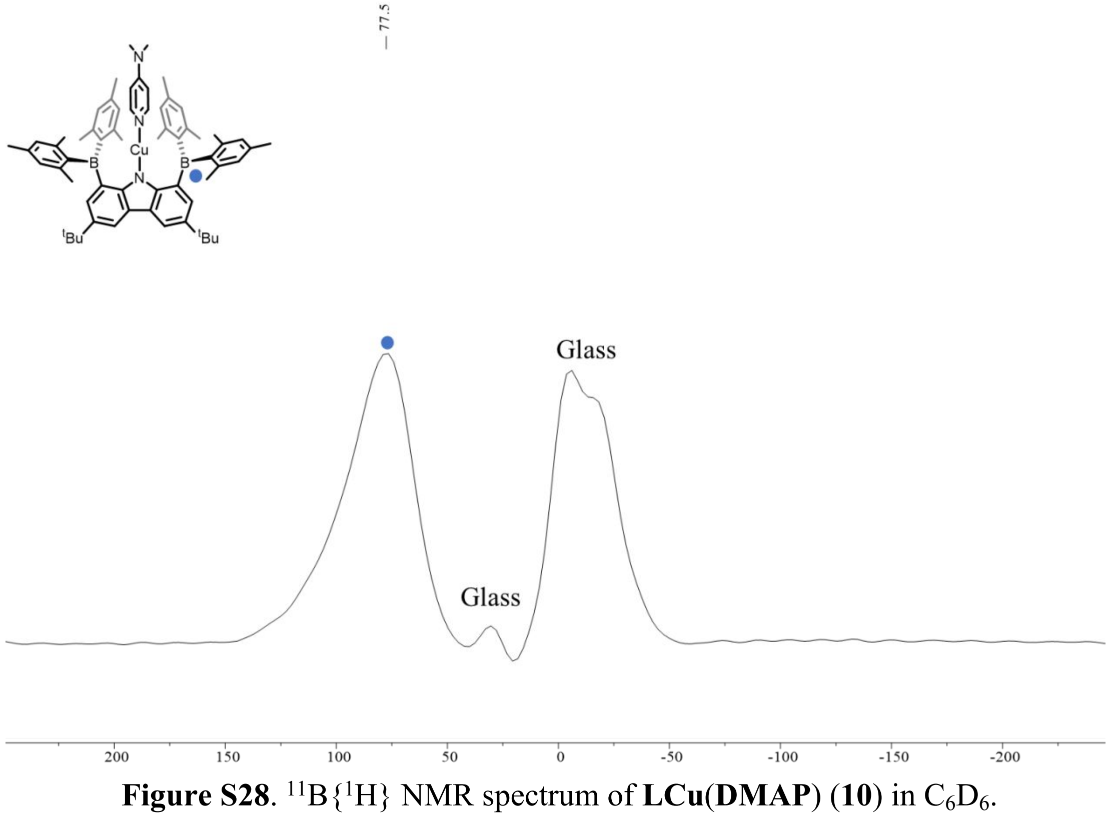

Figure S28. $^{11}B\{^{1}H\}$ NMR spectrum of **LCu**(**DMAP**) (**10**) in C_6D_6.

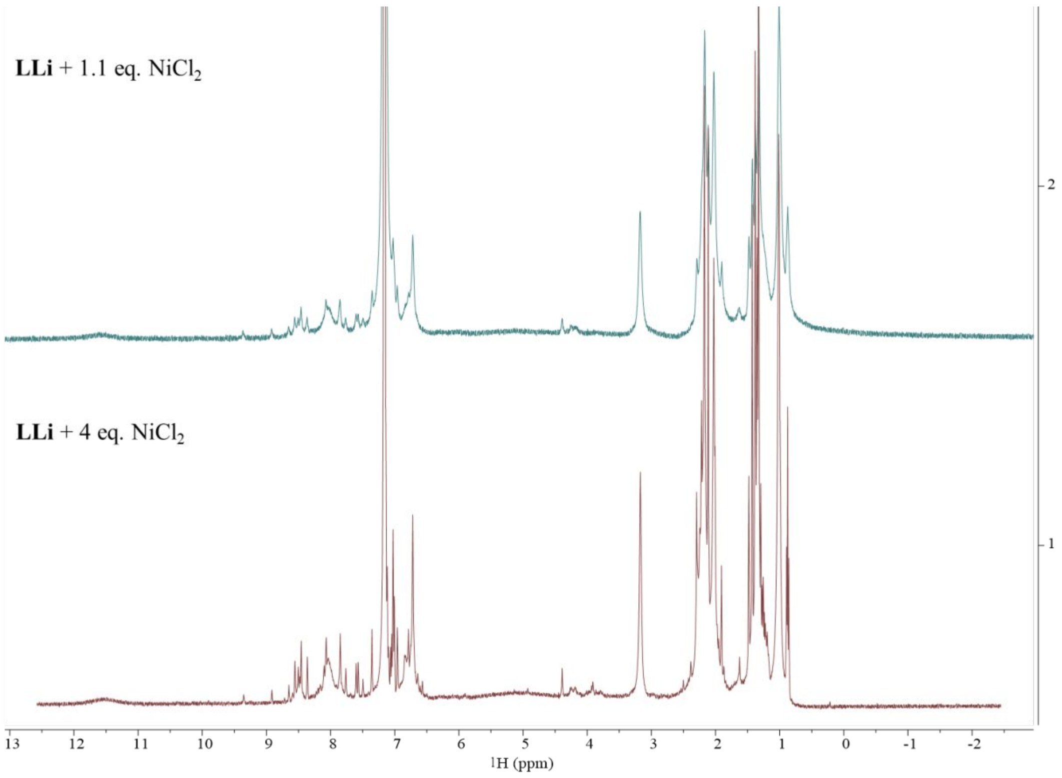

Figure S29. ^{1}H NMR spectrum in C_6D_6 of **LLi** (**1**) react with different equivalence of $NiCl_2$.

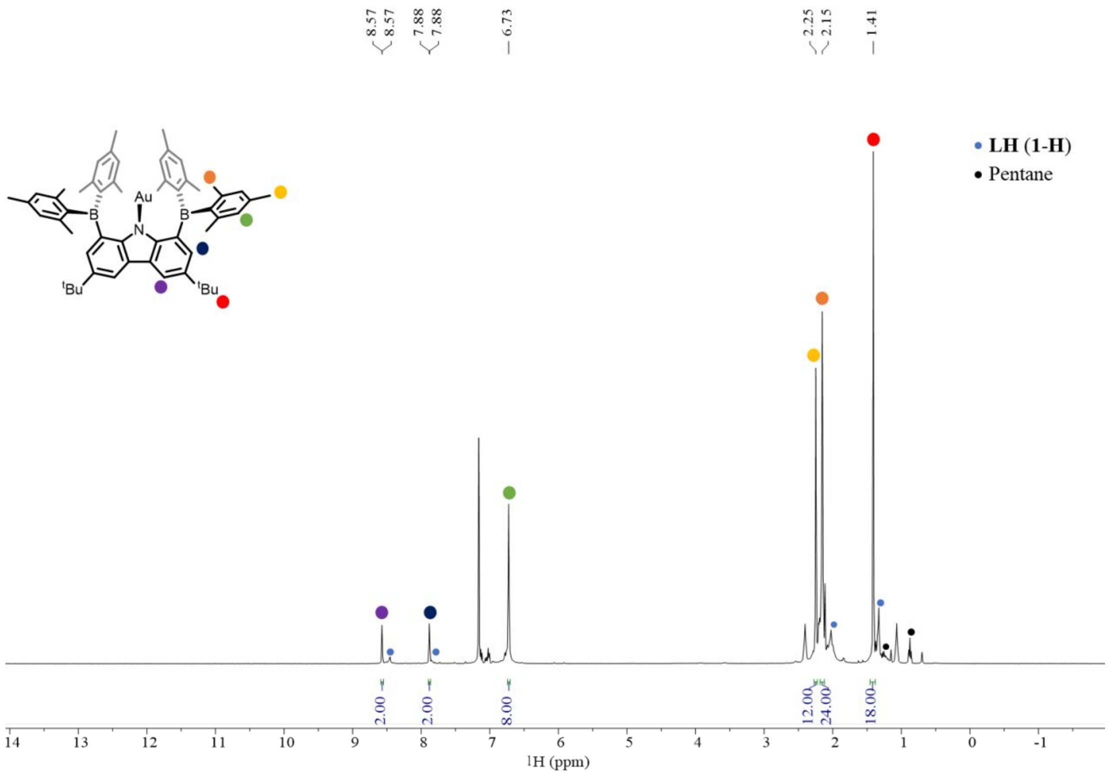

Figure S30. ^{1}H NMR spectrum of LAu in C_6D_6.

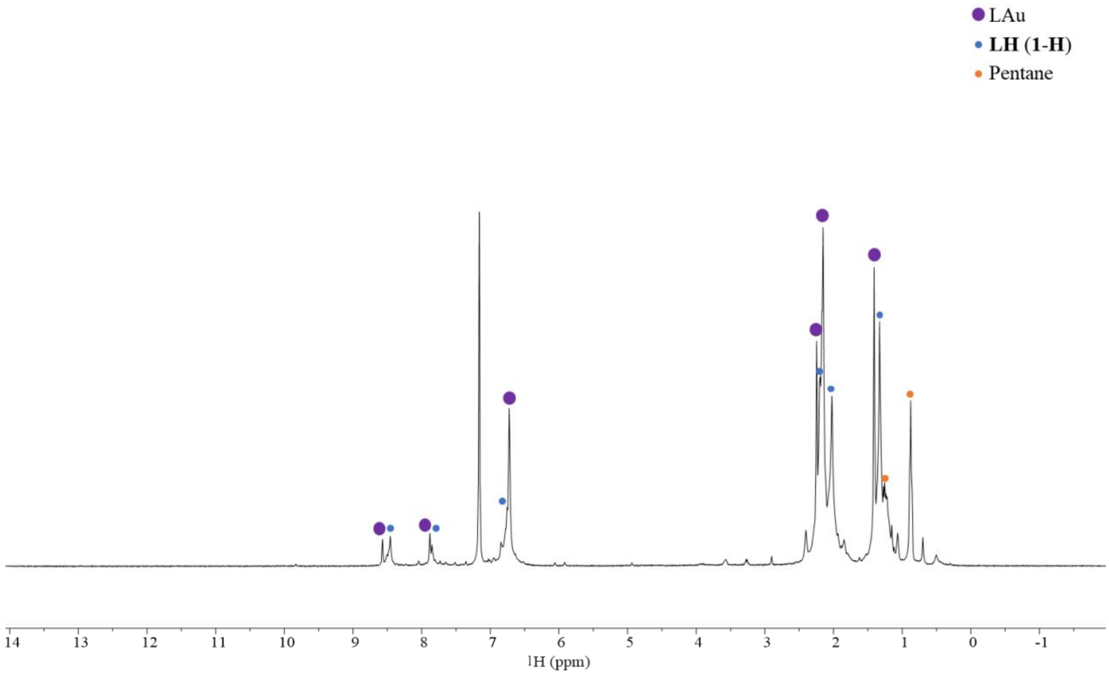

Figure S31. ^{1}H NMR spectrum of LAu slowly decomposed in C_6D_6.

2. EPR Characterizations

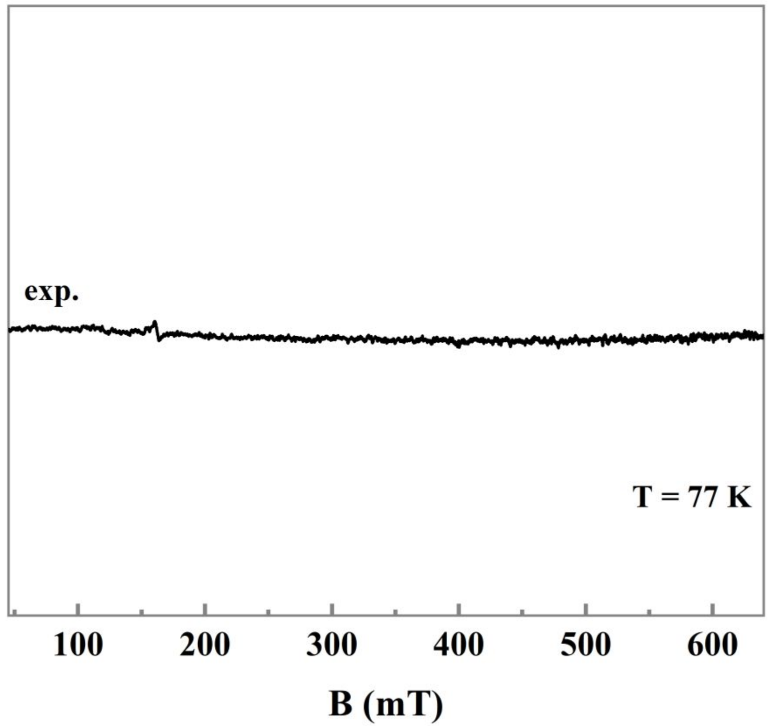

Figure S32. CW X-band EPR spectrum of **LFeCl** (**2**); Temperature = 77 K, solvent = Toluene.

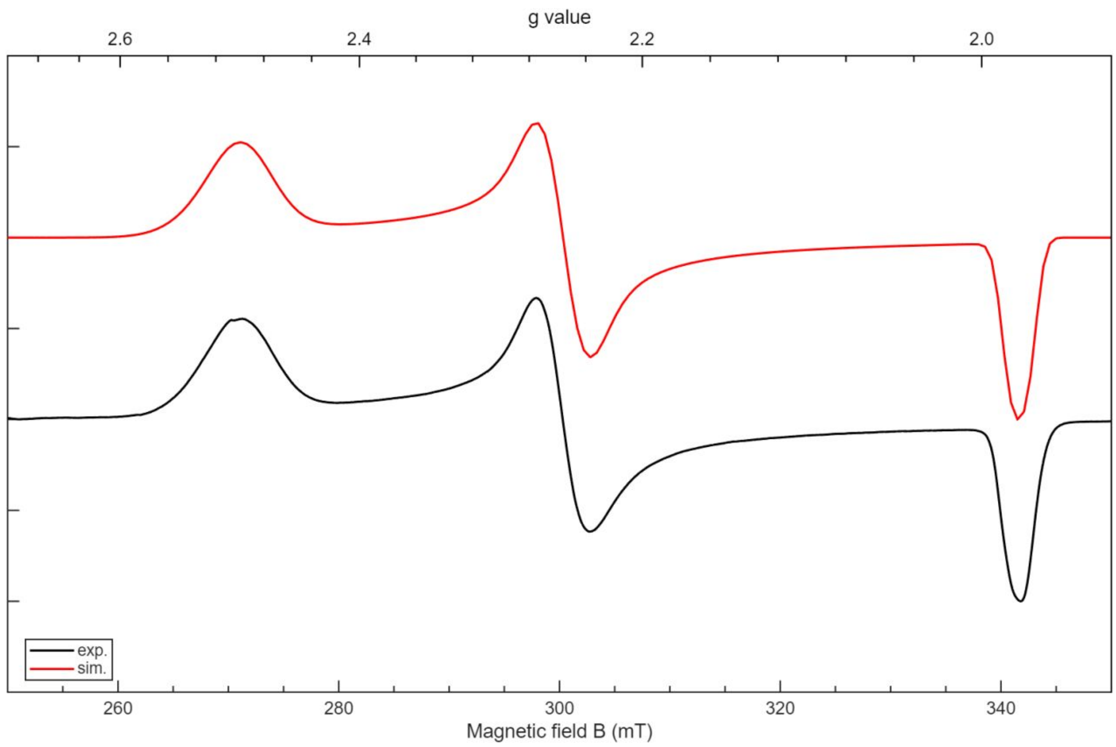

Figure S33. Experimental (black) and simulated (red) CW X-band EPR spectra of **LNi** (**6**). Temperature = 77 K, solvent = Toluene. Fit parameters: g_1 = 1.9805 ± 0.0001, g_2 = 2.2528 ± 0.0001, and g_3 = 2.5006 ± 0.0002; A_1 = 28.3 ± 0.4 MHz, A_2 = 0 MHz, and A_3 = 29.4 ± 3.2 MHz (^{14}N); g_{strain} = [0, 0.0157, 0.0415] ± [0, 0.0008, 0.06]. A_{strain} = [0, 0, 75.6] ± [0, 0, 100] MHz; H_{strain} = [47.3, 123, 191] ± [2.2, 0, 160] MHz; lw_{pp} = [0.0909, 0.240] ± [0, 0.026] mT.

$\underline{H_{strain,1} = 70}$

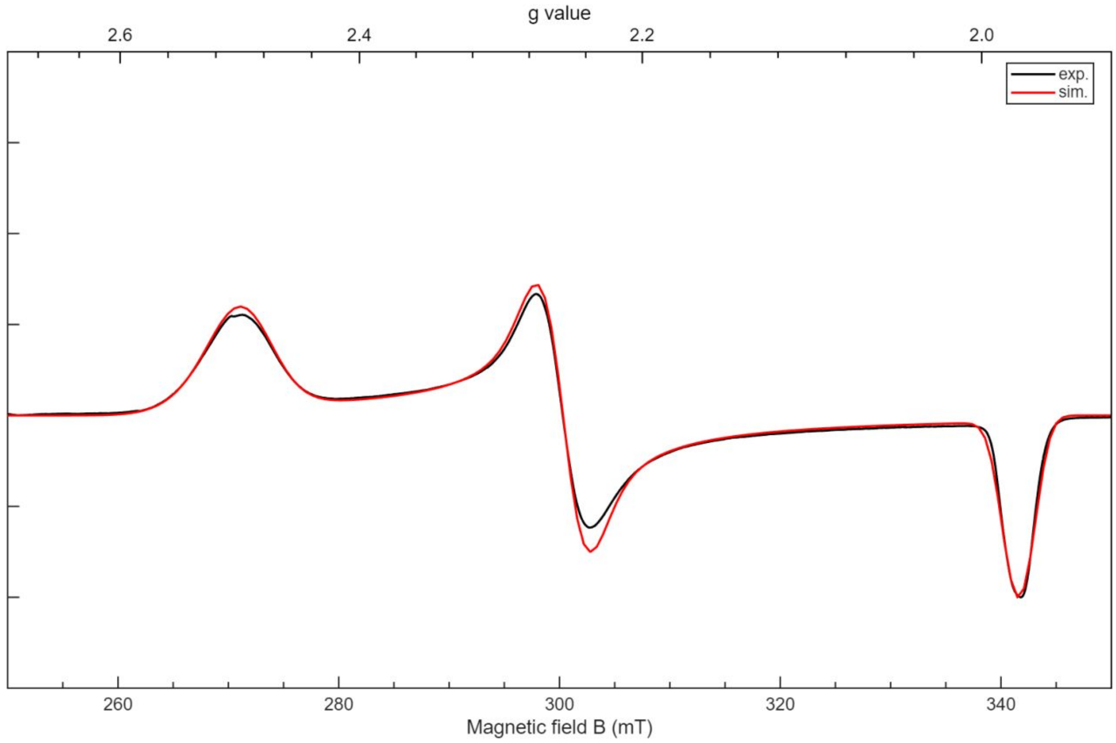

$\underline{H_{strain,1} = 30}$

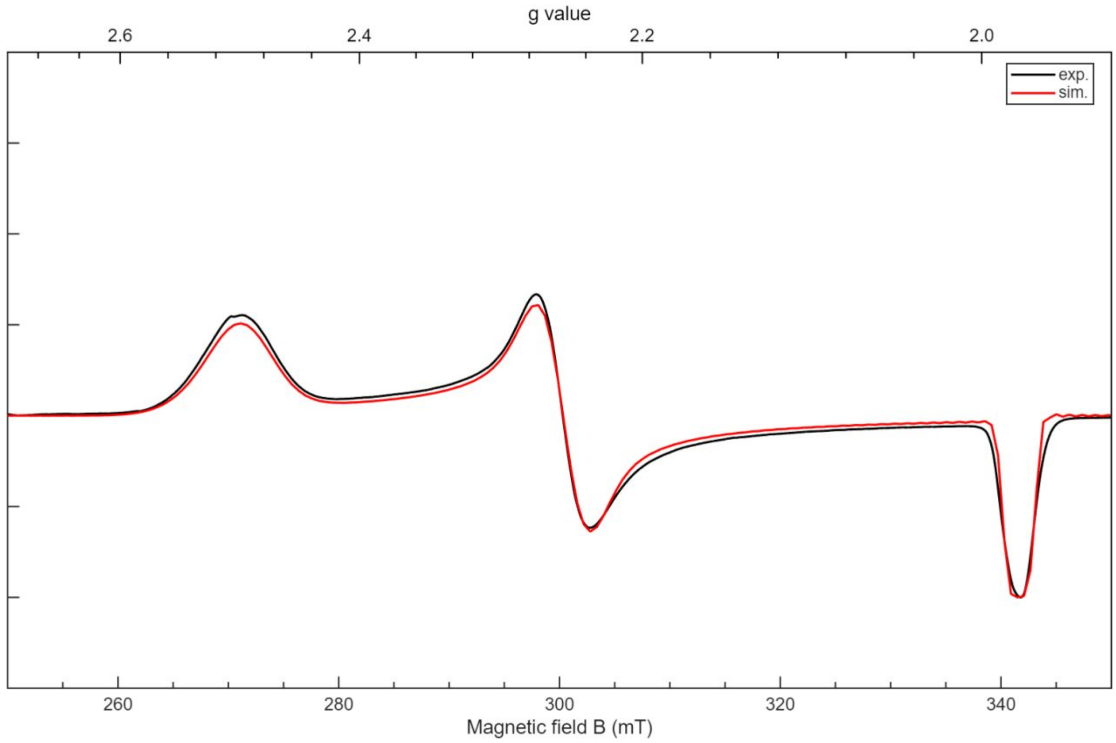

Figure S34. Experimental (black) and simulated (red) CW X-band EPR spectra of **LNi** (**6**) with different H_{strain} values; Temperature = 77 K, solvent = Toluene.

3. Single-Crystal X-Ray Diffraction Crystal Structure Figures and Data Tables

Single-crystal X-ray diffraction for complexes **1–4**, **7–10** and **1-H** were performed on a Bruker APEX DUO diffractometer with APEX II 4K and multi-layer mirror monochromated Mo Kα radiation (λ = 0.71073 Å) at 200(2) K. Data collection and reduction were performed with Bruker APEX II software. All of nonhydrogen atoms are refined anisotropically. Hydrogen atoms attached to the carbons were fixed at calculated positions and refined using a riding mode. Multiple disordered solvent molecules were observed in the crystal structures of some complexes. Whenever possible, co-crystallizing solvent molecules were modeled. Otherwise, SQUEEZE was employed to treat diffuse solvent contribution in the voids. For complex **5** and **6**, Single crystals data were collected on Rigaku XtaLABHyPix-Arc 150 diffractometer with Cu-Kα radiation (λ = 1.54178 Å) at 100(10) K. The structure determinations and refinements were carried out using the SHELXS and SHELXL programs, respectively, on the Olex2 interface. The structures were solved using direct methods, which yielded the positions of all nonhydrogen atoms. Hydrogen atoms on carbons were placed in calculated positions in the final structure refinement.

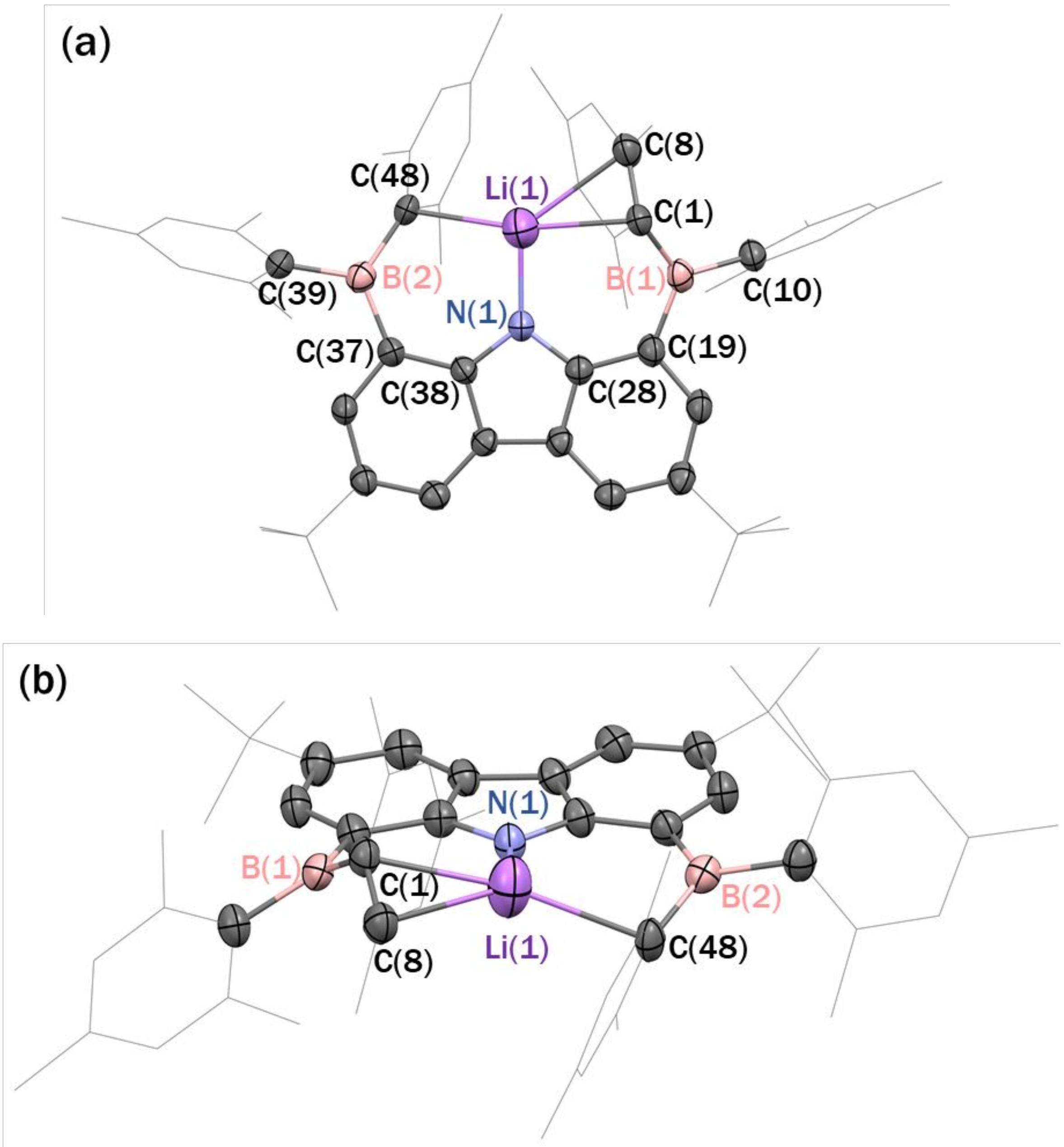

Figure S35. ORTEP of **LLi** (**1**). (a) The front view and (b) the top view of X-ray structure of complex **1**. 50% thermal ellipsoids; Hydrogen atoms, the disorder portions of the *t*-butyl and one of two independent molecules are omitted for clarity. Selected interatomic distances (Å) and angles (º): N(1)–Li(1) 1.900(9), C(1)···Li(1) 2.384(9), C(8)···Li(1) 2.538(9), C(48)···Li(1) 2.395(10).

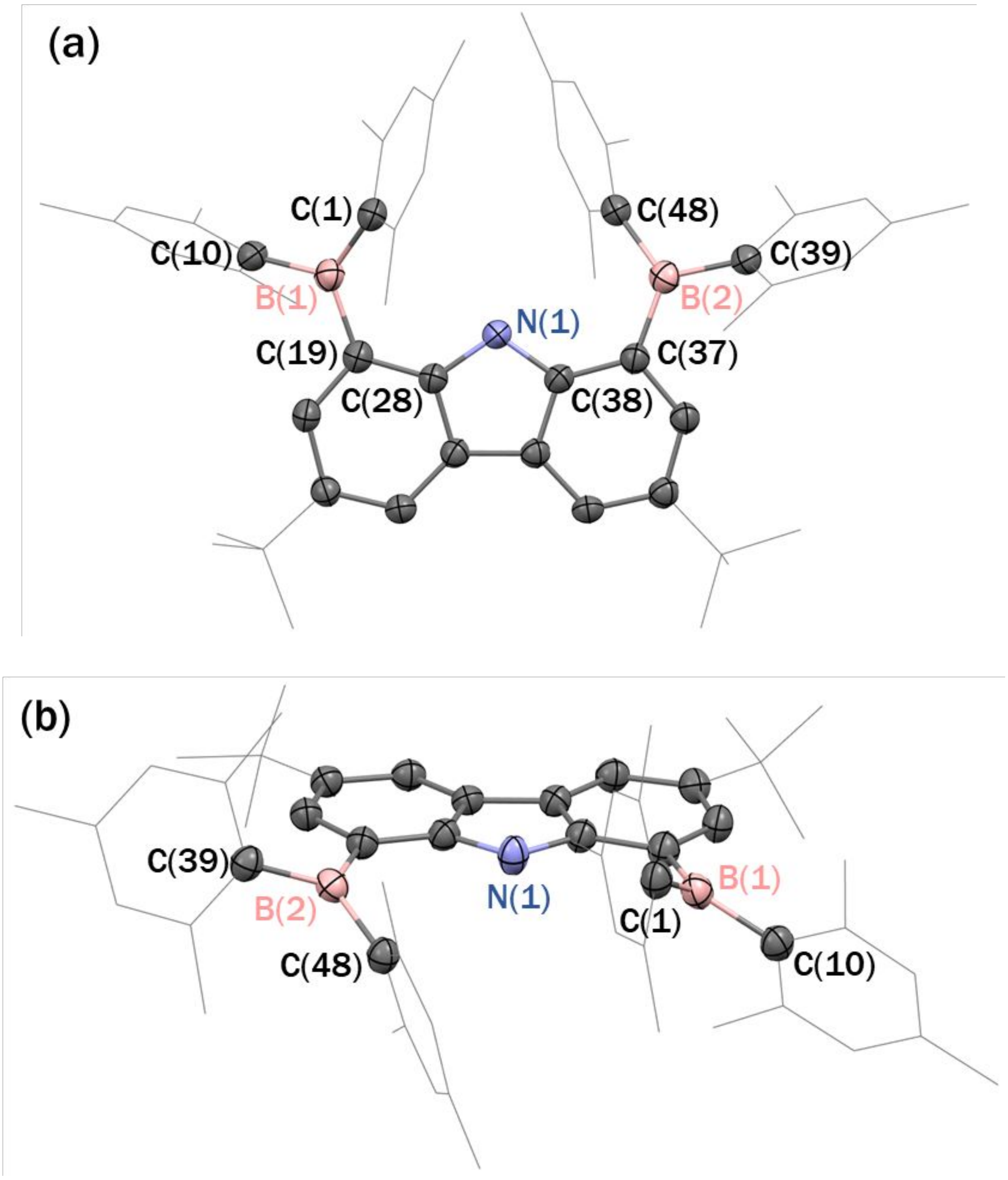

Figure S36. ORTEP of **LH** (**1-H**). (a) The front view and (b) the top view of X-ray structure of complex **1-H**. 50% thermal ellipsoids; Hydrogen atoms and the disorder portions of the t-butyl are omitted for clarity.

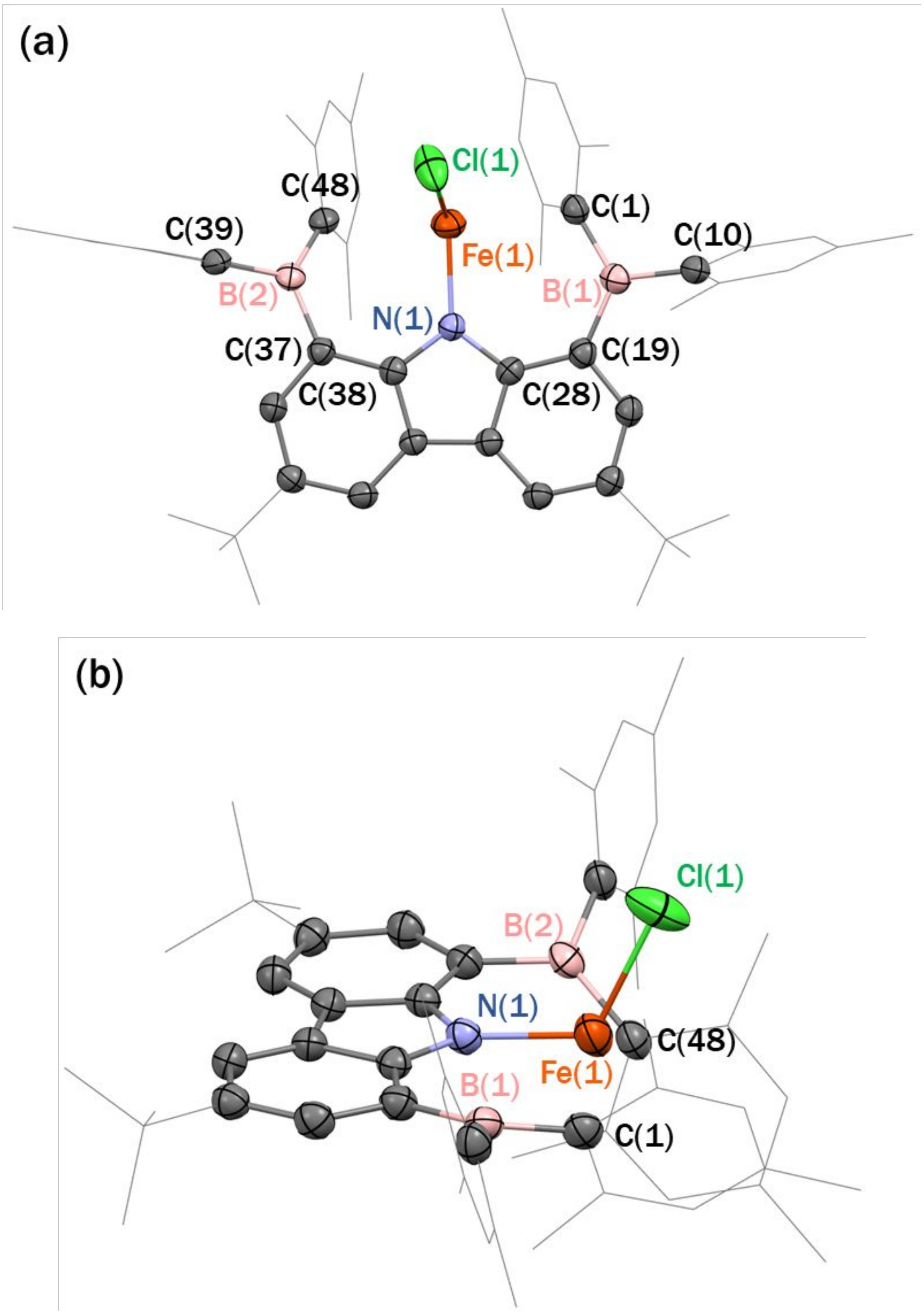

Figure S37. ORTEP of **LFeCl** (**2**). (a) The front view and (b) the side view of X-ray structure of complex **2**. 50% thermal ellipsoids; Hydrogen atoms are omitted for clarity. Selected interatomic distances (Å) and angles (º): N(1)–Fe(1) 1.960(2), Fe(1)–Cl(1) 2.2217(9), C(1)···Fe(1) 2.460(3), C(48)···Fe(1) 2.651(3); N(1)–Fe(1)–Cl(1) 119.37(7).

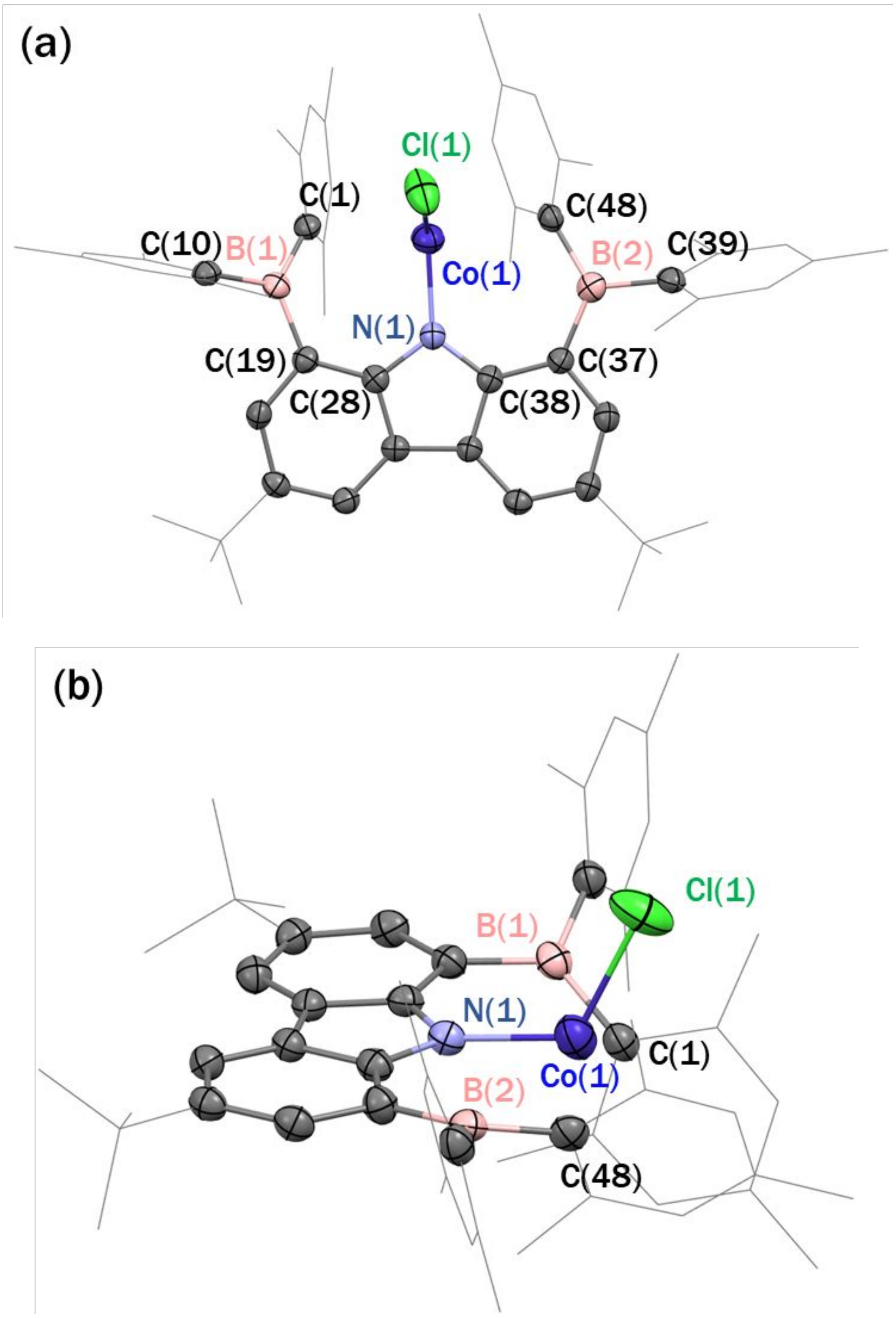

Figure S38. ORTEP of **LCoCl** (**3**). (a) The front view and (b) the side view of X-ray structure of complex **3**. 50% thermal ellipsoids; Hydrogen atoms are omitted for clarity. Selected interatomic distances (Å) and angles (º): N(1)–Co(1) 1.901(3), Co(1)–Cl(1) 2.2039(13), C(1)···Co(1) 2.536(5), C(48)···Co(1) 2.477(4); N(1)–Co(1)–Cl(1) 119.53(10).

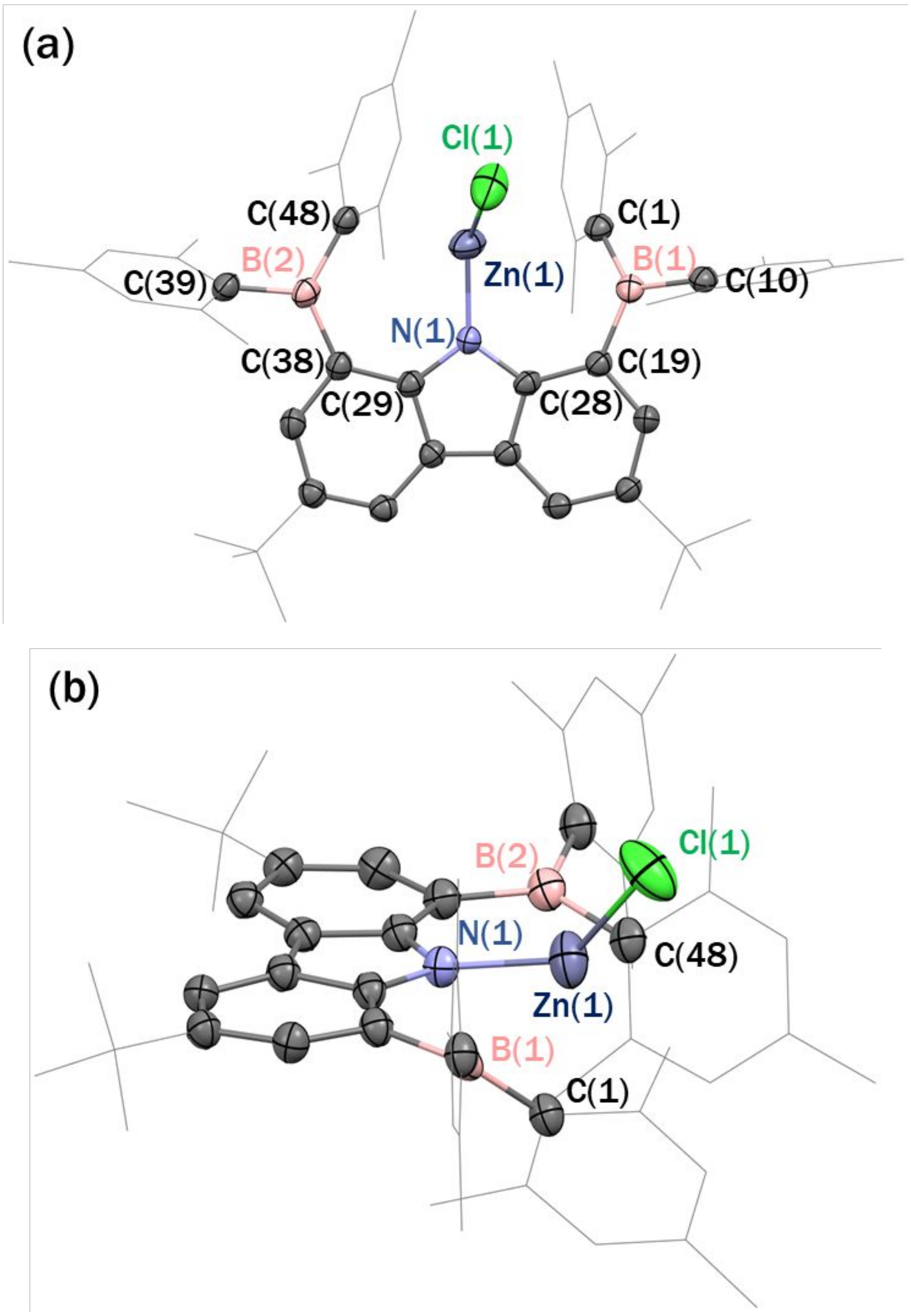

Figure S39. ORTEP of **LZnCl** (**4**). (a) The front view and (b) the side view of X-ray structure of complex **4**. 50% thermal ellipsoids; Hydrogen atoms are omitted for clarity. Selected interatomic distances (Å) and angles (º): N(1)–Zn(1) 1.892(3), Zn(1)–Cl(1) 2.1291(11), C(1)···Zn(1) 2.885(4), C(48)···Zn(1) 2.436(4); N(1)–Zn(1)–Cl(1) 133.49(9).

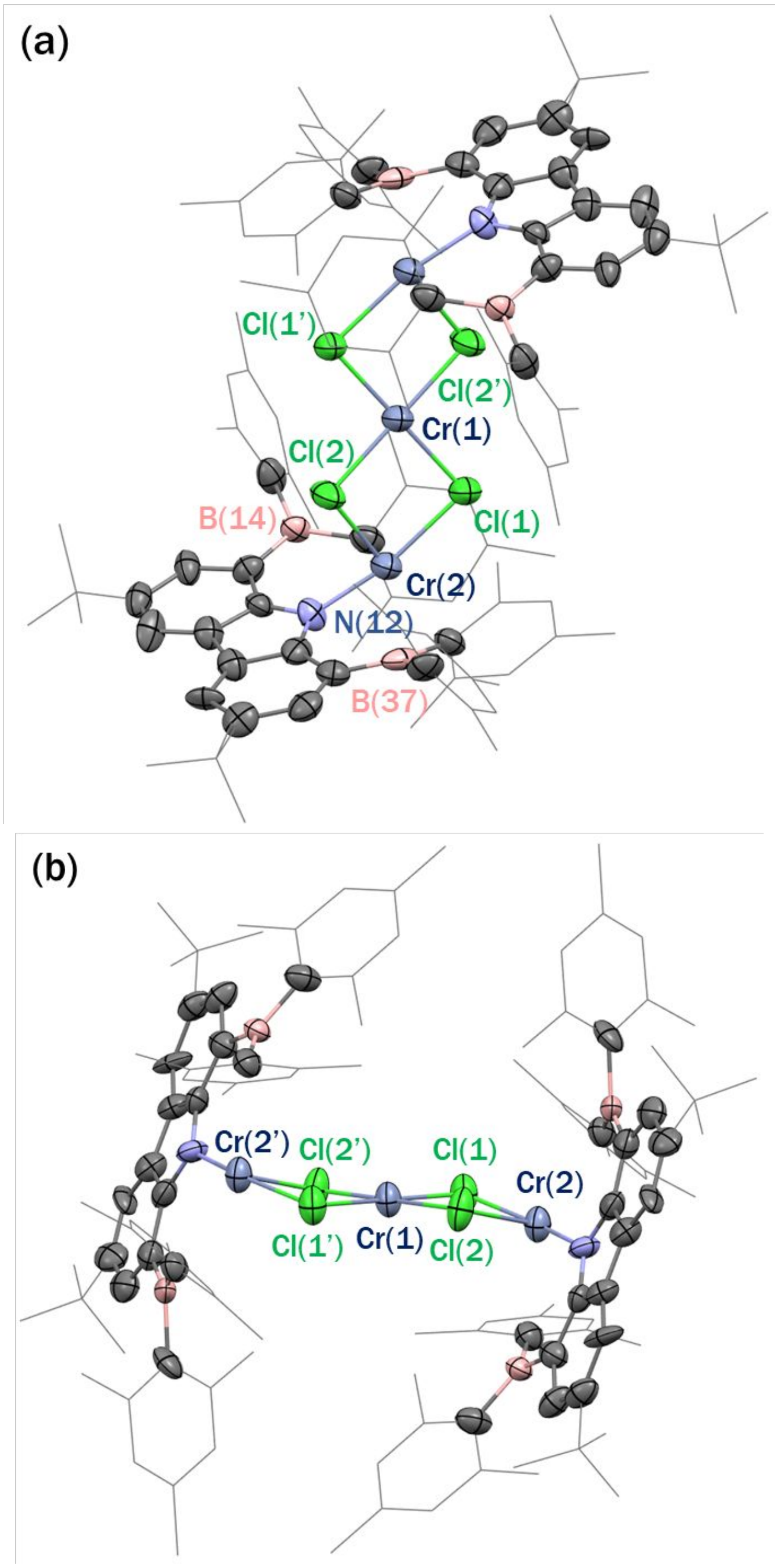

Figure S40. ORTEP of (**LCr**)$_2$(**CrCl**$_4$) (**5**). (a) The front view and (b) the side view of X-ray structure of complex **5**. 50% thermal ellipsoids.

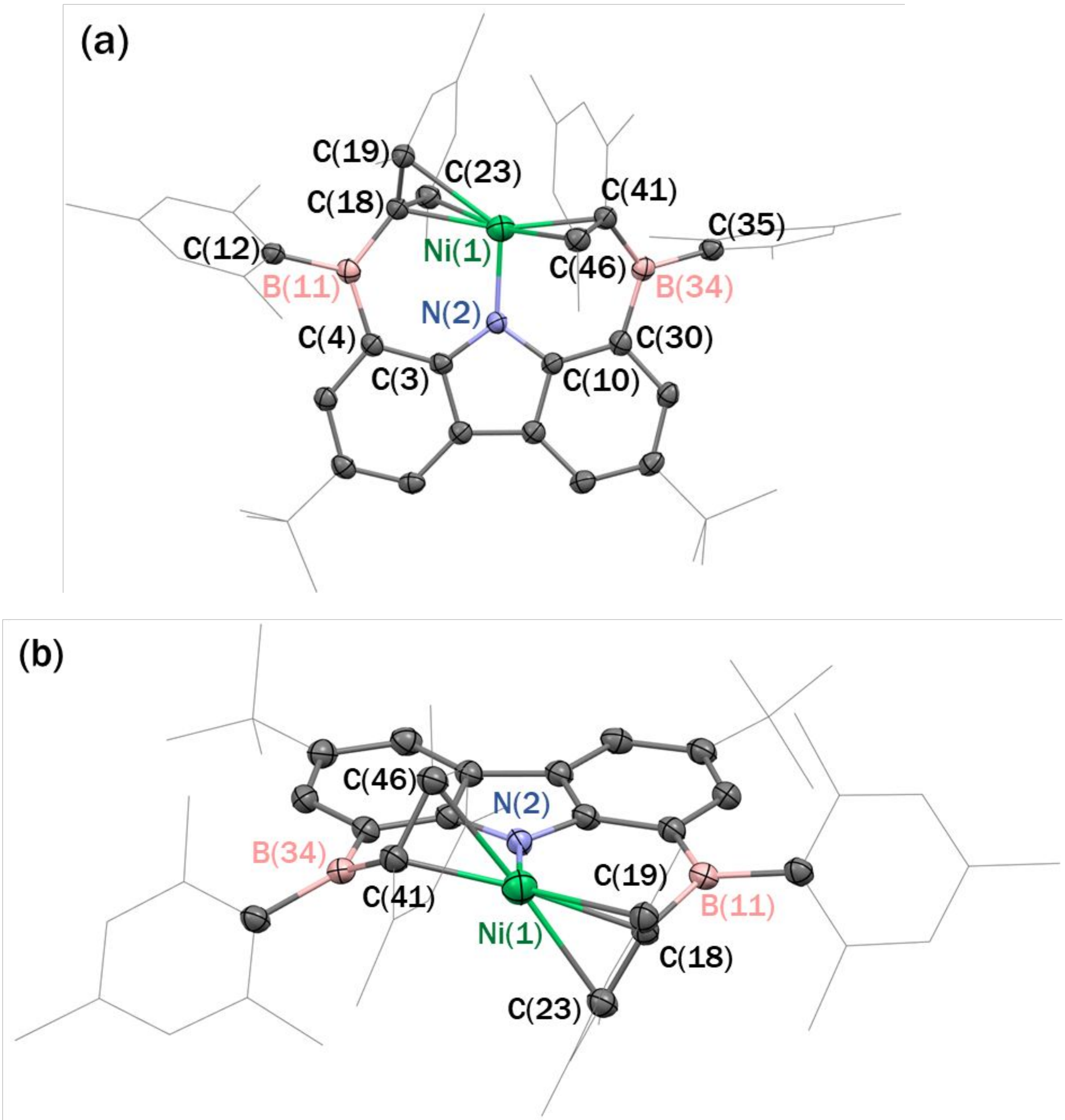

Figure S41. ORTEP of **LNi** (**6**). (a) The front view and (b) the top view of X-ray structure of complex **6**. 50% thermal ellipsoids; Hydrogen atoms are omitted for clarity. Selected interatomic distances (Å) and angles (º): N(2)–Ni(1) 1.914(3), C(18)···Ni(1) 2.141(3), C(19)···Ni(1) 2.458(3), C(23)···Ni(1) 2.350(3), C(41)···Ni(1) 2.171(3), C(46)···Ni(1) 2.408(3).

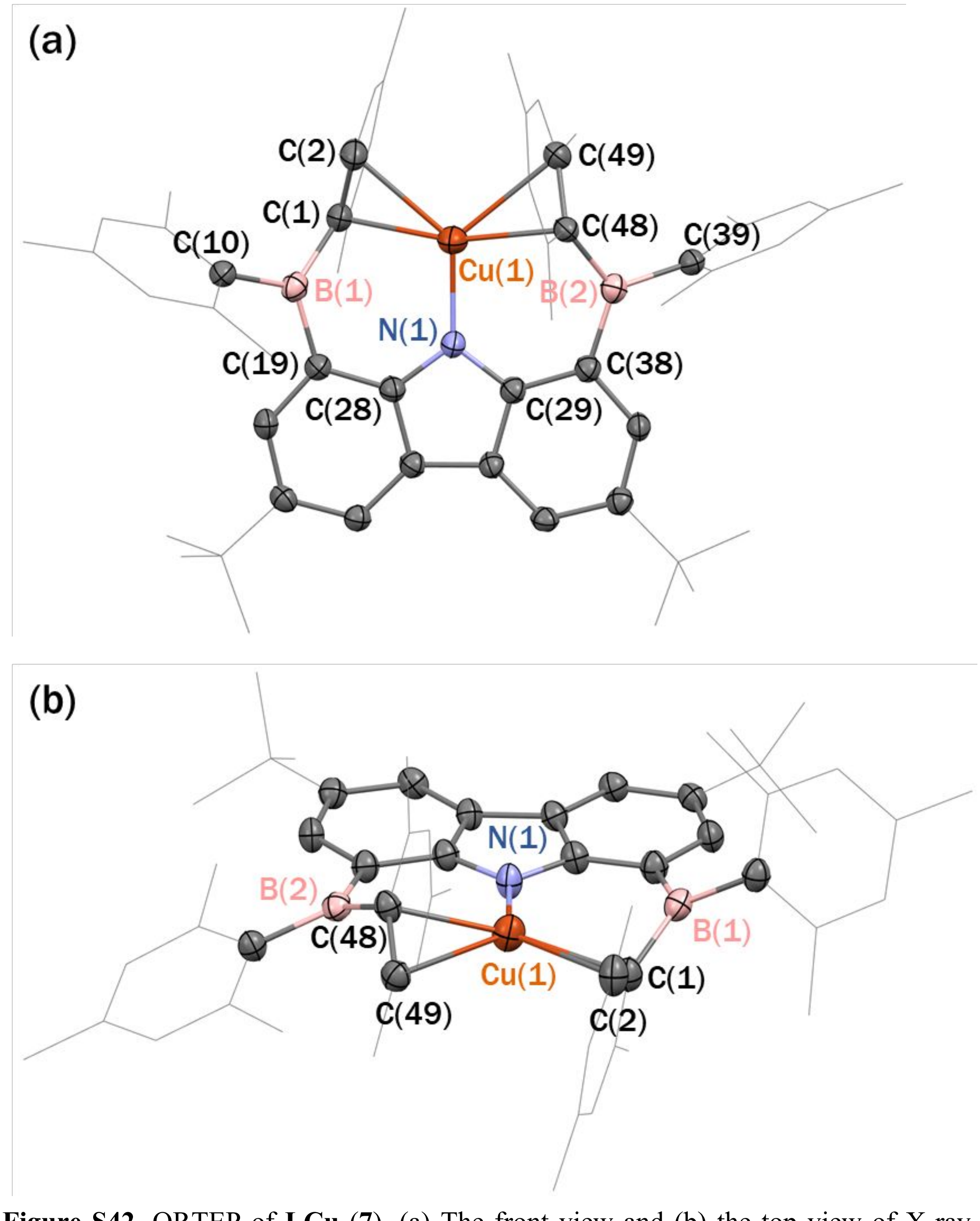

Figure S42. ORTEP of **LCu** (**7**). (a) The front view and (b) the top view of X-ray structure of complex **7**. 50% thermal ellipsoids; Hydrogen atoms and the disorder portions of the *t*-butyl are omitted for clarity. Selected interatomic distances (Å) and angles (º): N(1)–Cu(1) 1.918(2), C(1)···Cu(1) 2.159(3), C(2)···Cu(1) 2.396(3), C(48)···Cu(1) 2.169(3), C(49)···Cu(1) 2.452(3).

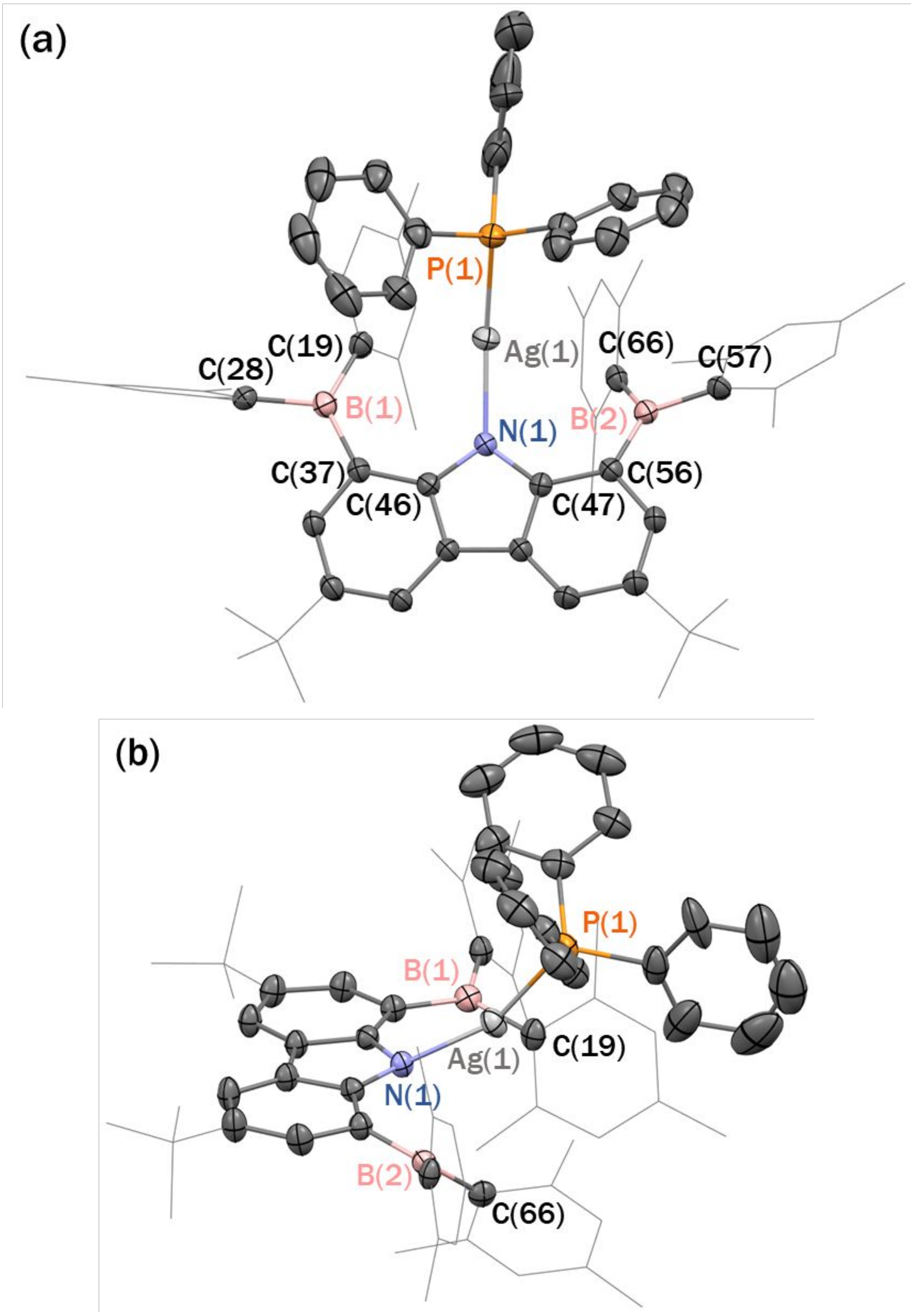

Figure S43. ORTEP of **LAgPPh$_3$** (**8**). (a) The front view and (b) the side view of X-ray structure of complex **8**. 50% thermal ellipsoids; Hydrogen atoms, solvate molecule and the disorder portions of the *t*-butyl are omitted for clarity. Selected interatomic distances (Å) and angles (º): N(1)–Ag(1) 2.122(2), P(1)–Ag(1) 2.3917(9), C(19)···Ag(1) 3.030(3), C(66)···Ag(1) 3.527(3), N(1)–Ag(1)–P(1) 157.73(7).

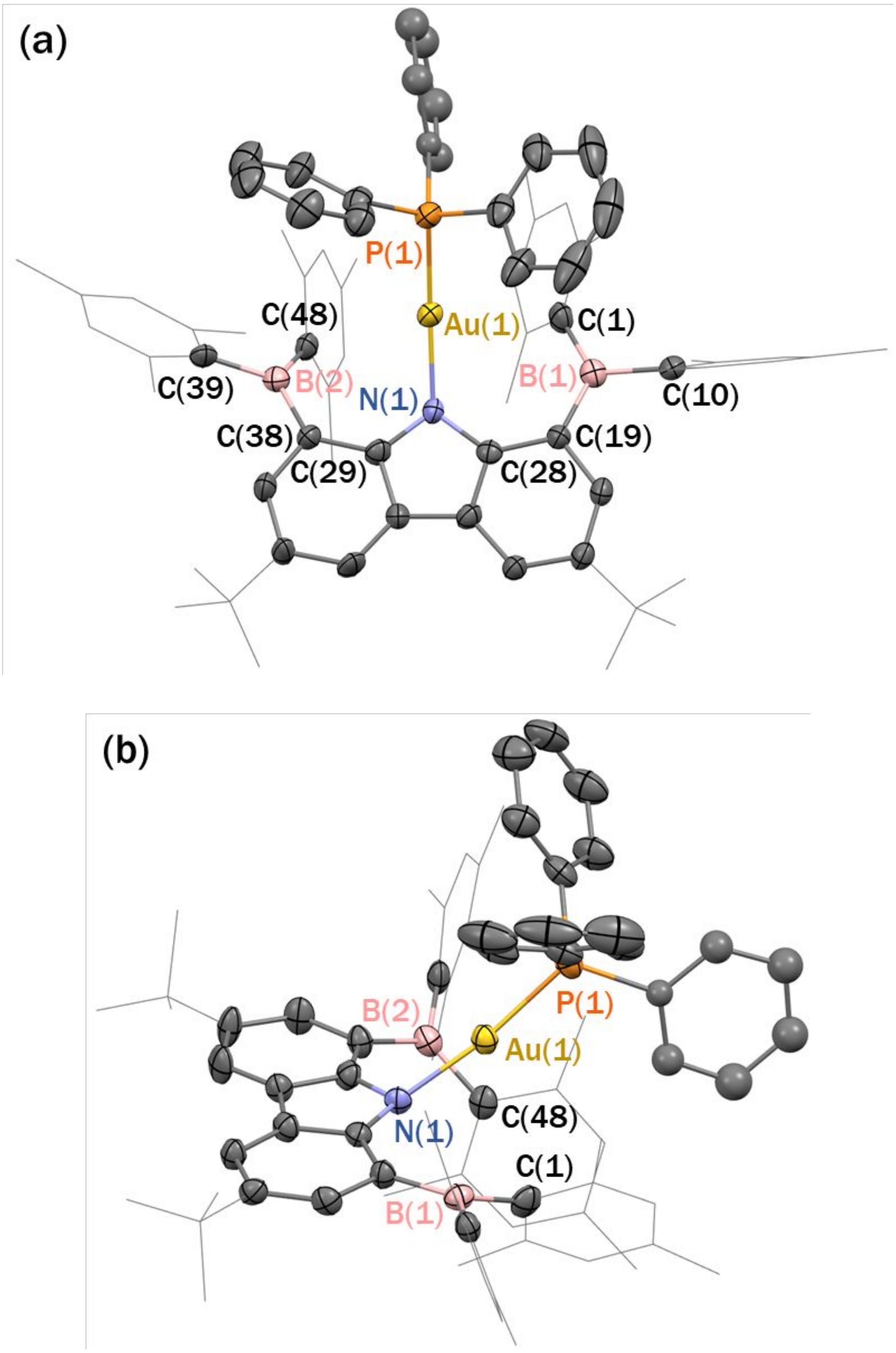

Figure S44. ORTEP of **LAuPPh$_3$** (**9**). (a) The front view and (b) the side view of X-ray structure of complex **9**. 50% thermal ellipsoids; Hydrogen atoms and the disorder portions of the *t*-butyl and phenyl groups are omitted for clarity. Selected interatomic distances (Å) and angles (º): N(1)–Au(1) 2.052(4), P(1)–Au(1) 2.2491(14), C(1)···Au(1) 3.387(5), C(48)···Au(1) 3.720(5), N(1)–Au(1)–P(1) 171.38(11).

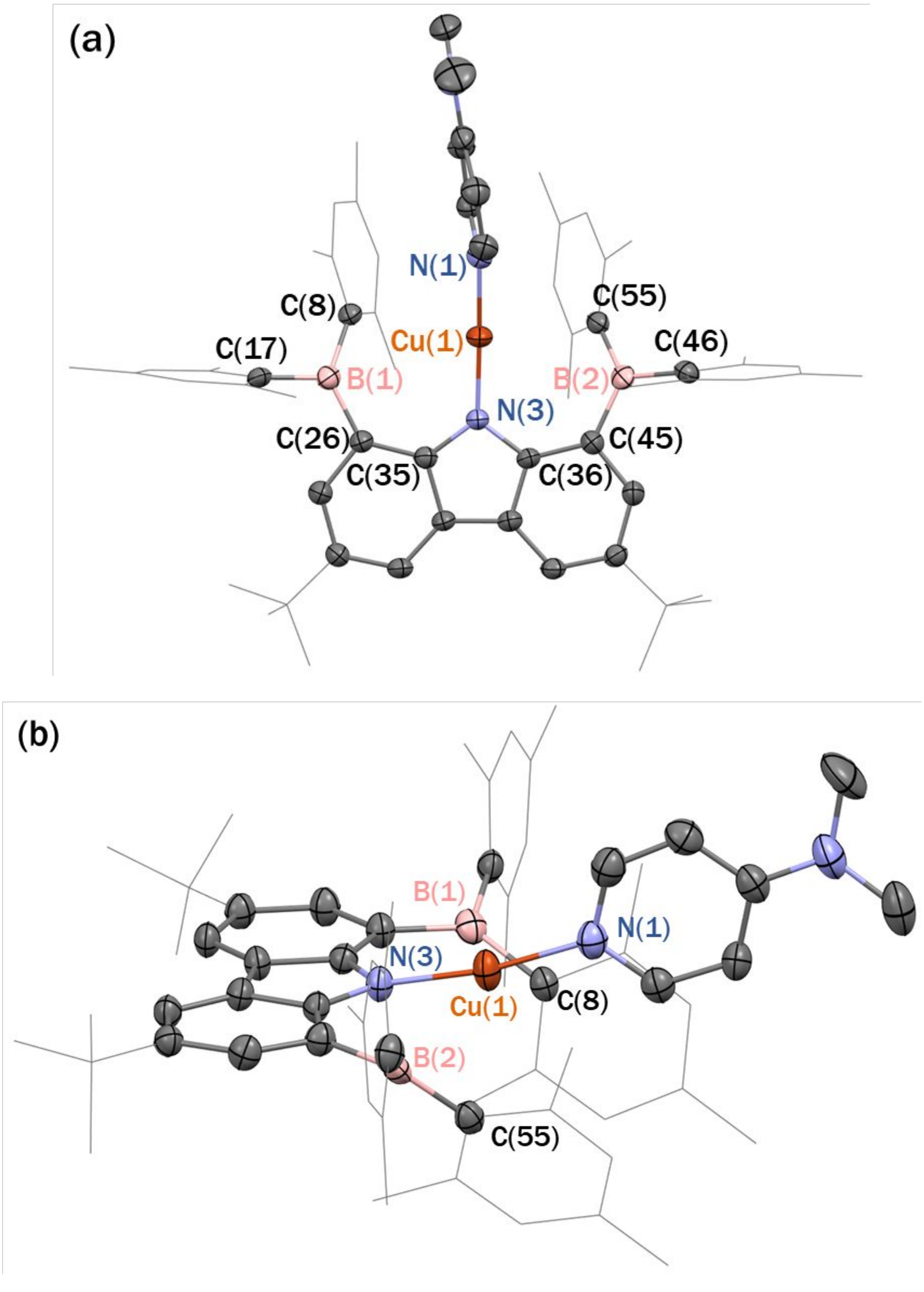

Figure S45. ORTEP of **LCu**(**DMAP**) (**10**). (a) The front view and (b) the side view of X-ray structure of complex **10**. 50% thermal ellipsoids; Hydrogen atoms and the disorder portions of the *t*-butyl are omitted for clarity. Selected interatomic distances (Å) and angles (º): N(3)–Cu(1) 1.854(3), N(1)–Cu(1) 1.881(3), C(8)···Cu(1) 3.057(3), C(55)···Cu(1) 3.063(3), N(3)–Cu(1)–N(1) 171.52(13).

Table S1. Crystal data and structure refinement for **LLi** (**1**).

Identification code	shelx	
Empirical formula	C112 H132 B4 Li2 N2 O0	
Formula weight	1563.31	
Temperature	200(2) K	
Wavelength	0.71073 Å	
Crystal system	Triclinic	
Space group	P -1	
Unit cell dimensions	a = 15.017(3) Å	α= 84.899(5)°.
	b = 18.464(3) Å	β= 77.337(5)°.
	c = 19.450(4) Å	γ = 86.540(5)°.
Volume	5236.4(17) $Å^3$	
Z	2	
Density (calculated)	0.991 Mg/m^3	
Absorption coefficient	0.055 mm^{-1}	
F(000)	1688	
Crystal size	0.500 x 0.440 x 0.020 mm^3	
Theta range for data collection	1.933 to 25.108°.	
Index ranges	-17<=h<=17, -21<=k<=22, -23<=l<=23	
Reflections collected	89731	
Independent reflections	18557 [R(int) = 0.1239]	
Completeness to theta = 25.108°	99.5 %	
Refinement method	Full-matrix least-squares on F^2	
Data / restraints / parameters	18557 / 6 / 1115	
Goodness-of-fit on F^2	1.031	
Final R indices [I>2sigma(I)]	R1 = 0.0829, wR2 = 0.2185	
R indices (all data)	R1 = 0.1677, wR2 = 0.2705	
Extinction coefficient	n/a	
Largest diff. peak and hole	0.558 and -0.375 e.$Å^{-3}$	

Table S2. Crystal data and structure refinement for **LH** (**1-H**).

Identification code	shelx	
Empirical formula	C56 H67 B2 N	
Formula weight	775.72	
Temperature	200(2) K	
Wavelength	0.71073 Å	
Crystal system	Monoclinic	
Space group	P 21/n	
Unit cell dimensions	a = 13.6488(4) Å	α= 90°.
	b = 25.4868(7) Å	β= 115.1470(10)°.
	c = 15.3942(5) Å	γ = 90°.
Volume	4847.5(3) $Å^3$	
Z	4	
Density (calculated)	1.063 Mg/m^3	
Absorption coefficient	0.059 mm^{-1}	
F(000)	1680	
Crystal size	0.520 x 0.270 x 0.210 mm^3	
Theta range for data collection	2.296 to 25.044°.	
Index ranges	-16<=h<=16, -30<=k<=30, -18<=l<=18	
Reflections collected	97050	
Independent reflections	8581 [R(int) = 0.0552]	
Completeness to theta = 25.044°	99.9 %	
Absorption correction	Semi-empirical from equivalents	
Max. and min. transmission	0.988 and 0.970	
Refinement method	Full-matrix least-squares on F^2	
Data / restraints / parameters	8581 / 36 / 574	
Goodness-of-fit on F^2	1.034	
Final R indices [I>2sigma(I)]	R1 = 0.0517, wR2 = 0.1259	
R indices (all data)	R1 = 0.0734, wR2 = 0.1409	
Extinction coefficient	n/a	
Largest diff. peak and hole	0.379 and -0.222 e.$Å^{-3}$	

Table S3. Crystal data and structure refinement for **LFeCl** (**2**).

Identification code	shelx	
Empirical formula	C56 H66 B2 Cl Fe N	
Formula weight	866.01	
Temperature	200(2) K	
Wavelength	0.71073 Å	
Crystal system	Triclinic	
Space group	P -1	
Unit cell dimensions	a = 13.0435(6) Å	α= 112.2810(10)°.
	b = 14.3430(6) Å	β= 106.217(2)°.
	c = 14.8199(7) Å	γ = 91.7290(10)°.
Volume	2434.40(19) $Å^3$	
Z	2	
Density (calculated)	1.181 Mg/m^3	
Absorption coefficient	0.401 mm^{-1}	
F(000)	924	
Crystal size	0.450 x 0.260 x 0.070 mm^3	
Theta range for data collection	2.082 to 25.092°.	
Index ranges	-15<=h<=15, -17<=k<=17, -17<=l<=17	
Reflections collected	69922	
Independent reflections	8619 [R(int) = 0.0638]	
Completeness to theta = 25.092°	99.5 %	
Refinement method	Full-matrix least-squares on F^2	
Data / restraints / parameters	8619 / 0 / 568	
Goodness-of-fit on F^2	1.037	
Final R indices [I>2sigma(I)]	R1 = 0.0482, wR2 = 0.1230	
R indices (all data)	R1 = 0.0772, wR2 = 0.1418	
Extinction coefficient	n/a	
Largest diff. peak and hole	0.283 and -0.601 e.$Å^{-3}$	

Table S4. Crystal data and structure refinement for **LCoCl** (**3**).

Identification code	shelx	
Empirical formula	C56 H66 B2 Cl Co N	
Formula weight	869.09	
Temperature	200(2) K	
Wavelength	0.71073 Å	
Crystal system	Triclinic	
Space group	P -1	
Unit cell dimensions	a = 12.9879(11) Å	α= 112.163(2)°.
	b = 14.3287(11) Å	β= 106.098(2)°.
	c = 14.8079(12) Å	γ = 91.763(2)°.
Volume	2423.5(3) $Å^3$	
Z	2	
Density (calculated)	1.191 Mg/m^3	
Absorption coefficient	0.446 mm^{-1}	
F(000)	926	
Crystal size	0.130 x 0.120 x 0.020 mm^3	
Theta range for data collection	2.087 to 25.025°.	
Index ranges	-15<=h<=15, -17<=k<=17, -17<=l<=17	
Reflections collected	69561	
Independent reflections	8544 [R(int) = 0.1054]	
Completeness to theta = 25.025°	99.8 %	
Refinement method	Full-matrix least-squares on F^2	
Data / restraints / parameters	8544 / 0 / 568	
Goodness-of-fit on F^2	1.045	
Final R indices [I>2sigma(I)]	R1 = 0.0602, wR2 = 0.1501	
R indices (all data)	R1 = 0.1181, wR2 = 0.1862	
Extinction coefficient	n/a	
Largest diff. peak and hole	0.338 and -0.790 e.$Å^{-3}$	

Table S5. Crystal data and structure refinement for **LZnCl** (**4**).

Identification code	shelx	
Empirical formula	C56 H66 B2 Cl N Zn	
Formula weight	875.53	
Temperature	200(2) K	
Wavelength	0.71073 Å	
Crystal system	Triclinic	
Space group	P -1	
Unit cell dimensions	a = 13.0822(6) Å	α= 112.1720(10)°.
	b = 14.4074(7) Å	β= 106.1800(10)°.
	c = 14.7682(6) Å	γ = 91.5290(10)°.
Volume	2448.24(19) $Å^3$	
Z	2	
Density (calculated)	1.188 Mg/m^3	
Absorption coefficient	0.593 mm^{-1}	
F(000)	932	
Crystal size	0.140 x 0.130 x 0.010 mm^3	
Theta range for data collection	2.078 to 25.138°.	
Index ranges	-15<=h<=15, -17<=k<=17, -17<=l<=17	
Reflections collected	89879	
Independent reflections	8720 [R(int) = 0.0762]	
Completeness to theta = 25.138°	99.4 %	
Absorption correction	Semi-empirical from equivalents	
Max. and min. transmission	0.994 and 0.922	
Refinement method	Full-matrix least-squares on F^2	
Data / restraints / parameters	8720 / 1 / 568	
Goodness-of-fit on F^2	1.062	
Final R indices [I>2sigma(I)]	R1 = 0.0577, wR2 = 0.1572	
R indices (all data)	R1 = 0.0843, wR2 = 0.1778	
Extinction coefficient	n/a	
Largest diff. peak and hole	0.413 and -0.853 e.$Å^{-3}$	

Table S6. Crystal data and structure refinement for **LNi** (**6**).

Identification code	230925lt2_auto
Empirical formula	$C_{56}H_{66}B_2NNi$
Formula weight	833.42
Temperature/K	99.99(10)
Crystal system	triclinic
Space group	P-1
a/Å	14.2993(5)
b/Å	14.3233(3)
c/Å	14.5520(3)
α/°	76.0697(19)
β/°	61.009(3)
γ/°	68.423(2)
Volume/Å^3	2417.12(13)
Z	2
ρ_{calc}g/cm^3	1.145
μ/mm^{-1}	0.835
F(000)	894.0
Crystal size/mm^3	0.17 × 0.09 × 0.02
Radiation	Cu Kα (λ = 1.54184)
2Θ range for data collection/°	6.656 to 150.56
Index ranges	-17 ≤ h ≤ 17, -13 ≤ k ≤ 17, -17 ≤ l ≤ 18
Reflections collected	30788
Independent reflections	9461 [R_{int} = 0.0259, R_{sigma} = 0.0315]
Data/restraints/parameters	9461/0/559
Goodness-of-fit on F^2	1.060
Final R indexes [I>=2σ (I)]	R_1 = 0.0724, wR_2 = 0.2243
Final R indexes [all data]	R_1 = 0.0860, wR_2 = 0.2360
Largest diff. peak/hole / e Å^{-3}	0.79/-1.47

Table S7. Crystal data and structure refinement for **LCu** (**7**).

Identification code	shelx	
Empirical formula	C56 H66 B2 Cu N	
Formula weight	838.25	
Temperature	200(2) K	
Wavelength	0.71073 Å	
Crystal system	Monoclinic	
Space group	P 21/n	
Unit cell dimensions	a = 13.4517(10) Å	α= 90°.
	b = 25.5527(18) Å	β= 114.693(2)°.
	c = 15.3803(13) Å	γ = 90°.
Volume	4803.2(6) $Å^3$	
Z	4	
Density (calculated)	1.159 Mg/m^3	
Absorption coefficient	0.491 mm^{-1}	
F(000)	1792	
Crystal size	0.400 x 0.270 x 0.040 mm^3	
Theta range for data collection	2.306 to 25.117°.	
Index ranges	-16<=h<=16, -30<=k<=30, -18<=l<=18	
Reflections collected	67164	
Independent reflections	8532 [R(int) = 0.0813]	
Completeness to theta = 25.117°	99.5 %	
Refinement method	Full-matrix least-squares on F^2	
Data / restraints / parameters	8532 / 13 / 584	
Goodness-of-fit on F^2	1.050	
Final R indices [I>2sigma(I)]	R1 = 0.0507, wR2 = 0.1278	
R indices (all data)	R1 = 0.0866, wR2 = 0.1498	
Extinction coefficient	n/a	
Largest diff. peak and hole	0.307 and -0.792 e.$Å^{-3}$	

Table S8. Crystal data and structure refinement for **$LAgPPh_3$ (8)**.

Identification code	shelx	
Empirical formula	C78 H89 Ag B2 N O P	
Formula weight	1216.96	
Temperature	200(2) K	
Wavelength	0.71073 Å	
Crystal system	Monoclinic	
Space group	C 2/c	
Unit cell dimensions	a = 27.859(2) Å	α= 90°.
	b = 12.1594(10) Å	β= 96.312(2)°.
	c = 42.642(3) Å	γ = 90°.
Volume	14357.5(19) Å^3	
Z	8	
Density (calculated)	1.126 Mg/m^3	
Absorption coefficient	0.345 mm^{-1}	
F(000)	5152	
Crystal size	0.570 x 0.400 x 0.220 mm^3	
Theta range for data collection	1.871 to 25.080°.	
Index ranges	-33<=h<=30, -14<=k<=14, -49<=l<=50	
Reflections collected	70021	
Independent reflections	12717 [R(int) = 0.0467]	
Completeness to theta = 25.080°	99.5 %	
Refinement method	Full-matrix least-squares on F^2	
Data / restraints / parameters	12717 / 64 / 766	
Goodness-of-fit on F^2	1.021	
Final R indices [I>2sigma(I)]	R1 = 0.0524, wR2 = 0.1345	
R indices (all data)	R1 = 0.0605, wR2 = 0.1411	
Extinction coefficient	n/a	
Largest diff. peak and hole	1.391 and -1.736 e.Å^{-3}	

Table S9. Crystal data and structure refinement for **$LAuPPh_3$ (9)**.

Identification code	shelx	
Empirical formula	C74 H81 Au B2 N P	
Formula weight	1233.95	
Temperature	200(2) K	
Wavelength	0.71073 Å	
Crystal system	Monoclinic	
Space group	C 2/c	
Unit cell dimensions	a = 27.7183(11) Å	α= 90°.
	b = 12.2773(4) Å	β= 96.6430(10)°.
	c = 42.6591(12) Å	γ = 90°.
Volume	14419.7(8) $Å^3$	
Z	8	
Density (calculated)	1.137 Mg/m^3	
Absorption coefficient	2.100 mm^{-1}	
F(000)	5088	
Crystal size	0.150 x 0.070 x 0.030 mm^3	
Theta range for data collection	2.095 to 25.076°.	
Index ranges	-26<=h<=32, -14<=k<=14, -50<=l<=50	
Reflections collected	53670	
Independent reflections	12749 [R(int) = 0.0682]	
Completeness to theta = 25.076°	99.5 %	
Refinement method	Full-matrix least-squares on F^2	
Data / restraints / parameters	12749 / 11 / 707	
Goodness-of-fit on F^2	1.013	
Final R indices [I>2sigma(I)]	R1 = 0.0411, wR2 = 0.0880	
R indices (all data)	R1 = 0.0665, wR2 = 0.0981	
Extinction coefficient	n/a	
Largest diff. peak and hole	1.701 and -1.364 e.$Å^{-3}$	

Table S10. Crystal data and structure refinement for **LCu(DMAP)** (**10**).

Identification code	shelx	
Empirical formula	C63 H76 B2 Cu N3	
Formula weight	960.42	
Temperature	200(2) K	
Wavelength	0.71073 Å	
Crystal system	Monoclinic	
Space group	P 21/n	
Unit cell dimensions	a = 16.111(9) Å	α= 90°.
	b = 16.874(7) Å	β= 94.676(17)°.
	c = 20.687(9) Å	γ = 90°.
Volume	5605(5) Å^3	
Z	4	
Density (calculated)	1.138 Mg/m^3	
Absorption coefficient	0.430 mm^{-1}	
F(000)	2056	
Crystal size	0.200 x 0.060 x 0.030 mm^3	
Theta range for data collection	1.959 to 25.041°.	
Index ranges	-15<=h<=19, -20<=k<=20, -24<=l<=24	
Reflections collected	59804	
Independent reflections	9892 [R(int) = 0.1299]	
Completeness to theta = 25.041°	99.8 %	
Absorption correction	Semi-empirical from equivalents	
Max. and min. transmission	0.987 and 0.919	
Refinement method	Full-matrix least-squares on F^2	
Data / restraints / parameters	9892 / 38 / 667	
Goodness-of-fit on F^2	1.024	
Final R indices [I>2sigma(I)]	R1 = 0.0531, wR2 = 0.1049	
R indices (all data)	R1 = 0.1271, wR2 = 0.1355	
Extinction coefficient	n/a	
Largest diff. peak and hole	0.264 and -0.385 e.Å^{-3}	

4. Buried Volume Analysis and Steric Maps

General Considerations

The buried volume of all the complexes were determined via the SambVca 2.1 webtool.[1] The buried volume values (%V_{bur}) and the topographic steric maps were taken directly from the generated output from the webtool in this report. The data was processed with the following details:

• The input file was taken directly from the single-crystal X-ray diffraction data.

• The metal atom was chosen as the origin (center) of each buried volume analysis and the topographic steric maps, with all atoms except the principal ligand removed from the model.

• The Z-axis was defined along the vector from the nitrogen atom of the carbazole ring to the metal center, with the XZ-plane corresponding to the plane of the carbazole ring.

• Bondi radii were chosen at their unscaled (x1) values.

• The mesh spacing used was at the standard value of 0.10 Å.

• The radius of the sphere around the metal center was held at a standard value of 3.5 Å.

• Hydrogen atoms were not included in all calculations.

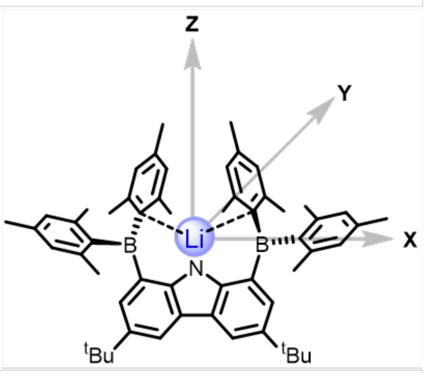

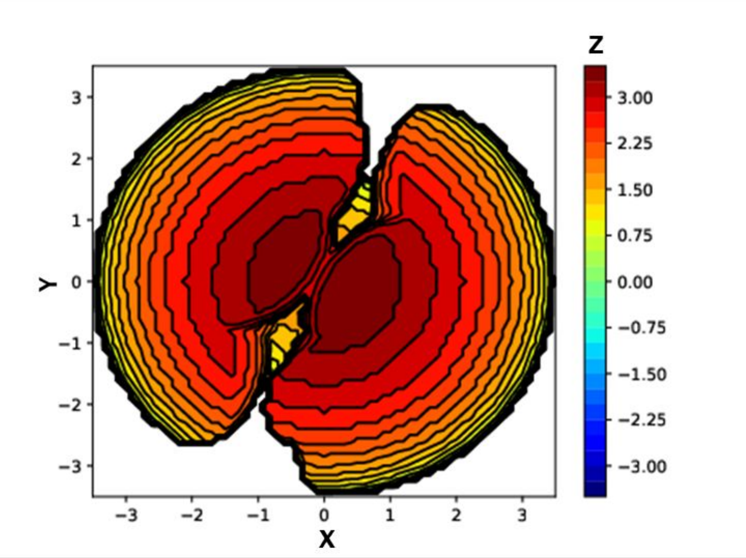

%V Free	%V Buried	% V Tot/V Ex
18.6	**81.4**	99.9

Quadrant	V f	V b	V t	%V f	%V b
SW	15.3	29.5	44.9	**34.2**	65.8
NW	1.7	43.2	44.9	**3.8**	96.2
NE	13.3	31.5	44.9	**29.7**	70.3
SE	3.0	41.8	44.9	**6.8**	93.2

Figure S46. Buried volume analysis and steric map of **LLi** (**1**).

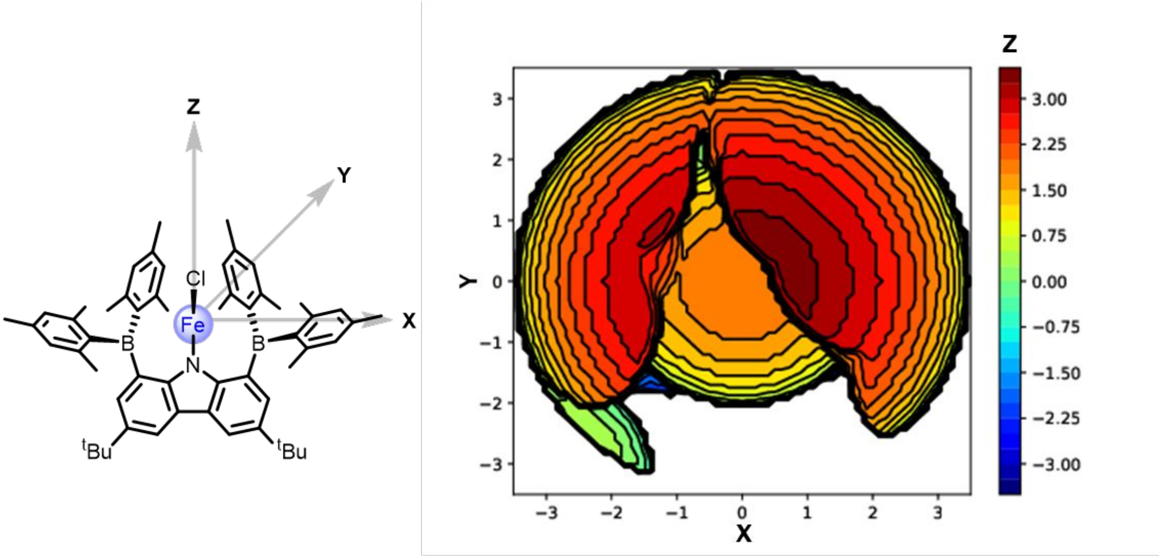

%V Free	**%V Buried**	% V Tot/V Ex
25.2	**74.8**	99.9

Quadrant	V f	V b	V t	%V f	**%V b**
SW	17.9	26.9	44.9	**40.0**	60.0
NW	6.4	38.4	44.9	**14.4**	85.6
NE	1.5	43.4	44.9	**3.2**	96.8
SE	19.4	25.5	44.9	**43.2**	56.8

Figure S47. Buried volume analysis and steric map of **LFeCl** (**2**).

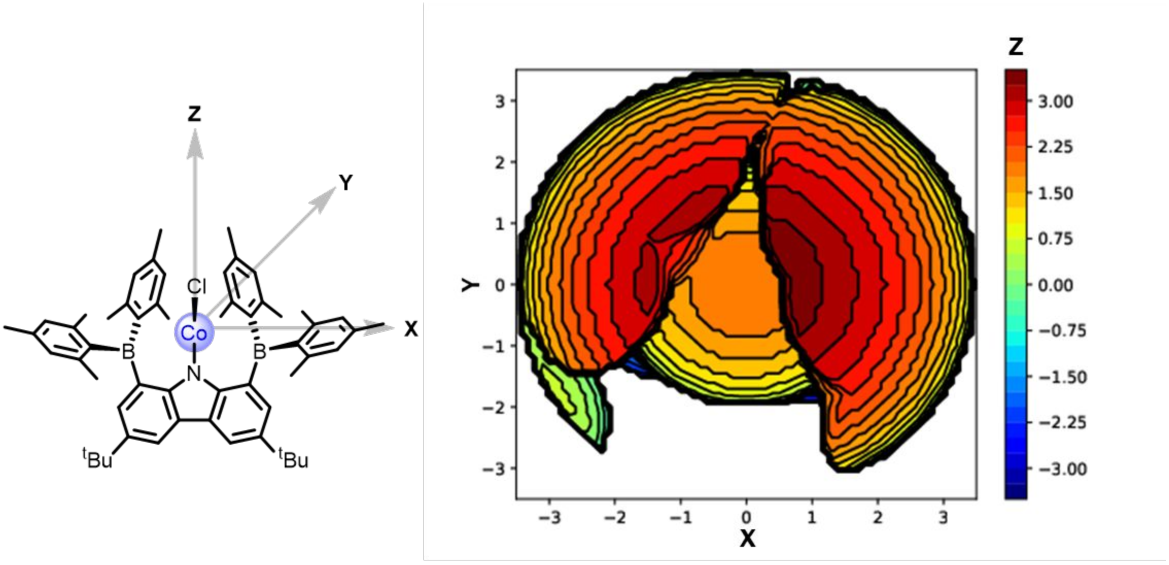

%V Free	%V Buried	% V Tot/V Ex
24.9	**75.1**	99.9

Quadrant	V f	V b	V t	%V f	%V b
SW	22.9	22.0	44.9	**51.0**	49.0
NW	2.9	42.0	44.9	**6.4**	93.6
NE	5.2	39.7	44.9	**11.5**	88.5
SE	13.8	31.1	44.9	**30.8**	69.2

Figure S48. Buried volume analysis and steric map of **LCoCl** (**3**).

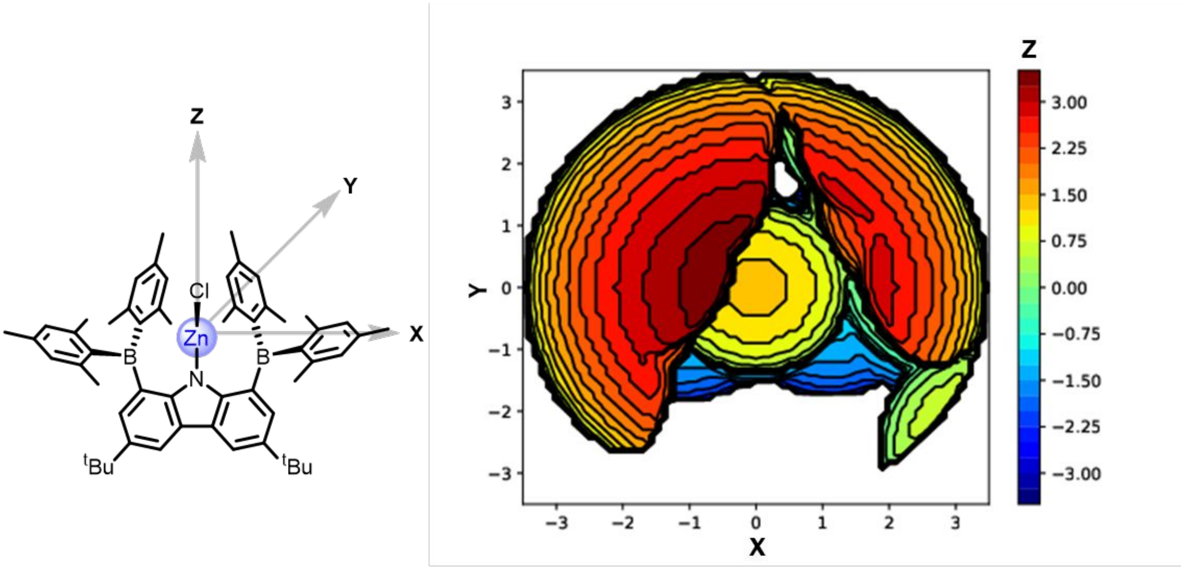

%V Free	%V Buried	% V Tot/V Ex
33.9	**66.1**	99.9

Quadrant	V f	V b	V t	%V f	%V b
SW	20.3	24.5	44.9	**45.3**	54.7
NW	3.7	41.2	44.9	**8.2**	91.8
NE	9.9	34.9	44.9	**22.1**	77.9
SE	26.9	18.0	44.9	**59.9**	40.1

Figure S49. Buried volume analysis and steric map of **LZnCl** (**4**).

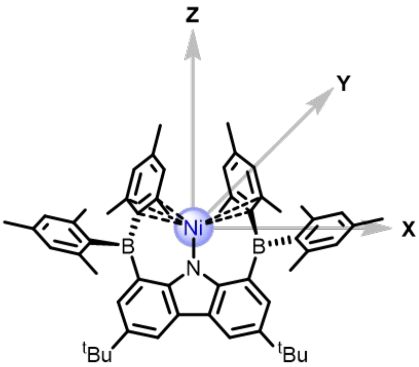

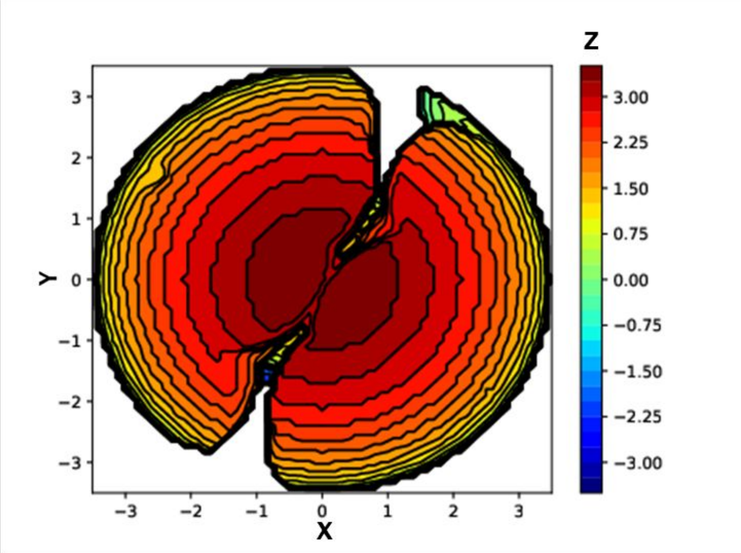

%V Free	**%V Buried**	% V Tot/V Ex
15.3	**84.7**	99.9

Quadrant	V f	V b	V t	%V f	**%V b**
SW	13.2	31.6	44.9	**29.5**	70.5
NW	0.9	44.0	44.9	**1.9**	98.1
NE	12.6	32.2	44.9	**28.2**	71.8
SE	0.8	44.1	44.9	**1.7**	98.3

Figure S50. Buried volume analysis and steric map of **LNi** (**6**).

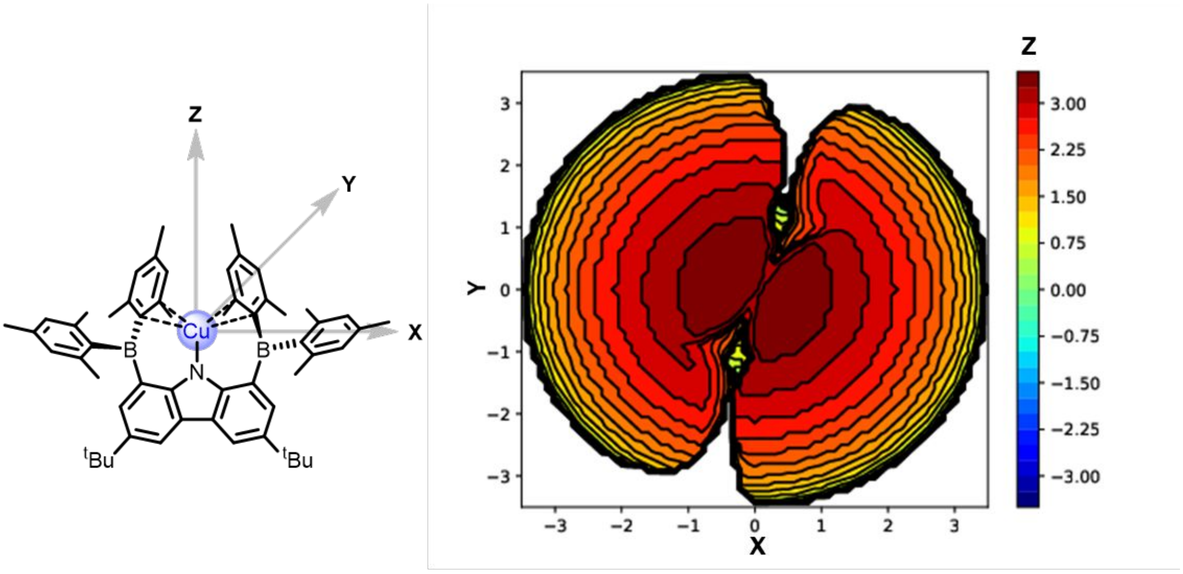

%V Free	%V Buried	% V Tot/V Ex
19.7	**80.3**	99.9

Quadrant	V f	V b	V t	%V f	%V b
SW	13.7	31.2	44.9	**30.5**	69.5
NW	3.8	41.0	44.9	**8.5**	91.5
NE	15.2	29.7	44.9	**33.9**	66.1
SE	2.6	42.3	44.9	**5.8**	94.2

Figure S51. Buried volume analysis and steric map of **LCu** (**7**).

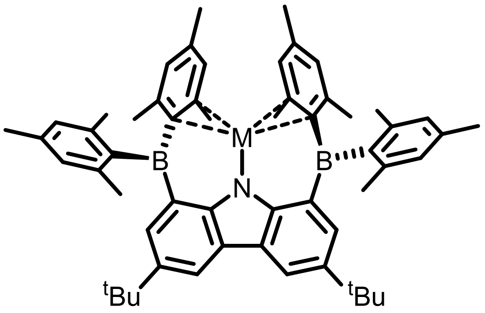

LM

M = Li (1), Ni (6), Cu (7)

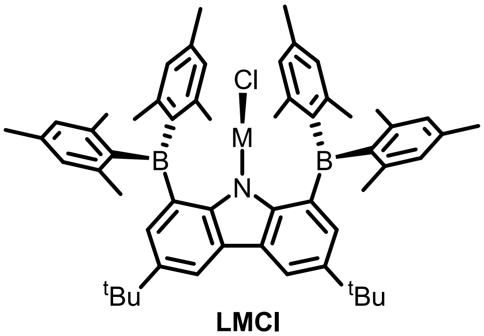

LMCl

M = Fe (2), Co (3), Zn (4)

Complex	Buried volume (%V_{bur})
1 (Li)	81.4
6 (Ni)	84.7
7 (Cu)	80.3
2 (Fe)	74.8
3 (Co)	75.1
4 (Zn)	66.1

Table S11. Synthesized complex structures and buried volume table.

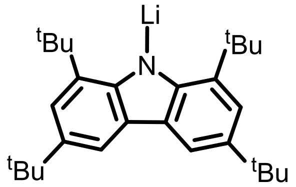

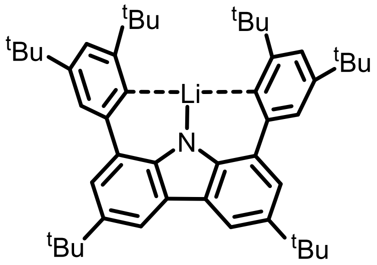

^{tBu4}CBzLi **^{dtbp}CBzLi**

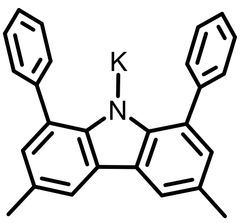

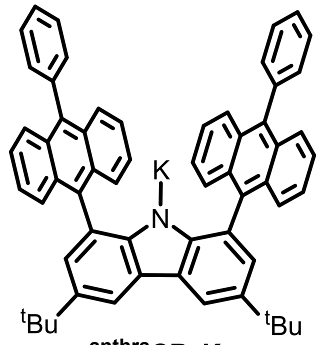

^{Ph}CBzK **^{anthra}CBzK**

Complex	Buried volume (%V_{bur})
^{tBu4}CBzLi	**45.9**
^{dtbp}CBzLi	**72.9**
^{Ph}CBzK	**68.7**
^{anthra}CBzK	**74.0**

Table S12. Reported alkali carbazolide complex structures and buried volume table; The input file was obtained directly from the single-crystal X-ray diffraction data reported in the original publication.[2–5]

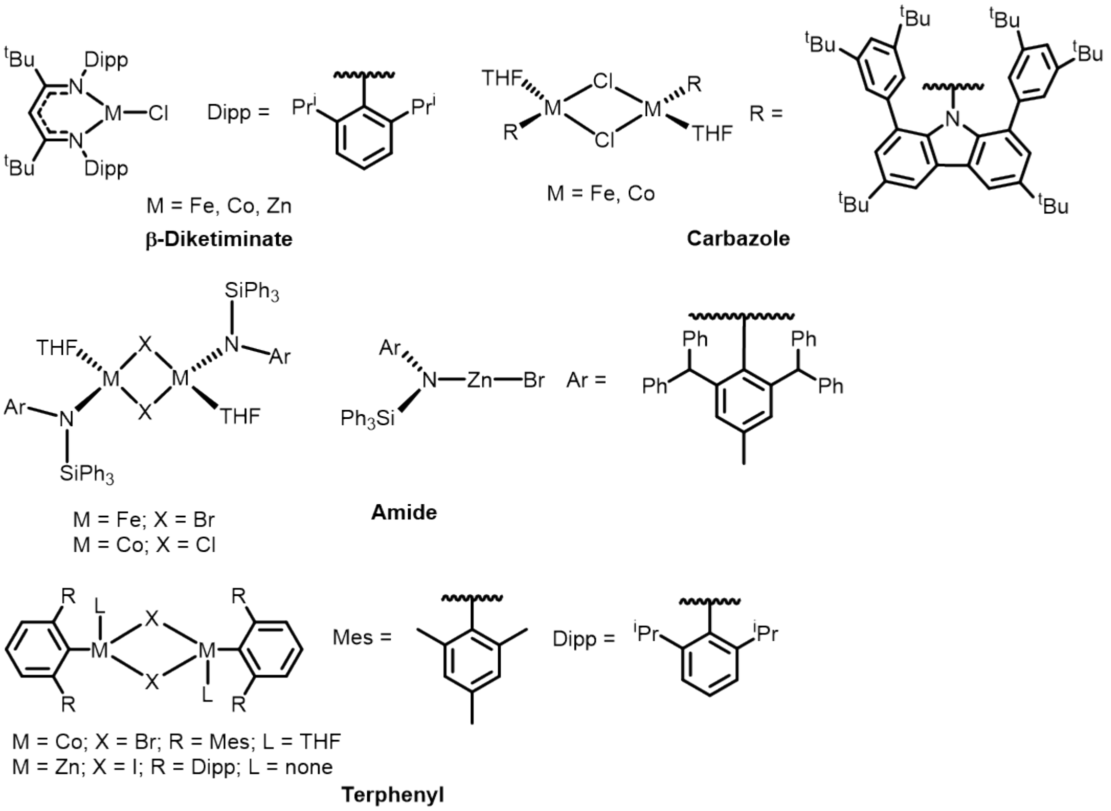

Metal	β-Diketiminate	Terphenyl	Amide	Carbazole
Fe	**66.3**		**50.0**	**45.3**
Co	**65.4**	**43.7**	**48.9**	**44.4**
Zn	**57.3**	**45.9**	**62.5**	

Table S13. Reported metal complex structures and buried volume table; The input file was obtained directly from the single-crystal X-ray diffraction data reported in the original publication.[6–12]

5. Computational Results

EPR Spectral Simulation and Fitting

Continuous-wave (CW) X-band EPR spectra were simulated and fitted using the EasySpin toolbox (MATLAB R2025a). The pepper solver was employed for powder CW spectra. Experimental data were recorded at 77 K on a Bruker EMX-plus spectrometer and loaded in Bruker BES3T format.

The spin Hamiltonian was defined as:

$$H = \mu_B \cdot B \cdot g \cdot S + S \cdot A \cdot I + H_{strain} + H_{lw}$$

Here, μ_B is the Bohr magneton; **B = (Bx, By, Bz)** is the external magnetic field vector; **g** is the 3 × 3 electronic g-tensor, reported through its principal values (g_x, g_y, g_z); and **S = (S_x, S_y, S_z)** is the electron spin operator for S = 1/2. The second term describes the hyperfine interaction, where **A** is the 3 × 3 hyperfine tensor with principal values (A_x, A_y, A_z) and **I = (I_x, I_y, I_z)** is the nuclear spin operator of each magnetically active nucleus. For **6** the nuclei included ^{14}N (I = 1). The additional terms H_{strain} and H_{lw} represent inhomogeneous and homogeneous broadening, respectively: H_{strain} is implemented in EasySpin as distributions of g and A values (g_{Strain}, H_{Strain}), while H_{lw} corresponds to the peak-to-peak linewidth (lw_{pp}) used to model relaxation broadening.

Experimental parameters were fixed to spectrometer settings: microwave frequencies of 9.468582 GHz for **6**, modulation amplitude 0.16 mT, and sweep widths of approximately 600 mT. The fitting procedure employed a simplex least-squares algorithm with a maximum of 200 iterations.

The g-tensor principal values, selected hyperfine A-tensor components, peak-to-peak linewidth (lw_{pp}), and strain terms (g_{Strain} and H_{Strain}) were allowed to vary within physically reasonable ranges. Convergence was assessed by minimizing the residual error and visual inspection of experimental versus simulated spectra.

DFT calculations

DFT calculations were carried out with the ORCA program package (versions 5.0.4).[13] Geometries of **6** was optimized with the TPSS-D4 functional using the def2-TZVPP basis set.[14,15] EPR parameters (g-tensors and hyperfine couplings) were computed at the optimized geometries with various functionals (BP86, PBE, TPSS, B3LYP, PBE0, TPSSh, TPSS0, and wB97X) and the CP(PPP) basis for metal atoms, in combination with def2-TZVPP for all non-metal atoms.[14] The eprnmr module was used with coupled-perturbed Kohn–Sham theory. Reported A-tensors are given in MHz and were rotated into the principal g-axis system using orca_euler. Auxiliary basis sets were generated automatically.[16]

Molecular orbital

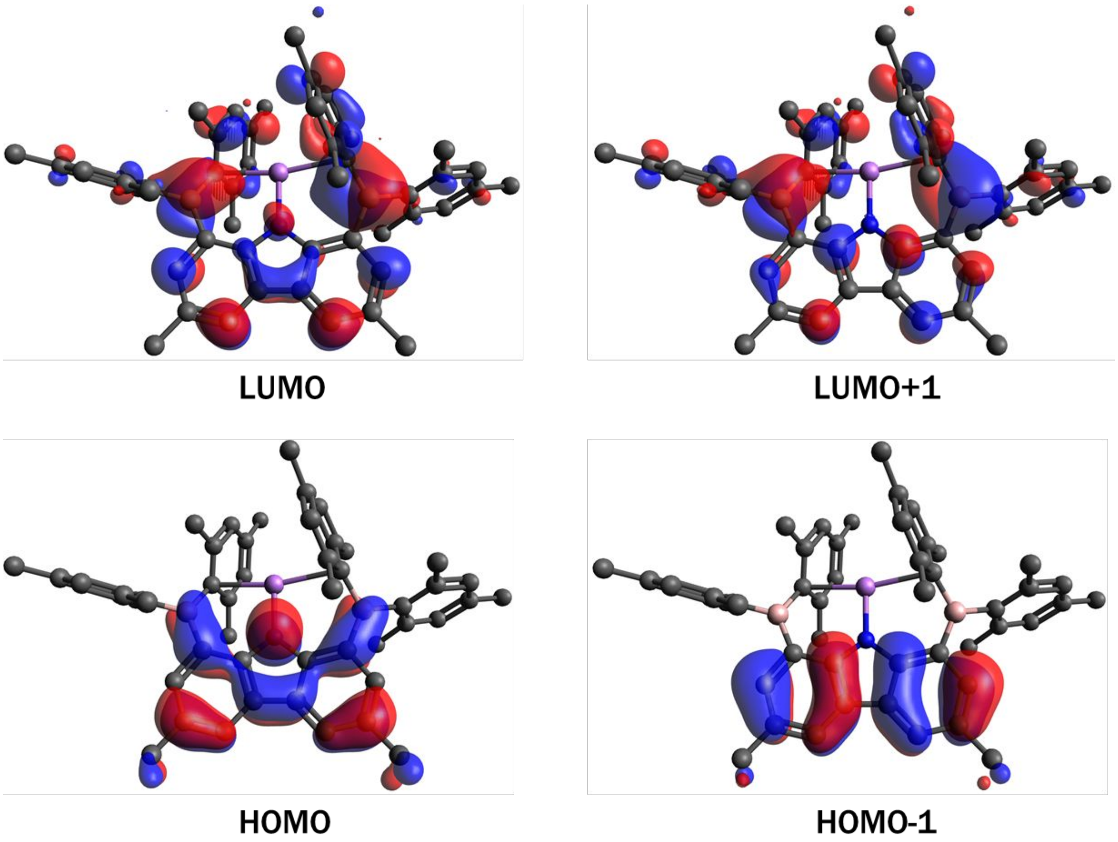

Figure S52. MO plot of the HOMO, HOMO-1, LUMO, and LUMO+1 of **LLi** (**1**) with isosurface value of 0.025.

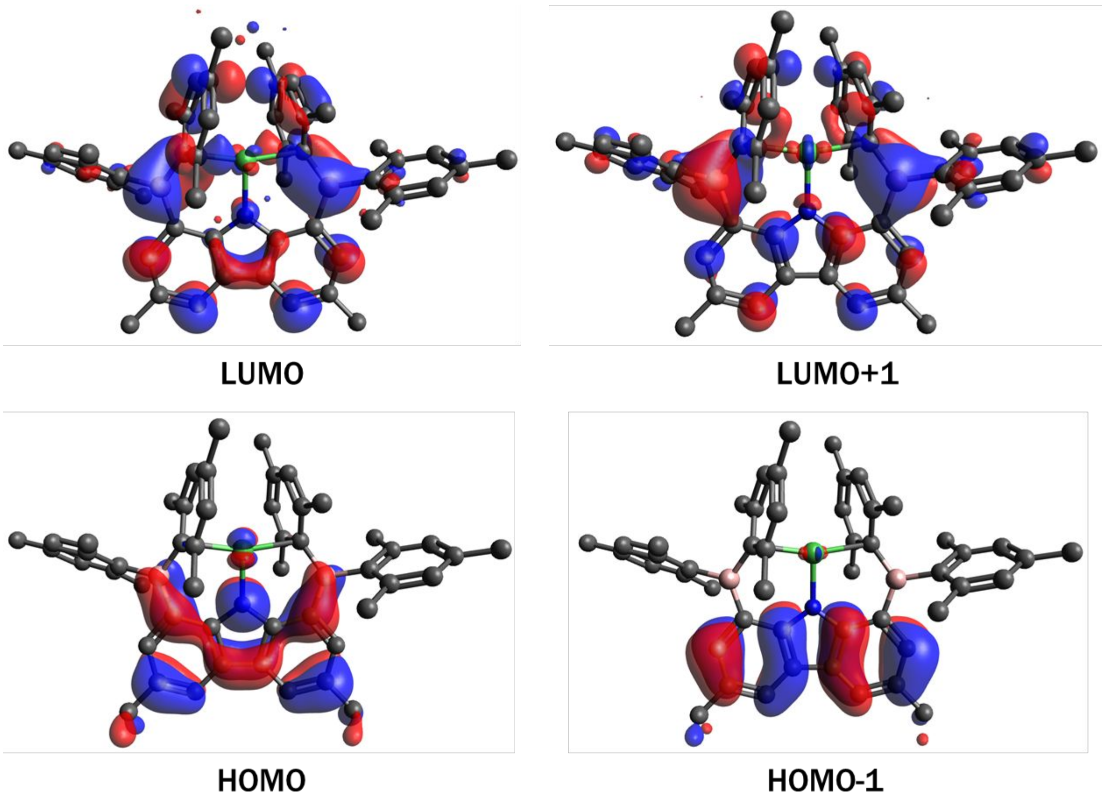

Figure S53. MO plot of the HOMO, HOMO-1, LUMO, and LUMO+1 of **LNi** (**6**) with isosurface value of 0.025.

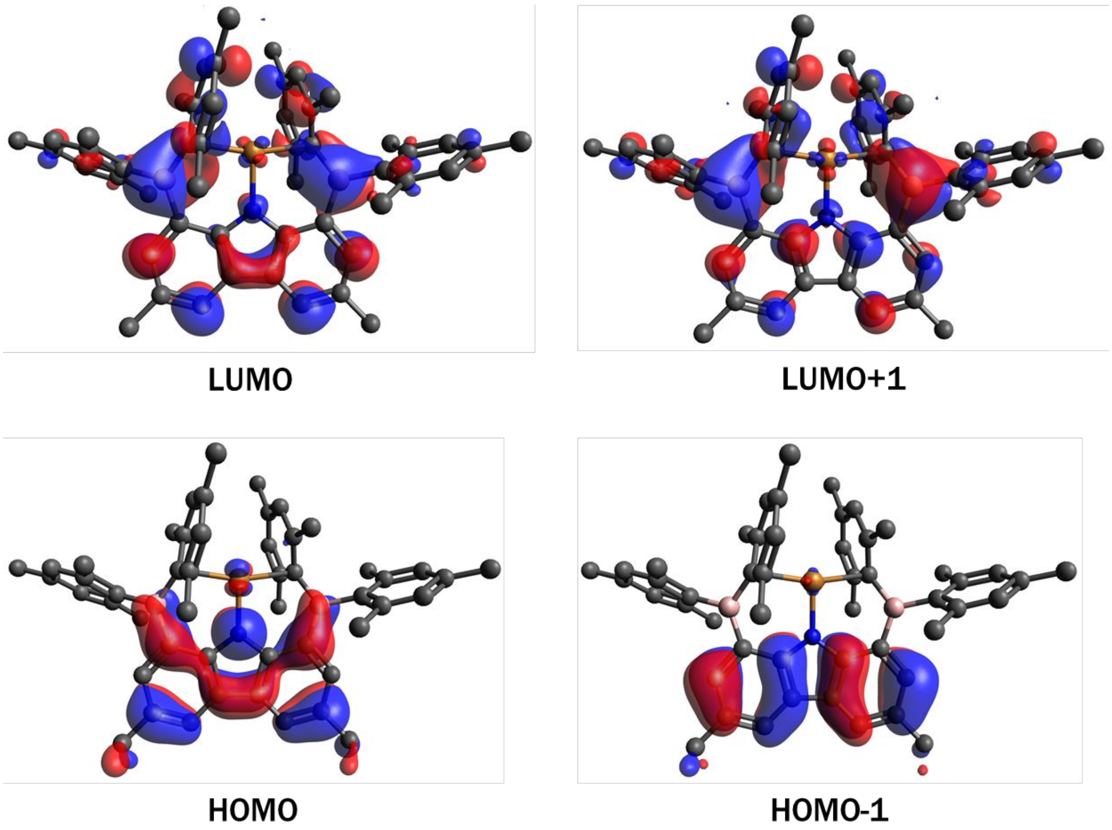

Figure S54. MO plot of the HOMO, HOMO-1, LUMO, and LUMO+1 of **LCu** (**7**) with isosurface value of 0.025.

Method	g_1	g_2	g_3	Metal $\|A_1\|$, $\|A_2\|$, $\|A_3\|$	Ligand $\|A_1\|$, $\|A_2\|$, $\|A_3\|$
Expt	1.98	2.25	2.50	0.5, 65, 60	0.5,40,60
BP86	2.01	2.13	2.21	81,80,147	40,28,27
PBE	2.01	2.13	2.21	79,81,149	40,29,28
TPSS	2.01	2.11	2.17	86,74,147	35,25,24
B3LYP	2.01	2.20	2.30	88,91,179	28,22,21
PBE0	2.02	2.21	2.33	78,101,191	25,19,18
TPSSh	2.01	2.13	2.21	85,83,166	29,22,21
TPSS0	2.02	2.17	2.26	81,94,187	22,17,16
wB97X	2.02	2.23	2.35	79,101,198	25,19,18

Table S14. Experimentally fitted and DFT calculated EPR g and A values for complex **6**.

Coordinate

Complex 1

C	12.2811	8.4151	6.4406
C	11.7418	8.157	7.7354
C	10.3073	8.4791	8.0945
H	9.7259	8.2809	7.3313
H	10.2319	9.4289	8.3227
H	10.0327	7.9359	8.8638
C	12.5115	7.5345	8.7058
H	12.1307	7.3765	9.5619
C	13.8164	7.1335	8.4684
C	14.6247	6.4253	9.5354
H	14.8268	5.512	9.2384
H	14.108	6.3907	10.3679
H	15.4602	6.9111	9.6906
C	14.3415	7.3798	7.2078
H	15.2363	7.1181	7.0267
C	13.6132	7.9898	6.2035
C	14.2883	8.195	4.858
H	13.8218	7.6717	4.1736
H	15.2215	7.9044	4.9134
H	14.2568	9.1457	4.6183
C	11.3107	8.458	3.8753
C	10.9702	7.1027	3.6766
C	10.5162	6.2314	4.8252
H	9.7349	6.6391	5.2558
H	11.2421	6.1459	5.4772
H	10.2751	5.3443	4.4858
C	11.0108	6.5541	2.391
H	10.7691	5.6419	2.2741
C	11.3906	7.2883	1.2841
C	11.4692	6.6781	-0.0846
H	10.5795	6.6846	-0.4976
H	11.788	5.7548	-0.0132
H	12.0902	7.1953	-0.6376
C	11.695	8.6267	1.4805
H	11.9413	9.1541	0.7303

C	11.653	9.2196	2.7267
C	12.0548	10.6861	2.819
H	12.6225	10.9179	2.0547
H	12.5495	10.8378	3.6515
H	11.2506	11.246	2.8114
C	10.6	10.3766	5.6274
C	9.3445	10.5521	5.0006
H	9.0886	9.9064	4.3515
C	8.4453	11.5944	5.2543
C	8.833	12.5656	6.1802
H	8.2479	13.2848	6.3872
C	10.0896	12.4819	6.8072
C	10.9597	11.3969	6.5494
C	10.7853	13.3384	7.718
C	10.4522	14.5318	8.3585
H	9.6084	14.9328	8.1884
C	11.3318	15.1423	9.2375
C	12.5748	14.5261	9.4251
H	13.1848	14.9501	10.0179
C	12.9998	13.3327	8.8129
C	12.041	12.7181	7.9435
C	15.2283	13.3697	10.3681
C	14.7408	13.2066	11.6809
C	13.4114	12.5591	11.9725
H	12.7013	13.2307	11.9117
H	13.4253	12.1778	12.8747
H	13.2453	11.8465	11.3195
C	15.5092	13.6296	12.7676
H	15.1735	13.4948	13.6448
C	16.7346	14.2335	12.6159
C	17.5479	14.6769	13.8186
H	18.4937	14.7457	13.5672
H	17.4495	14.0198	14.5396
H	17.2284	15.5504	14.1253
C	17.201	14.4269	11.3281
H	18.0346	14.8636	11.2022
C	16.4839	14.0022	10.209
C	17.0636	14.2933	8.8366

H	17.3786	13.4588	8.4305
H	17.8144	14.9165	8.9243
H	16.3721	14.692	8.2678
C	15.1705	11.7628	8.1713
C	15.7137	10.5704	8.707
C	15.3697	10.113	10.1047
H	14.4151	9.8978	10.1522
H	15.5762	10.8284	10.7425
H	15.896	9.316	10.3263
C	16.5334	9.7666	7.9121
H	16.8897	8.9711	8.2886
C	16.8505	10.0765	6.6099
C	17.7822	9.2116	5.795
H	17.3562	8.9883	4.9399
H	17.9775	8.3874	6.287
H	18.6154	9.698	5.6267
C	16.2795	11.2262	6.0756
H	16.4641	11.4465	5.1707
C	15.4508	12.0673	6.8127
C	14.8915	13.2911	6.1207
H	15.547	13.6283	5.4734
H	14.7035	13.985	6.7864
H	14.0637	13.0525	5.6532
B	11.395	9.1012	5.3198
B	14.4194	12.8158	9.1063
N	12.1553	11.5266	7.2454
Li	13.5239	10.2267	7.3975
C	10.99626	16.42171	10.02624
H	10.45588	17.09741	9.39669
H	10.39722	16.16986	10.87631
H	11.90253	16.88681	10.35374
C	7.10604	11.67849	4.49871
H	7.27397	12.07182	3.5179
H	6.67756	10.701	4.42245
H	6.43607	12.32108	5.03079

Complex 6

C	-1.900458996	-0.841072221	-0.772045304
C	-1.897853841	-2.093717982	-0.052210570
C	-2.577418552	-2.228944972	1.294779756
H	-2.553536734	-1.288105786	1.863613937
H	-2.082639162	-3.006928209	1.898661233
H	-3.637420178	-2.514056740	1.169254860
C	-1.387553360	-3.257615154	-0.658440780
H	-1.447448093	-4.203081709	-0.106058858
C	-0.840108152	-3.254549231	-1.953683632
C	-0.357464011	-4.535408742	-2.588337324
H	0.478678348	-4.977024853	-2.013180643
H	-0.007158121	-4.367803204	-3.619888546
H	-1.161002556	-5.293579016	-2.615647078
C	-0.719017427	-2.020073649	-2.604304110
H	-0.260532324	-1.982296773	-3.599523615
C	-1.201594622	-0.815922787	-2.043559701
C	-1.139155382	0.432249391	-2.901593099
H	-2.128543125	0.912373920	-2.991160871
H	-0.778878192	0.181509606	-3.912614499
H	-0.461841030	1.186535415	-2.469011150
C	-4.381689927	-0.069221567	-0.000190288
C	-5.167454601	-0.851940049	-0.892471033
C	-4.618939544	-1.318353157	-2.226647224
H	-5.430969481	-1.686791885	-2.875914550
H	-3.890855566	-2.141119094	-2.101297904
H	-4.098647568	-0.507033596	-2.764052908
C	-6.482598622	-1.202819199	-0.549526028
H	-7.077276811	-1.791794857	-1.259814259
C	-7.054004101	-0.827856373	0.677957404
C	-8.478584555	-1.199644821	1.019754567
H	-8.630039458	-1.259737689	2.111077527
H	-8.760338639	-2.169804651	0.575057090
H	-9.187274264	-0.443366895	0.630154258
C	-6.265686742	-0.077651530	1.564128544
H	-6.681440308	0.207359020	2.539291983
C	-4.955507224	0.316968845	1.243981421
C	-4.180371270	1.115268562	2.276261431

H	-4.248607520	2.200193036	2.079492639
H	-3.104650958	0.870604957	2.276707582
H	-4.579804572	0.929909642	3.288171718
C	-2.453507076	1.794579284	-0.315987286
C	-3.393400142	2.851812417	-0.434844093
H	-4.449603265	2.582022463	-0.560904372
C	-3.044336268	4.213635832	-0.415089953
C	-1.686104844	4.559945040	-0.236242744
H	-1.396055937	5.617616272	-0.198794340
C	-0.716104362	3.562687544	-0.102082703
C	-1.096480346	2.178992602	-0.154334164
C	1.095671155	2.179300372	0.153960111
C	0.714926622	3.562893995	0.101494031
C	1.684654878	4.560435366	0.235465871
H	1.394318094	5.618021771	0.197839450
C	3.042965694	4.214528680	0.414549836
C	3.392376484	2.852809819	0.434669777
H	4.448638277	2.583358483	0.560939055
C	2.452795962	1.795285527	0.315732382
C	4.381663893	-0.067734173	0.000278172
C	4.956053230	0.319415162	-1.243327436
C	4.181276223	1.118330539	-2.275450941
H	3.105279336	0.874967873	-2.275266020
H	4.579968699	0.932270574	-3.287540038
H	4.250958632	2.203225500	-2.079026242
C	6.266450197	-0.074782985	-1.563131440
H	6.682680328	0.211065144	-2.537849498
C	7.054331755	-0.825752782	-0.677223698
C	8.479093927	-1.197182508	-1.018663838
H	9.187387923	-0.440054808	-0.629987716
H	8.630538030	-1.258419623	-2.109932904
H	8.761401989	-2.166674610	-0.572882528
C	6.482181654	-1.202063613	0.549507222
H	7.076396121	-1.791914601	1.259454448
C	5.166840348	-0.851529468	0.892092274
C	4.617419094	-1.319799156	2.225234186
H	4.097020181	-0.509152623	2.763560392
H	5.428961395	-1.689453151	2.874408275

H	3.889128659	-2.142157406	2.098217946
C	1.900764739	-0.840508882	0.771939750
C	1.898879834	-2.093264122	0.052422374
C	2.578723168	-2.228436465	-1.294438375
H	2.554652102	-1.287683828	-1.863397935
H	2.084382611	-3.006686712	-1.898331172
H	3.638811093	-2.513097991	-1.168611314
C	1.389097150	-3.257269498	0.658856689
H	1.449599396	-4.202845270	0.106726062
C	0.841430854	-3.254145562	1.954006501
C	0.359191280	-4.535080971	2.588809015
H	1.162427322	-5.293609593	2.614604848
H	-0.477931950	-4.976088489	2.014586569
H	0.010284835	-4.367801987	3.620886775
C	0.719629262	-2.019562105	2.604303845
H	0.260961254	-1.981743638	3.599439458
C	1.201795281	-0.815345680	2.043351749
C	1.138960075	0.433013338	2.901106825
H	0.462237281	1.187450904	2.467900409
H	2.128466957	0.912806042	2.991304487
H	0.778014228	0.182529060	3.911951739
B	-2.893444502	0.325478906	-0.349619489
B	2.893219660	0.326386324	0.349395542
N	-0.000299481	1.356219638	-0.000089321
Ni	0.000008329	-0.574631139	-0.000207275
C	4.091028210	5.292587459	0.587271157
H	3.998691423	6.074992220	-0.188043580
H	3.994756487	5.796690927	1.568084065
H	5.108952238	4.872002695	0.527549050
C	-4.092684658	5.291371038	-0.588091453
H	-3.999691338	6.074701138	0.186200285
H	-3.997408708	5.794314918	-1.569601953
H	-5.110509197	4.870748774	-0.526926839
He	0.000000000	0.000000000	0.000000000
Ne	0.000182555	-0.793765790	-0.000046547
Ar	-0.024047270	0.000040995	-0.793401470
Kr	0.793401450	0.000183882	-0.024047260

Complex 7

C	5.0973	16.0044	5.1771
C	5.098	17.4228	5.2106
C	4.8706	18.2349	3.9428
H	5.3255	17.8014	3.1911
H	5.2322	19.1391	4.0642
H	3.9096	18.289	3.7573
C	5.1445	18.1087	6.4296
H	5.1329	19.0574	6.4198
C	5.2077	17.4562	7.6416
C	5.2413	18.2073	8.9442
H	5.4442	19.1493	8.7727
H	5.9338	17.8243	9.523
H	4.3693	18.1332	9.385
C	5.2496	16.0639	7.6179
H	5.3108	15.6008	8.4449
C	5.2061	15.3182	6.4421
C	5.2347	13.8222	6.5467
H	4.516	13.4436	5.9986
H	5.1065	13.5585	7.4812
H	6.0988	13.4895	6.2287
C	3.3722	15.5992	3.099
C	2.1426	15.7859	3.7434
C	1.9913	15.5395	5.2343
H	1.0397	15.491	5.4617
H	2.4099	16.2747	5.7294
H	2.4289	14.6945	5.4714
C	1.0064	16.1484	3.0167
H	0.1825	16.2722	3.47
C	1.0626	16.3332	1.6388
C	-0.1871	16.7011	0.8633
H	0.0468	16.8594	-0.0753
H	-0.5798	17.5154	1.2427
H	-0.8349	15.9684	0.9191
C	2.2692	16.1805	1.0181
H	2.3248	16.3411	0.0837
C	3.4233	15.7999	1.7001
C	4.7067	15.6774	0.9066

H	4.8198	14.7507	0.6081
H	5.4656	15.9352	1.47
H	4.6648	16.2696	0.1255
C	5.443	13.921	3.4128
C	4.8027	12.8784	2.7294
H	3.8621	12.9432	2.6109
C	5.4412	11.7484	2.2078
C	6.8288	11.6734	2.3208
H	7.2944	10.9315	1.9526
C	7.5358	12.6861	2.9763
C	6.8416	13.7808	3.5398
C	8.9813	14.163	3.9526
C	8.9342	12.9309	3.2413
C	10.116	12.2634	2.9498
H	10.0885	11.4549	2.4533
C	11.3479	12.7752	3.3836
C	11.3426	13.9715	4.1032
H	12.176	14.2988	4.4198
C	10.1953	14.729	4.3961
C	11.5187	16.4414	6.0446
C	11.8651	15.5944	7.1297
C	11.0961	14.3432	7.4491
H	10.1487	14.48	7.2371
H	11.1911	14.1354	8.4017
H	11.4469	13.5993	6.9149
C	12.9079	15.9518	7.9805
H	13.1144	15.3863	8.7141
C	13.6511	17.0942	7.7964
C	14.8104	17.4567	8.7127
H	15.6076	16.9513	8.4463
H	14.5769	17.2346	9.6388
H	14.9933	18.4166	8.643
C	13.3261	17.9206	6.7378
H	13.828	18.7153	6.6039
C	12.2779	17.6209	5.855
C	12.0287	18.5744	4.7225
H	11.8424	18.0669	3.9038
H	12.8214	19.134	4.5858

H	11.2592	19.1416	4.9373
C	9.2211	17.2586	4.8745
C	8.7086	18.0301	5.9386
C	8.8802	17.5879	7.3891
H	8.9309	16.6092	7.4282
H	8.1159	17.8958	7.9177
H	9.7059	17.9724	7.7518
C	8.1289	19.2821	5.7002
H	7.8009	19.785	6.4366
C	8.0205	19.809	4.4282
C	7.4587	21.1819	4.1771
H	8.1949	21.8196	4.0697
H	6.8996	21.4495	4.9359
H	6.9177	21.1686	3.3598
C	8.4482	19.0206	3.364
H	8.3446	19.351	2.4798
C	9.0227	17.772	3.5495
C	9.4803	17.0276	2.3236
H	8.8538	16.2977	2.1311
H	10.3733	16.6577	2.4812
H	9.5108	17.6431	1.5607
B	4.6715	15.166	3.8898
B	10.2894	16.0912	5.1046
N	7.712	14.6705	4.1241
Cu	7.2337	16.3388	4.9183
C	12.65357	12.02645	3.05771
H	12.68788	11.11162	3.6116
H	12.68913	11.81077	2.01028
H	13.49089	12.63674	3.32482
C	4.61158	10.67698	1.4761
H	4.45693	10.97742	0.46085
H	5.13602	9.74453	1.49618
H	3.66537	10.56457	1.96287

6. References

(1) Falivene, L.; Credendino, R.; Poater, A.; Petta, A.; Serra, L.; Oliva, R.; Scarano, V.; Cavallo, L. SambVca 2. A Web Tool for Analyzing Catalytic Pockets with Topographic Steric Maps. *Organometallics* **2016**, *35*, 2286–2293.

(2) Moorhouse, R. S.; Moxey, G. J.; Ortu, F.; Reade, T. J.; Lewis, W.; Blake, A. J.; Kays, D. L. Structural Diversity in Alkali Metal Complexes of Sterically Demanding Carbazol-9-Yl Ligands. *Inorg. Chem.* **2013**, *52*, 2678–2683.

(3) Yu, Q.; Zhang, L.; He, Y.; Pan, J.; Li, H.; Bian, G.; Chen, X.; Tan, G. A Stable Tetrazagallole and Its Radical Anion Dimer. *Chem. Commun.* **2021**, *57*, 9268–9271.

(4) Kaiser, M.; Müller, M. P.; Krätschmer, F.; Rutschmann, M.; Hinz, A. Efficient Fluorescence of Alkali Metal Carbazolides. *Inorg. Chem. Front.* **2023**, *10*, 2987–2994.

(5) Coombs, N. D.; Stasch, A.; Cowley, A.; Thompson, A. L.; Aldridge, S. Bulky Aryl Functionalized Carbazolyl Ligands: Amido Alternatives to the 2,6-Diarylphenyl Ligand Class? *Dalton Trans.* **2008**, 332–337.

(6) Ellison, J. J.; Power, P. P. Synthesis and Structural Characterization of Magnesium and Cobalt Derivatives of the Bulky Aryl Ligand $-C_6H_3$-2,6-Mes_2. *J. Organomet. Chem.* **1996**, *526*, 263–267.

(7) Smith, J. M.; Lachicotte, R. J.; Holland, P. L. Tuning Metal Coordination Number by Ancillary Ligand Steric Effects: Synthesis of a Three-Coordinate Iron(II) Complex. *Chem. Commun.* **2001**, 1542–1543.

(8) Holland, P. L.; Cundari, T. R.; Perez, L. L.; Eckert, N. A.; Lachicotte, R. J. Electronically Unsaturated Three-Coordinate Chloride and Methyl Complexes of Iron, Cobalt, and Nickel. *J. Am. Chem. Soc.* **2002**, *124*, 14416–14424.

(9) Ayala, C. N.; Chisholm, M. H.; Gallucci, J. C.; Krempner, C. Chemistry of BDI*M(2+) Complexes (M = Mg, Zn) and Their Role in Lactide Polymerization Where BDI* Is the Anion Derived from Methylenebis(C-^{t}Bu, N-2,6-Diisopropylphenyl)Imine BDI*H. *Dalton Trans.* **2009**, 9237.

(10) Kaiser, M.; Winkler, L.; Hinz, A. Complexes of 3d Metals with a Bulky Carbazolyl Ligand. *Z. Anorg. Allg. Chem.* **2022**, *648*, e202200131.

(11) Hicks, J.; Jones, C. Extremely Bulky Amido First Row Transition Metal(II) Halide Complexes: Potential Precursors to Low Coordinate Metal–Metal Bonded Systems. *Inorg. Chem.* **2013**, *52*, 3900–3907.

(12) Zhu, Z.; Brynda, M.; Wright, R. J.; Fischer, R. C.; Merrill, W. A.; Rivard, E.; Wolf, R.; Fettinger, J. C.; Olmstead, M. M.; Power, P. P. Synthesis and Characterization of the Homologous M−M Bonded Series Ar‘MMAr‘ (M = Zn, Cd, or Hg; Ar‘ = C_6H_3-2,6-(C_6H_3-2,6-Pr^i_2)$_2$) and Related Arylmetal Halides and Hydride Species. *J. Am. Chem. Soc.* **2007**, *129*, 10847–10857.

(13) Neese, F. Software Update: The ORCA Program System—Version 5.0. *WIREs Comput Mol Sci* **2022**, *12*, e1606.
(14) Weigend, F.; Ahlrichs, R. Balanced Basis Sets of Split Valence, Triple Zeta Valence and Quadruple Zeta Valence Quality for H to Rn: Design and Assessment of Accuracy. *Phys. Chem. Chem. Phys.* **2005**, *7*, 3297.
(15) Tao, J.; Perdew, J. P.; Staroverov, V. N.; Scuseria, G. E. Climbing the Density Functional Ladder: Nonempirical Meta–Generalized Gradient Approximation Designed for Molecules and Solids. *Phys. Rev. Lett.* **2003**, *91*, 146401.
(16) Stoychev, G. L.; Auer, A. A.; Neese, F. Automatic Generation of Auxiliary Basis Sets. *J. Chem. Theory Comput.* **2017**, *13*, 554–562.
